# Copper‐Catalyzed Oxidative Cross‐Coupling of Electron‐Deficient Polyfluorophenylboronate Esters with Terminal Alkynes

**DOI:** 10.1002/chem.202002888

**Published:** 2020-11-09

**Authors:** Zhiqiang Liu, Yudha P. Budiman, Ya‐Ming Tian, Alexandra Friedrich, Mingming Huang, Stephen A. Westcott, Udo Radius, Todd B. Marder

**Affiliations:** ^1^ Institute of Inorganic Chemistry and Institute for Sustainable Chemistry & Catalysis with Boron Julius-Maximilians-Universität Würzburg Am Hubland 97074 Würzburg Germany; ^2^ Department of Chemistry and Biochemistry Mount Allison University Sackville NB E4L 1G8 Canada; ^3^ Department of Chemistry Faculty of Mathematics and Natural Sciences Universitas Padjadjaran 45363 Jatinangor Indonesia

**Keywords:** boronate esters, coupling reactions, fluorine, fluoroarenes, Sonogashira reaction

## Abstract

We report herein a mild procedure for the copper‐catalyzed oxidative cross‐coupling of electron‐deficient polyfluorophenylboronate esters with terminal alkynes. This method displays good functional group tolerance and broad substrate scope, generating cross‐coupled alkynyl(fluoro)arene products in moderate to excellent yields. Thus, it represents a simple alternative to the conventional Sonogashira reaction.

## Graphical abstract

**BFF**: A mild procedure is reported for the synthesis of alkynyl(fluoro)arenes via copper‐catalyzed oxidative cross‐coupling of polyfluorophenylboronate esters with terminal alkynes.

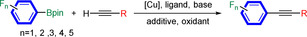

## Introduction

Functionalized aryl and heteroaryl alkynes are powerful building blocks in chemical synthesis because of their versatility to be transformed into various useful molecules and also their ubiquity in natural product synthesis, pharmaceuticals, and advanced materials.^[1]^ Consequently, much effort has been expended to develop efficient methods to install various alkynyl groups. Some of the strategies which have been established include: 1) Sonogashira palladium/copper‐catalyzed sp^2^‐sp cross‐coupling of aryl halides with terminal alkynes;^[2]^ 2) direct alkynylation of unreactive alkyl and aryl C−H bonds with prefunctionalized alkynating reagents such as alkynyl halides^[3]^ and hypervalent iodine reagents;^[4]^ 3) alkynylation of tetra‐ and pentafluoroarenes and heteroarenes via C−H bond activation;[^5^, ^6^] and 4) cross‐coupling of copper(I) acetylides with aryl halides, known as the Castro‐Stephens reaction.[^7^, ^8^, ^9^] However, some drawbacks remain, such as the use of precious metal catalysts including Pd,^[2]^ Rh,[^4a^, ^4b^, ^4h^] and Au,[^4c^, ^4d^] strategies that depend on the use of alkynyl halides or hypervalent iodine reagents, which are less readily available than the corresponding terminal alkynes, and the fact that copper(I) acetylides can be heat and shock sensitive when isolated.

**Scheme 1:**
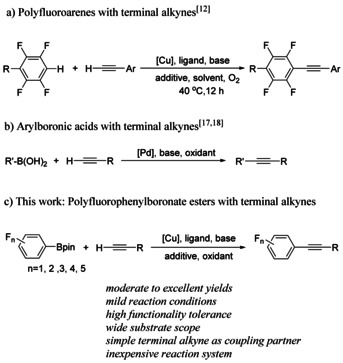
Selected oxidative cross‐coupling reactions of alkynes.

It is generally acknowledged that polyfluoroarenes are important fluorinated aromatic cores and key structural units for various organic molecules, such as pharmaceuticals, agrochemicals and organic materials.^[10]^ The development of efficient methods to introduce fluorine or fluorinated building blocks into organic molecules has been the subject of intense research. Under certain conditions, Sonogashira cross‐couplings involving highly fluorinated aryl halides can be problematic, giving low yields^[11a]^ and side reactions, that is, hydrodehalogenation accompanied by homocoupling of the terminal alkyne.^[11b]^ The latter problem seems to arise from the slow reductive elimination of the fluoroaryl alkyne from Pd^II^, which leads to competing reverse transmetalation processes, that is, transfer of aryl groups from Pd to Cu in exchange for a second alkynyl moiety being transferred from Cu to Pd. Thus, an alternative approach would be useful. In 2010, Su and co‐workers demonstrated the direct functionalization of polyfluoroarene C−H bonds with terminal alkynes, which has proven to be a viable method to generate the corresponding alkynylated products (Scheme 1 a),^[12]^ but this reaction is limited to C_6_F_5_H or 4‐RC_6_F_4_H substrates. Soon after, the oxidative alkynylation of azoles containing acidic C−H bonds with terminal alkynes was reported by the groups of Miura,^[13]^ Chang,^[14]^ and others.^[15]^ Recently, Su and co‐workers reported a palladium‐catalyzed alkynylation of heterocyclic substrates such as thiophenes and furans.^[16]^ Although these achievements were promising, they were restricted by elevated temperatures (>90 °C) and limited substrate scope. In 2003, the palladium‐catalyzed oxidative cross‐coupling of terminal alkynes with arylboronic acids was first disclosed by Zou and co‐workers (Scheme 1 b).^[17]^ In the past few years, various modifications of this Pd‐catalyzed reaction have been developed.^[18]^ However, palladium is costly and only a few electron‐withdrawing substituents on the aromatic ring of arylboronic acids were employed. Recently, Cheng et al. disclosed a copper‐catalyzed oxidative coupling of arylboronic acids with terminal alkynes.^[19]^ However, the reported method suffers from some disadvantages including high reaction temperature, long reaction time (36 h), and only moderate yields. From a synthetic point of view, the development of an improved procedure employing an inexpensive catalyst for widespread application has remained a highly desirable goal.

We reported the C−F borylation of fluoroarenes using a NHC (*N*‐heterocyclic carbene)‐ligated Ni complex as a catalyst to generate fluorinated arylboronic acid pinacol esters (Ar_F_Bpin) in good to excellent yields.[^20a^, ^20b^] Very recently, we reported optimized conditions for the Suzuki–Miyaura cross‐coupling of Ar_F_Bpin with aryl iodides and bromides using a combination of CuI and phenanthroline as a catalyst precursor to generate cross‐coupled products in moderate to excellent yields.^[20c]^ We have recently reported the palladium‐catalyzed homocoupling of fluorinated arylboronates,^[20d]^ and the borylation of aryl chlorides, using NHC‐stabilized nickel(0) complexes^[20e]^ or a readily prepared NHC‐stabilized Cu catalyst.^[20f]^ Inspired by these results, we attempted to develop a Cu‐catalyst system for the oxidative cross‐coupling of Ar_F_Bpin compounds with terminal alkynes.

## Results and Discussion

We initially investigated the cross‐coupling reaction with model substrates pentafluorophenyl‐Bpin (**1 a**) and phenylacetylene (**2 a**), using Ag_2_O as the oxidant and phenanthroline (Phen) as the ligand. During our initial experiments, no reaction occurred when CuBr_2_ was employed as the metal source, with *t*BuOLi as the base in DMF solution (Table 1, entry 1). However, employing CuCl as catalyst precursor gave rise to compound **3 a** in 10 % yield (Table 1, entry 2). The introduction of Cu(OAc)_2_ as the catalyst precursor improved the yield to 18 % (Table 1, entry 3). However, large amounts of diyne byproduct **4** and perfluorobiphenyl compound **5** were produced.

**Table 1:** Optimization of reaction conditions.^[a]^

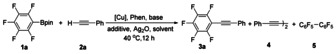							
Entry	[Cu]	Base	Additive	Solvent	Yield [%]^[b]^		
	(15 mol %)	(2 equiv)	(40 mol %)		**3 a**	**4**	**5**
1	CuBr_2_	*t*BuOLi	–	DMF	0	65	36
2	CuCl	*t*BuOLi	–	DMF	10	55	35
3	Cu(OAc)_2_	*t*BuOLi	–	DMF	18	60	35
4	Cu(OAc)_2_	K_3_PO_4_	–	DMF	35	52	8
5	Cu(OAc)_2_	K_3_PO_4_	DDQ	DMF	58	5	8
6	Cu(OAc)_2_	Cs_2_CO_3_	DDQ	DMF	11	25	25
**7**	**Cu(OAc)_2_**	**K_2_CO_3_**	**DDQ**	**DMF**	**82**	**3**	**4**
8	Cu(OAc)_2_	KF	DDQ	DMF	15	10	45
9	Cu(OAc)_2_	K_2_CO_3_	DDQ	MTBE	0	5	35
10	Cu(OAc)_2_	K_2_CO_3_	DDQ	DCE	0	5	0
11	Cu(OAc)_2_	K_2_CO_3_	DDQ	toluene	0	10	10
12	Cu(OAc)_2_	K_2_CO_3_	DDQ	DMSO	25	15	20
13	Cu(OAc)_2_	K_2_CO_3_	DDQ	CH_3_CN	10	15	10
14	Cu(OAc)_2_	K_2_CO_3_	DDQ	THF	5	10	15
15^[c]^	Cu(OAc)_2_	K_2_CO_3_	DDQ	DMF	5	5	40
16^[d]^	Cu(OAc)_2_	K_2_CO_3_	DDQ	DMF	5	10	35
17^[e,f]^	Cu(OAc)_2_	K_2_CO_3_	DDQ	DMF	0	6	10
18^[g]^	Cu(OAc)_2_	K_2_CO_3_	DDQ	DMF	35	5	30
19^[h]^	Cu(OAc)_2_	K_2_CO_3_	DDQ	DMF	25	28	30

[a] Reaction conditions: **1 a** (0.4 mmol), **2 a** (0.45 mmol), Cu(OAc)_2_ (15 mol %), phenanthroline (Phen, 15 mol %), Ag_2_O (1.8 equiv), DDQ (40 mol %), base (2.0 equiv), anhydrous and degassed solvent (5 mL). The mixture was stirred at 40 °C under argon, in a sealed tube for 12 h. [b] **3 a**: isolated yield, **4**: isolated yield, **5**: the yields were determined by GC‐MS analysis vs. a calibrated internal standard (*n*‐dodecane) and are averages of two runs. [c] The reaction was performed in air. [d] Room temperature. [e] In the absence of Ag_2_O. [f] Under O_2._ [g] Ag_2_O (1.2 equiv). [h] Base (1.0 equiv).

We speculated that strong bases, such as *t*BuOLi, might accelerate the formation of **5**. Under otherwise identical conditions, replacing the strong base with K_3_PO_4_ effectively inhibited the homocoupling of pentafluorophenyl‐Bpin (Table 1, entry 4). To our surprise, the addition of 2,3‐dichloro‐5,6‐dicyanobenzoquinone (DDQ) significantly improved the yield to 58 % and suppressed the formation of **4** (Table 1, entry 5). It is possible that DDQ serves as an electron‐transfer mediator.[^12^, ^21^] To optimize the reaction performance, we screened the reaction parameters, including the base and the solvent. Of the bases examined, K_2_CO_3_ proved to be the most effective (entry 7). Both KF and Cs_2_CO_3_ gave significantly lower yields (entries 6 and 8). In addition, reaction optimization also revealed that the solvent had a significant impact on this reaction. Lower yields were observed when reactions were performed in other solvents such as 1,2‐dichloroethane (DCE), CH_3_CN, THF, DMSO, methyl *tert*‐butyl ether (MTBE), and toluene (entries 9–14). Notably, the replacement of Ag_2_O with O_2_ failed to give any desired product (entry 17), indicating the unique roles of Ag_2_O in promoting this reaction. Attempts to run the reaction in air resulted in a very low yield of the desired product (entry 15). Reducing the amount of K_2_CO_3_ and Ag_2_O also diminished the yield (Table 1, entries 18 and 19).

**Scheme 2:**
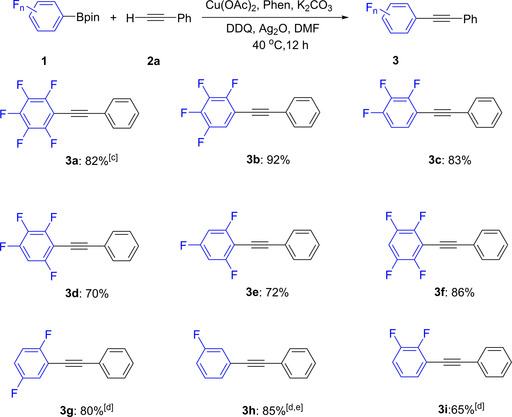
Scope of the reaction with respect to the different polyfluorophenyl boronate substrates **1**.^[a,b]^ [a] Reaction conditions: **1** (0.4 mmol), **2 a** (0.45 mmol), Cu(OAc)_2_ (15 mol %), Phen (15 mol %), Ag_2_O (1.2 equiv), DDQ (40 mol %), K_2_CO_3_ (2.0 equiv), DMF (4 mL), 40 °C, 12 h, Ar. [b] **3**: isolated yield. [c] Ag_2_O (1.8 equiv). [d] 80 °C. [e] *t*BuOLi.

With the optimized conditions in hand, we focused our attention on investigating the scope and limitations of the oxidative cross‐coupling reaction. As shown in Scheme 2, various fluorophenylboronate esters **1** containing 1–4 fluorine atoms were tested. Under the standard conditions (Table 1, entry 7), different tetrafluorophenylboronate esters and trifluorophenylboronate esters smoothly underwent alkynylation giving good to excellent yields (Scheme 2, **3 b**–**3 f**). However, these reaction conditions were not suitable for Ar_F_Bpin substrates containing di‐ or mono‐fluorinated arylboronates such as 2,5‐ or 2,3‐difluorophenyl‐Bpin (**1 g** and **1 i**) and 3‐fluorophenyl‐Bpin (**1 h**), perhaps due to the lower Lewis acidity of the boronates which is impacted by the number of fluorines and, especially, *ortho*‐fluorine substituents. We speculated that increasing the temperature might be crucial for overcoming the barrier to C−B bond activation and thus to obtaining efficient catalysis. When reactions were performed at 80 °C, the corresponding products **3 g** and **3 i** were formed in good yields. It is also noteworthy that replacement of the weak base with a stronger base afforded the corresponding product in good yield (**3 h**).

**Scheme 3:**
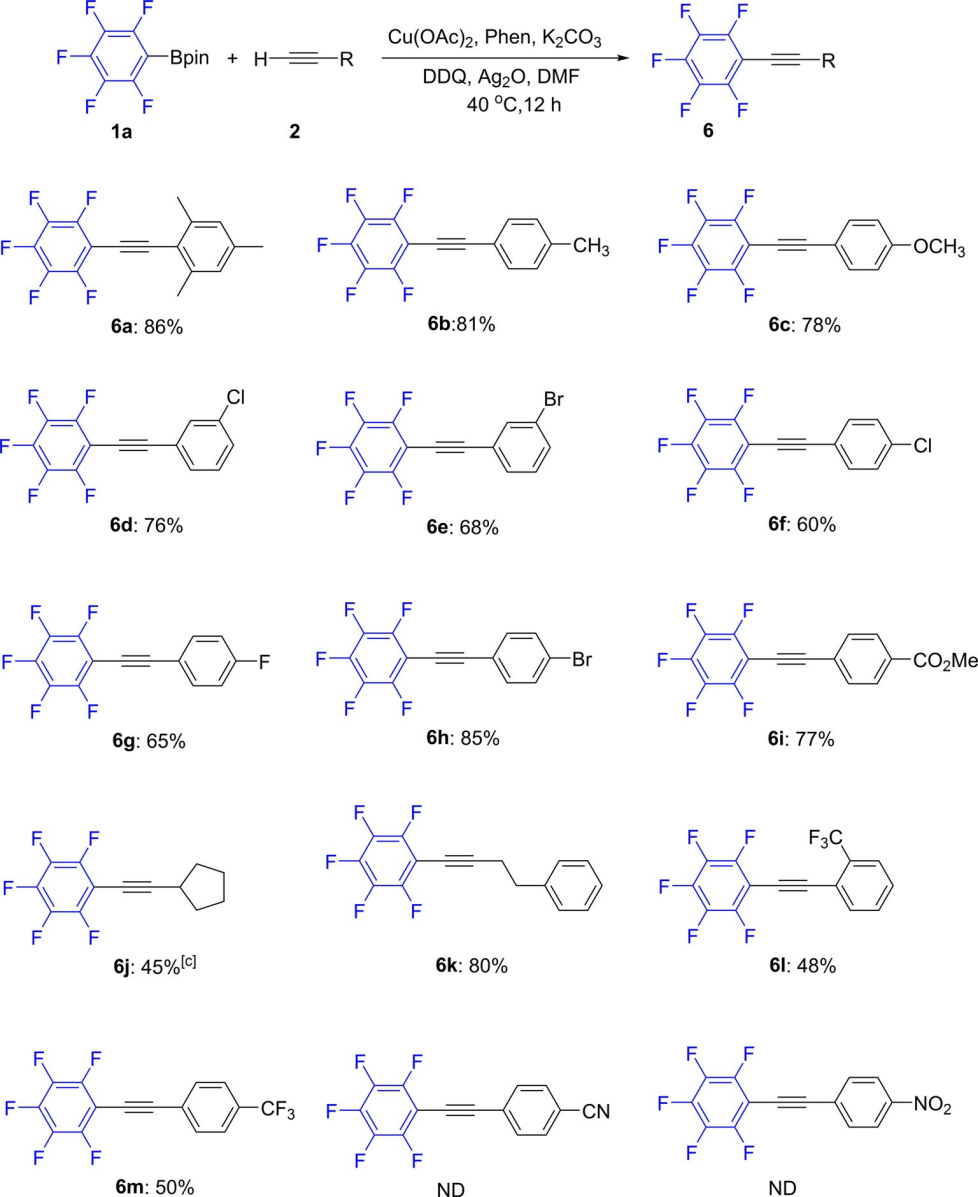
Scope of the reaction with respect to the different terminal alkyne substrates **2**.^[a,b]^ [a] Reaction conditions: **1 a** (0.4 mmol), **2** (0.45 mmol), Cu(OAc)_2_ (15 mol %), Phen (15 mol %), Ag_2_O (1.8 equiv), DDQ (40 mol %), K_2_CO_3_ (2.0 equiv), DMF (4 mL), 40 °C, 12 h, Ar. [b] **6**: isolated yield. [c] 24 h.

The substituents of alkynes **2** were then varied in order to explore further the scope of the reaction. As shown in Scheme 3, a series of alkynes **2** with different electron‐withdrawing and electron‐donating substituents on the aromatic ring were subjected to the optimal conditions. The experimental results showed that a broad range of substituents on the arylalkynes **2**, including methyl, methoxy, chloro, bromo, and fluoro groups at the *ortho*‐, *meta*‐, and *para*‐positions of the aromatic ring were well tolerated, providing the desired compounds in moderate to excellent yields (Scheme 3, **6 a**–**6 h**). Furthermore, the structures of compounds **6 a** and **6 g** were unambiguously confirmed via single crystal X‐ray diffraction (see below). An ester group, which may not be tolerated in reactions employing organozinc reagents, is also compatible with this reaction (**6 i**). Importantly, aliphatic alkynes proceeded to give the desired products in moderate to good yields (**6 j** and **6 k**). With a highly electron‐withdrawing CF_3_ substituent, only moderate yields were observed (**6 l** and **6 m**). Unfortunately, less reactive 4‐nitro‐phenyl and 4‐cyano‐phenyl alkynes were not suitable for the reaction under the standard conditions.

**Scheme 4:**
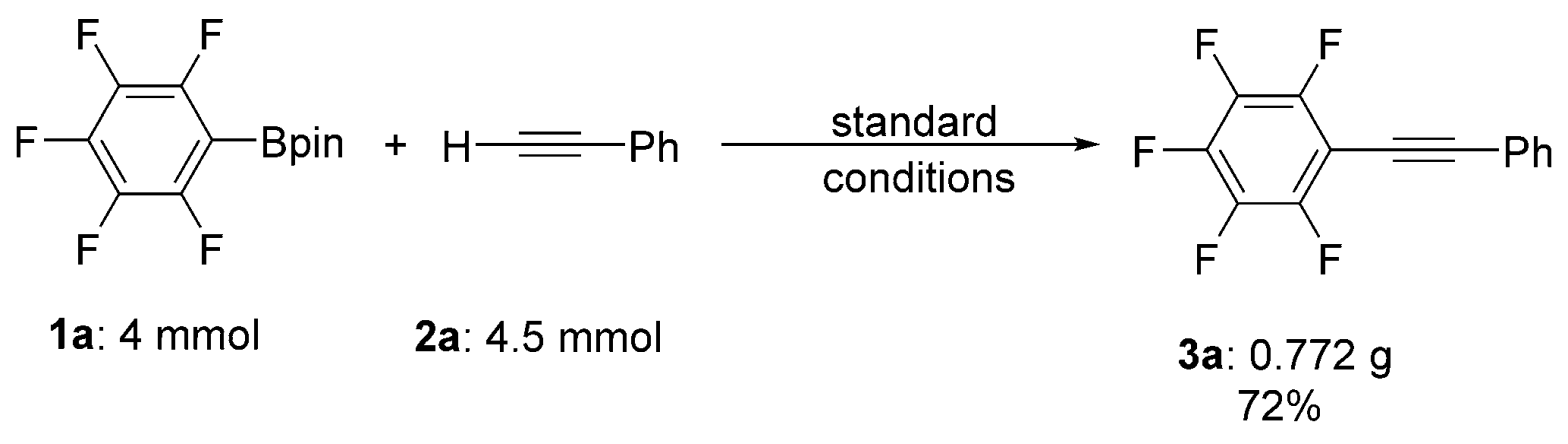
Gram‐scale synthesis of **3a**.

To examine the feasibility of scaling up the reaction, a gram‐scale coupling of C_6_F_5_‐Bpin with phenylacetylene was employed (Scheme 4). The desired coupling product was obtained with minimal loss of yield (72 %).

**Scheme 5:**
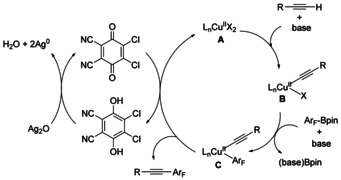
Proposed mechanism.

Based on previous reports,^[22]^ and the aforementioned observations, a plausible catalytic cycle for our oxidative cross‐coupling reaction is shown in Scheme 5. The first step would involve the addition of alkynyl anion leading to the formation of alkynylcopper(II) species **B**. Subsequent transmetalation between Ar_F_Bpin and intermediate **B** occurs to form intermediate **C**. The desired product **3 a** would be generated by C−C reductive elimination. The Cu^0^ species formed is reoxidized by DDQ (see above)[^12^, ^21^] to regenerate **A**, completing the catalytic cycle.

### Molecular and crystal structures: intermolecular π⋅⋅⋅π stacking interactions

**Figure 1:**
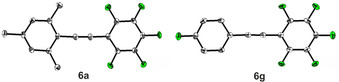
Solid‐state molecular structures of **6 a** and **6 g** determined by single‐crystal X‐ray diffraction at 100 K. Ellipsoids are drawn at the 50 % probability level, and H atoms are omitted for clarity. Colors: white (carbon), green (fluorine).

The crystal structures of the cross‐coupling products **6 a** and **6 g** were analyzed using single‐crystal X‐ray diffraction. The molecular geometries of these compounds in their crystal structures are shown in Figure 1. The central C≡C bond lengths are 1.195(2) and 1.1996(6) Å (Table 2) and, hence, typical of C≡C triple bonds (1.192 Å).^[23]^ The sp‐sp^2^ C−C single bonds between the alkyne and the fully fluorinated phenyl rings are slightly shorter (1.4265(7) and 1.427(2) Å) than the corresponding bonds to the mesityl ring of **6 a** (1.4350(7) Å) or the *para*‐mono‐fluorinated phenyl ring of **6 g** (1.437(2) Å). The sp‐sp^2^ C−C bonds to tetra‐ or pentafluorinated phenyl rings are also shorter than those to the fully or mostly hydrogen‐containing phenyl rings of mixed compounds in other partially fluorinated tolans,^[24]^ rod‐like 1,4‐bis(phenylethynyl)benzenes,^[25]^ and phenyl and perfluorophenyl end‐capped polyynes.^[26]^ The shortening is due to the strong electron‐withdrawing nature of the fluorine atoms, and the length difference is also observed in the co‐crystals of fully hydrogen‐containing and fully fluorinated tolans,^[24]^ rod‐like 1,4‐bis(phenylethynyl)benzenes,^[27]^ and phenyl end‐capped polyynes.^[26]^ The molecules of **6 a** and **6 g** are nearly planar with a very small twist between the aryl moieties (2.959(3) and 3.04(5)°, Table 2). A small twist angle of between 0 and 6° is also typical of the hydrogenated and fluorinated tolans, rod‐like 1,4‐bis(phenylethynyl)benzenes, and phenyl endcapped polyynes.[^24^, ^25^, ^26^, ^27^] Larger twist angles were reported for compounds related to **6 g** in which the fluorine atom at the *para*‐position of the phenyl ring is substituted by iodine (9.4(2)°), bromine (15.69(8)°), and NO_2_ (9.90(7)°).^[28]^ This may be related to the prevalence of different intermolecular interactions in these compounds (see below).

**Table 2:** Selected bond lengths (Å) and angles (°) of **6 a** and **6 g**, and π⋅⋅⋅π stacking distances (Å).

	**6 a**	**6 g**
C≡C triple bond	1.1996(6)	1.195(2)
C_Aryl(H/F)_−C_triple_	1.4350(7)	1.437(2)
C_Aryl(F)_−C_triple_	1.4265(7)	1.427(2)
∢Aryl(F)−Aryl(H/F)	2.959(3)	3.04(5)
centroid‐centroid distance	3.586(3) 3.629(3)	3.705(3) 3.913(3)
interplanar separation	3.361(3)/3.424(3) 3.325(3)/3.376(3)	3.349(2)/3.415(2) 3.379(2)/3.438(2)
offset shift^[a]^	1.248(3)/1.064(2) 1.455(2)/1.332(2)	1.586(3)/1.439(3) 1.975(3)/1.868(3)

[a] The offset shift, also called inter‐centroid shift, is the distance within a plane of an aryl ring between the centroid of the respective aryl ring and the intersection point with the normal to the plane through the centroid of the other aryl ring.

**Figure 2:**
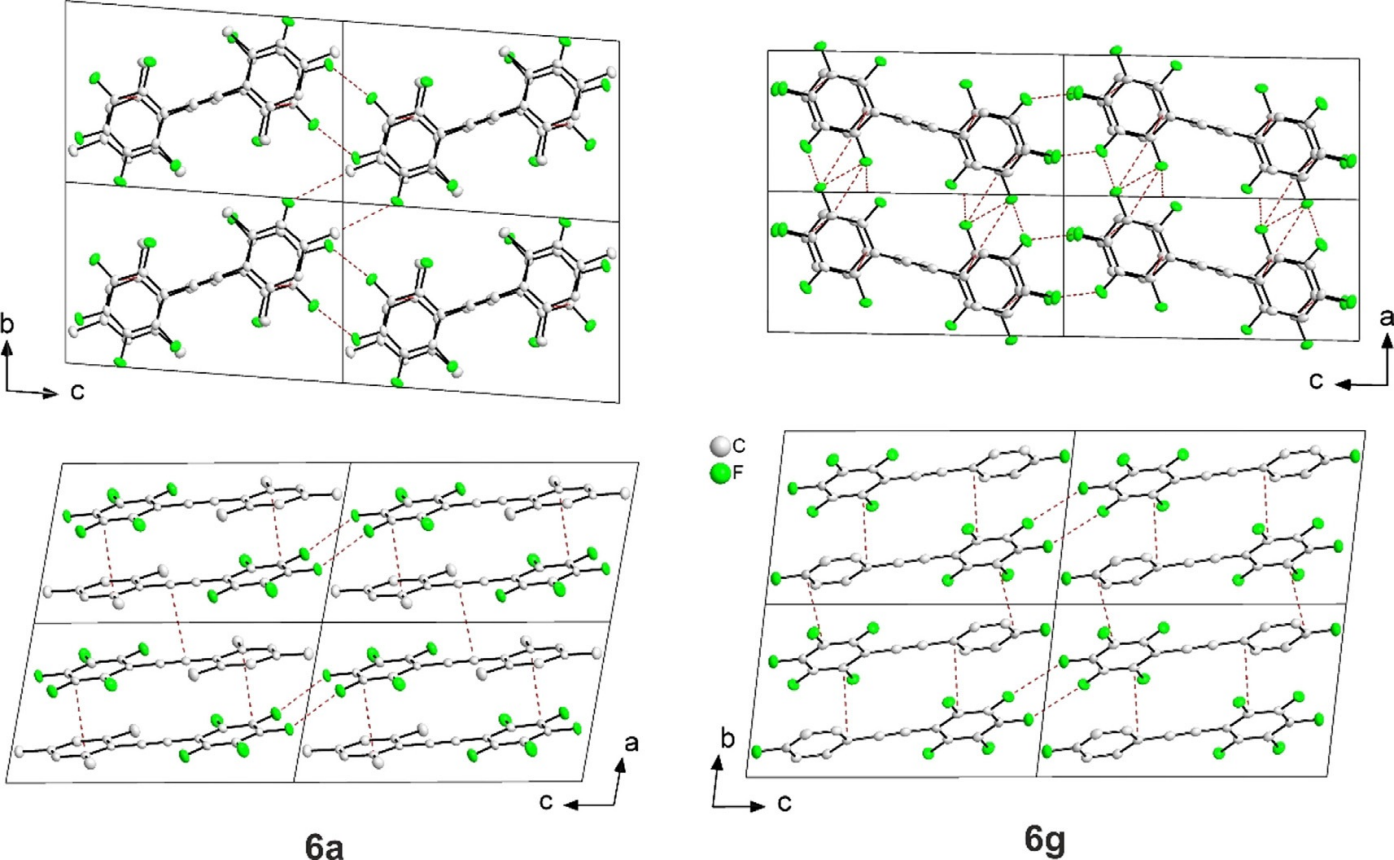
Crystal structures of (left) **6 a** and (right) **6 g** projected along (top) the stacking direction of the molecules, and (bottom) the *b‐* and *a*‐axis, respectively, at 100 K. Molecules are π‐stacked along the *a*‐axis (**6 a**) and the *b*‐axis (**6 g**), respectively, in alternating orientations. Four unit cells are shown in each projection. All ellipsoids are drawn at the 50 % probability level, and H atoms are omitted for clarity. Colors: white (carbon), green (fluorine). Red dotted lines represent intermolecular contacts which are shorter than the sum of the van der Waals radii.

In compounds **6 a** and **6 g**, the nearly planar molecules are related by inversion symmetry and are oriented offset face‐to‐face in a head‐to‐tail fashion forming infinite π‐stacks (Figure 2). The interplanar separations between the aromatic rings (3.325(3) −3.438(2) Å, Table 2) are in the normal range of π–π stacking interactions, which are typical of molecules for which the packing is dominated by arene‐perfluoroarene interactions. The differences in electronegativity of hydrogen and fluorine atoms with respect to the carbon atoms lead to the formation of opposite multipoles for fully fluorinated and nonfluorinated aryl groups and, hence, to attractive multipole forces between these groups.^[29]^ Head‐to‐tail stacking via arene‐perfluoroarene interactions, analogous to that observed in **6 a** and **6 g**, is commonly found in self‐complementary compounds that contain both fluorinated and nonfluorinated aryl groups. Examples are partially fluorinated tolans^[24]^ and phenyl‐endcapped polyynes,^[26]^ but also co‐crystals of bis(phenylethynyl)benzenes with inversely alternating fluorinated and nonfluorinated phenyl rings.^[25]^ We conclude that methylation at the 2‐, 4‐, and 6‐positions of the phenyl ring in **6 a** does not alter this common stacking motif and, hence, the influence of arene‐perfluoroarene interaction on the molecular packing. Arene‐perfluoroarene π‐stacking was also observed in the 1:1 co‐crystal of mesitylene and hexafluorobenzene.^[30]^ Weak intermolecular C−H⋅⋅⋅F, C⋅⋅⋅F, and F⋅⋅⋅F interactions exist between adjacent stacks in **6 a** and **6 g** (Figure 2, Table S2 in the Supporting Information). Mono‐fluorination at the *para*‐position of the phenyl ring in **6 g** does not have a significant influence on the arene‐perfluoroarene packing, which is very similar to that of 1‐pentafluorophenyl‐2‐phenylacetylene.^[24]^ This was expected as the mono‐chlorination of partially fluorinated tolan at the same *para* position did not alter the packing motif.^[28a]^ The effect of halogenation with chlorine, bromine, and iodine atoms at the *para*‐positions of partially fluorinated tolans on the presence of arene‐perfluoroarene interaction, studied earlier by Marder and co‐workers,^[28a]^ revealed the absence of arene‐perfluoroarene stacking only for the compounds substituted with the heavier halogens (Br, I). This was explained by the prevalence of Br⋅⋅⋅Br and I⋅⋅⋅I interactions determining the packing of the molecules.^[28a]^ Also note the larger twist angle between the phenyl rings in these compounds (15.69(8) and 9.4(2)°) when compared to those in arene‐perfluoroarene π‐stacked tolans (see discussion above). Similarly, the substitution of other strong electron‐withdrawing groups such as NO_2_ and CN at the *para*‐position of the phenyl ring in partially fluorinated tolans showed the prevalence of O⋅⋅⋅O and C−H⋅⋅⋅N interactions and the absence of arene‐perfluoroarene interactions in their crystal structures.^[28b]^

## Conclusion

In conclusion, we have developed a copper‐catalyzed method for the direct alkynylation of electron‐deficient polyfluorophenylboronate esters with terminal alkynes. This reaction features broad functional group tolerance, mild reaction conditions, and simple operation. From a synthetic point of view, the present reaction has the potential to be applied widely in organic synthesis because many shelf‐stable aryl and alkyl boronate esters are commercially available. The partially fluorinated tolans also display interesting fluoroarene‐arene π‐stacking interactions in the solid‐state as demonstrated by single‐crystal X‐ray diffraction in two cases.

## Crystallographic details

Crystal data collection and processing parameters are given in the Supporting Information. Deposition Numbers 2000968 (**6 a**), and 2000970 (**6 g**) contain the supplementary crystallographic data for this paper. These data are provided free of charge by the joint Cambridge Crystallographic Data Centre and Fachinformationszentrum Karlsruhe Access Structures service www.ccdc.cam.ac.uk/structures.

## Conflict of interest

The authors declare no conflict of interest.

## Supporting information

As a service to our authors and readers, this journal provides supporting information supplied by the authors. Such materials are peer reviewed and may be re‐organized for online delivery, but are not copy‐edited or typeset. Technical support issues arising from supporting information (other than missing files) should be addressed to the authors.

Supplementary
