# Supplementary material for: Copper‐Catalyzed Oxidative Cross‐Coupling of Electron‐Deficient Polyfluorophenylboronate Esters with Terminal Alkynes
Source: Chemistry. 2020 Nov 9;26(71):17267–74. doi: 10.1002/chem.202002888 (PMC7821263; doi:10.1002/chem.202002888)
Supplement: Supplementary file 1 — Supplementary [file CHEM-26-17267-s001.pdf]

Chemistry–A European Journal

Supporting Information

Copper-Catalyzed Oxidative Cross-Coupling of Electron-Deficient Polyfluorophenylboronate Esters with Terminal Alkynes

Zhiqiang Liu+,[a] Yudha P. Budiman+,[a, c] Ya-Ming Tian,[a] Alexandra Friedrich,[a] Mingming Huang,[a] Stephen A. Westcott,[b] Udo Radius,*[a] and Todd B. Marder*[a]

Table of contents

1. General Information..S3

2. Borylation of Polyfluoroarenes..S3

3. General Procedure..S4

4. Characterization Data..S4

5. ^{1}H NMR, ^{19}F NMR, and ^{13}C{^{1}H} NMR Spectra of All Products S13

6. Crystallographic Details...S46

7. References...S50

1. General Information

NMR spectra were recorded on a Bruker AC–500 spectrometer (500 MHz for ^{1}H NMR, 125 MHz for ^{13}C{^{1}H} NMR, and 470 MHz for ^{19}F NMR) with $CDCl_3$ as the solvent. Chemical shifts (δ) are given in ppm and ^{1}H NMR spectra were referenced via residual proton resonances of $CDCl_3$ (7.26 ppm), ^{13}C{^{1}H} spectra were referenced to $CDCl_3$ (77.16 ppm) and ^{19}F spectra are referenced to external $CFCl_3$. The following abbreviations are used to indicate multiplicities: s = singlet; d = doublet; t = triplet; q = quartet; m = multiplet. GCMS analyses were performed on an Agilent Technologies GCMS system (GC 7890A, EI-MS 5975C). HRMS were recorded using a Thermo Scientific Exactive Plus Orbitrap MS system with either an HESI source with an aux-gas temperature of 50 °C or an APCI source with a corona needle with an aux-gas temperature of 400 °C. Chemical yields refer to pure, isolated products. Automated flash chromatography was performed on silica gel (Biotage SNAP catridge KP-Sil), obtained from Biotage, using a Biotage® Isolera Four Flash system. Solvents were generally removed using a rotary evaporator *in vacuo* at a maximum temperature of 55 °C. Unless otherwise stated, all reagents were purchased from commercial sources and used without further purification. B_2pin_2 was kindly provided by AllyChem Co. Ltd. (Dalian, China). Infrared spectra were recorded on a Nicolet 380 FT-IR spectrometer as solids, using an ATR unit, and are reported in cm^{-1}. Elemental analyses were performed on a LECO CHNS-932 Elemental Analyzer in our institute.

2. Borylation of Polyfluoroarenes

In an argon filled glovebox, a solution of $[Ir(COD)(OMe)]_2$ (0.5 mol%), 4,4’-di-tert-butyl-2,2’-bipyridine (2 mol%), bis(pinacolato)diboron (B_2pin_2) (0.5 eq) and polyfluoroarene (1 eq) in hexane (dry and degassed) was stirred at room temperature in a sealed reaction vessel for 48 h.[1] The volatile materials were removed *in vacuo* to give the crude product, together with unreacted starting arene. The residue was then purified by flash chromatography on silica gel to provide the corresponding fluoroarylboronate ester product.

3. General Procedure

In an argon filled glovebox, a microwave reaction tube with a sealable crimp-cap and equipped with a magnetic stir bar was charged with a polyfluorophenylboronate ester **1** (0.4 mmol), terminal alkynes **2** (0.45 mmol), $Cu(OAc)_2$ (11 mg, 15 mol %), Phen (11 mg, 15 mol %), DDQ (50 mg, 40 mol %), K_2CO_3 (138 mg, 0.8 mmol), in DMF (3.0 mL, dried and degassed) and Ag_2O (1.8/1.2 eq, 167 mg/111 mg) was added at room temperature. The sealed reaction vessel was removed from the glovebox and placed in an oil bath at 40 °C for 12 h. After the reaction was completed, it was cooled to room temperature and monitored for completion by TLC. The resulting solution was poured into saturated brine (5 mL) and then extracted with EtOAc (two times). The combined organic layers were dried over anhydrous Na_2SO_4 and the solvents were removed *in vacuo*. The residue was purified by flash chromatography on silica gel (eluant: n-pentane) to give the desired product.

4. Characterization Data

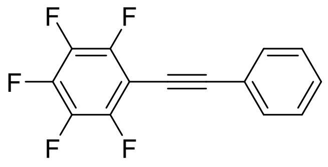

(**3a**):[2] 87.9 mg, 82% yield, white solid. ^{1}H NMR (500 MHz, $CDCl_3$): δ (ppm) = 7.59-7.57 (dm, J = 7 Hz, 2H), 7.43-7.37 (m, 3H). ^{13}C{^{1}H} NMR (125 MHz, $CDCl_3$): δ (ppm) = 147.1 (dm, J_{F-C} = 250 Hz), 141.4 (dm, J_{F-C} = 250 Hz), 137.6 (dm, J_{F-C} = 250 Hz), 131.9, 129.6, 128.5, 121.6, 101.5 (m), 100.3 (tm, J_{F-C} = 18 Hz), 73.1 (m). ^{19}F NMR (470 MHz, $CDCl_3$): δ (ppm) = -136.1- -136.2 (m, 2F), -152.8 - -152.9 (m, 1F), -161.9 - -162.0 (m, 2F). HRMS (ESI): calcd. for $C_{14}H_5F_5$: 268.0306, found: 268.0295.

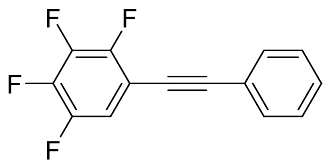

(**3b**): 92 mg, 92% yield, white solid. IR (ATR[cm^{-1}]) 2223 ($\nu_{C\equiv C}$). ^{1}H NMR (500 MHz, $CDCl_3$): δ (ppm) = 7.56-7.54 (m, 2H), 7.41-7.36 (m, 3H), 7.16-7.10 (m, 1H). ^{13}C{^{1}H} NMR

(125 MHz, $CDCl_3$): δ (ppm) = 148.2 (dm, J_{F-C} = 250 Hz), 146.9 (dm, J_{F-C} = 250 Hz), 141.9 (dm, J_{F-C} = 248 Hz), 141.8 (dm, J_{F-C} = 250 Hz), 131.8, 129.3, 128.5, 121.8, 114.2 (dd, J_{F-C} = 21 Hz, 4 Hz), 108.5 (m), 96.5 (m), 79.6 (m). ^{19}F NMR (470 MHz, $CDCl_3$): δ (ppm) = -135.2- -135.3 (m, 1F), -139.3 - -139.4 (m, 1F), -153.7 - -153.8 (m, 1F), -154.9 - -155.1 (m, 1F). HRMS (ESI): calcd. for $C_{14}H_6F_4$: 250.0400, found: 250.0396. Elemental analysis calcd for $C_{14}H_6F_4$: C 67.21, H 2.24; found: C 67.47, H 2.49.

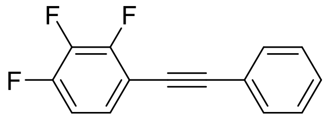

(**3c**):[3] 77 mg, 83% yield, white solid. ^{1}H NMR (500 MHz, $CDCl_3$): δ (ppm) = 7.57-7.55 (m, 2H), 7.38-7.37 (m, 3H), 7.27-7.22 (m, 1H), 6.98-6.93 (m, 1H). $^{13}C\{^1H\}$ NMR (125 MHz, $CDCl_3$): δ (ppm) = 151.9 (ddd, J_{F-C} = 250 Hz, 12 Hz, 4 Hz), 151.3 (ddd, J_{F-C} = 250 Hz, 10 Hz, 3 Hz), 140.2 (dt, J_{F-C} = 250 Hz, 15 Hz), 131.7, 129.0, 128.5, 127.0 (ddd, J_{F-C} = 8 Hz, 4 Hz, 1 Hz), 122.3, 112.3 (dd, J_{F-C} = 19 Hz, 4 Hz), 109.8 (dd, J_{F-C} = 13 Hz, 4 Hz), 95.3 (m), 80.5 (m). ^{19}F NMR (470 MHz, $CDCl_3$): δ (ppm) = -130.2- -130.3 (m, 1F), -131.9 - -132.0 (m, 1F), -159.5 - -159.6 (m, 1F). HRMS (ESI): calcd. for $C_{14}H_7F_3$ $[M+H]^+$ 233.0573, found: 233.0562.

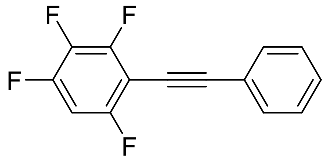

(**3d**): 70 mg, 70% yield, white solid. IR (ATR[cm^{-1}]) 2225 ($\nu_{C\equiv C}$). ^{1}H NMR (500 MHz, $CDCl_3$): δ (ppm) = 7.59-7.57 (m, 2H), 7.40-7.36 (m, 3H), 6.85-6.80 (m, 1H). $^{13}C\{^1H\}$ NMR (125 MHz, $CDCl_3$): δ (ppm) = 157.4 (dm, J_{F-C} = 250 Hz), 151.7 (dm, J_{F-C} = 248 Hz), 150.6 (dm, J_{F-C} = 250 Hz), 137.3 (dm, J_{F-C} = 249 Hz), 131.8, 129.3, 128.5, 121.9, 100.9 (td, J_{F-C} = 25 Hz, 4 Hz), 100.2 (m), 74.2 (t, J_{F-C} = 3 Hz). ^{19}F NMR (470 MHz, $CDCl_3$): δ (ppm) = -111.0 (ddd, J = 11 Hz, 9 Hz, 2 Hz , 1F), -128.5 (ddd, J = 21 Hz, 6 Hz, 2 Hz, 1F), -130.1 (dddd, J = 21 Hz, 10 Hz, 6 Hz, 2 Hz, 1F), -164.4 (tdd, J = 21 Hz, 11 Hz, 6 Hz, 1F). HRMS (ESI): calcd. for $C_{14}H_6F_4$: 250.0400, found: 250.0396. Elemental analysis calcd for $C_{14}H_6F_4$: C 67.21, H 2.24; found: C 67.55, H 2.41.

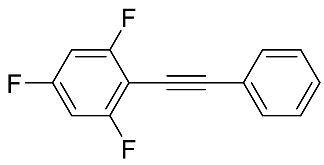

(**3e**): 66.8 mg, 72% yield, white solid. IR (ATR[cm^{-1}]) 2223 ($\nu_{C\equiv C}$). ^{1}H NMR (500 MHz, $CDCl_3$): δ (ppm) = 7.58-7.56 (m, 2H), 7.38-7.32 (m, 3H), 6.74-6.71 (m, 2H). $^{13}C\{^1H\}$ NMR (125 MHz, $CDCl_3$): δ (ppm) = 163.2 (dm, $J_{F\text{-}C}$ = 250 Hz), 162.2 (dm, $J_{F\text{-}C}$ = 250 Hz), 131.7, 128.9, 128.4, 122.4, 100.5 (m), 98.8, 75.1. ^{19}F NMR (470 MHz, $CDCl_3$): δ (ppm) = -104.3 - -104.4 (m, 2F), -105.2 - -105.3 (m, 1F). HRMS (ESI): calcd. for $C_{14}H_7F_3$: 232.0494, found: 232.0490. Elemental analysis calcd for $C_{14}H_7F_3$: C 72.42, H 3.04; found: C 72.71, H 3.24.

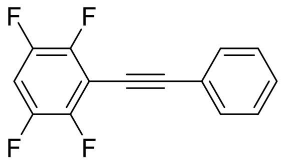

(**3f**): 86 mg, 86% yield, white solid. IR (ATR[cm^{-1}]) 2227 ($\nu_{C\equiv C}$). ^{1}H NMR (500 MHz, $CDCl_3$): δ (ppm) = 7.61-7.59 (m, 2H), 7.42-7.37 (m, 3H), 7.08-7.02 (m, 1H). $^{13}C\{^1H\}$ NMR (125 MHz, $CDCl_3$): δ (ppm) = 146.6 (dm, $J_{F\text{-}C}$ = 250 Hz), 145.8 (dm, $J_{F\text{-}C}$ = 250 Hz), 131.9, 129.6, 128.5, 121.7, 106.1 (t, $J_{F\text{-}C}$ = 23 Hz), 105.5 (m), 101.8 (t, $J_{F\text{-}C}$ = 4 Hz), 74.4 (t, $J_{F\text{-}C}$ = 4 Hz). ^{19}F NMR (470 MHz, $CDCl_3$): δ (ppm) = -136.6 - -136.7 (m, 2F), -138.9 - -139.1 (m, 2F). HRMS (ESI): calcd. for $C_{14}H_6F_4$: 250.0400, found: 250.0396. Elemental analysis calcd for $C_{14}H_6F_4$: C 67.21, H 2.24; found: C 67.45, H 2.48.

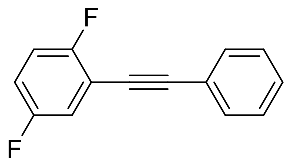

(**3g**): 68.6 mg, 80% yield, oil. ^{1}H NMR (500 MHz, $CDCl_3$): δ (ppm) = 7.57-7.54 (m, 2H), 7.38-7.35 (m, 3H), 7.22-7.19 (m, 1H), 7.08-6.98 (m, 2H). $^{13}C\{^1H\}$ NMR (125 MHz, $CDCl_3$): δ (ppm) = 158.8 (dd, $J_{F\text{-}C}$ = 248 Hz, 3 Hz), 158.1 (dd, $J_{F\text{-}C}$ =250 Hz, 3 Hz), 132.5, 131.8, 129.2, 128.9, 128.5, 122.4, 119.4 (dd, $J_{F\text{-}C}$ = 25 Hz, 2 Hz) , 116.5 (m), 113.1 (m), 95.3 (d, $J_{F\text{-}C}$ = 4 Hz), 81.6 (d, $J_{F\text{-}C}$ = 3 Hz), 73.9. ^{19}F NMR (470 MHz, $CDCl_3$): δ (ppm) = -115.9 - -116.0 (m,

1F), -118.9 - -119.0 (m, 1F). HRMS (ESI): calcd. for $C_{14}H_8F_2$ [M+H] $^+$ 215.0667, found: 215.0662.

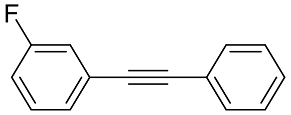

(**3h**):[4] 66.7 mg, 85% yield, oil. ^{1}H NMR (500 MHz, $CDCl_3$): δ (ppm) = 7.56-7.54 (m, 2H), 7.37-7.36 (m, 3H), 7.32-7.30 (m, 2H), 7.25-7.23 (m, 1H), 7.07-7.03 (m, 1H). $^{13}C\{^1H\}$ NMR (125 MHz, $CDCl_3$): δ (ppm) = 162.4 (d, $J_{F\text{-}C}$ = 250 Hz), 131.7, 129.9 (d, $J_{F\text{-}C}$ = 9 Hz), 128.6, 128.4, 127.5 (d, $J_{F\text{-}C}$ = 3 Hz), 125.2 (d, $J_{F\text{-}C}$ = 10 Hz), 122.8, 118.4 (d, $J_{F\text{-}C}$ = 22 Hz), 115.6 (d, $J_{F\text{-}C}$ = 21 Hz), 90.3, 88.1 (d, $J_{F\text{-}C}$ = 4 Hz). ^{19}F NMR (470 MHz, $CDCl_3$): δ (ppm) = -112.9 - -113.0 (m, 1F). HRMS (ESI): calcd. for $C_{14}H_9F$ 196.0688, found: 196.0673.

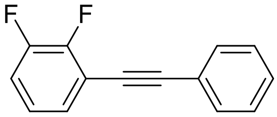

(**3i**): 55.6 mg, 65% yield, oil. ^{1}H NMR (500 MHz, $CDCl_3$): δ (ppm) = 7.58-7.56 (m, 2H), 7.38-7.35 (m, 3H), 7.29-7.26 (m, 1H), 7.17-7.12 (m, 1H), 7.08-7.03 (m, 1H). $^{13}C\{^1H\}$ NMR (125 MHz, $CDCl_3$): δ (ppm) = 150.9 (dd, $J_{F\text{-}C}$ = 249 Hz, 14 Hz), 150.6 (dd, $J_{F\text{-}C}$ = 250 Hz, 12 Hz), 132.5, 131.8, 128.9, 128.4, 128.2 (d, $J_{F\text{-}C}$ = 3 Hz), 123.9 (dd, $J_{F\text{-}C}$ = 12 Hz, 3 Hz), 117.4 (d, $J_{F\text{-}C}$ = 17 Hz), 114.2 (dd, $J_{F\text{-}C}$ = 13 Hz, 2 Hz), 95.6 (d, $J_{F\text{-}C}$ = 4 Hz), 81.6. ^{19}F NMR (470 MHz, $CDCl_3$): δ (ppm) = -134.9 - -135.0 (m, 1F), -137.5 - -137.6 (m, 1F). HRMS (ESI): calcd. for $C_{14}H_8F_2$ [M+H] $^+$ 215.0667, found: 215.0662.

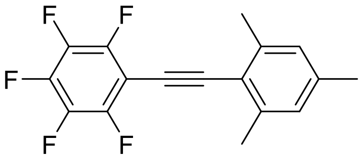

(**6a**): 106.6 mg, 86% yield, white solid. IR (ATR[cm^{-1}]) 2223 ($\nu_{C\equiv C}$). ^{1}H NMR (500 MHz, $CDCl_3$): δ (ppm) = 6.92-6.91 (m, 2H), 2.47 (s, 6H), 2.31 (s, 3H). $^{13}C\{^1H\}$ NMR (125 MHz, $CDCl_3$): δ (ppm) = 146.7 (dm, $J_{F\text{-}C}$ = 250 Hz), 141.1 (dm, $J_{F\text{-}C}$ = 251 Hz), 140.8, 139.5, 137.6 (dm, $J_{F\text{-}C}$ = 252 Hz), 127.8, 118.5, 100.9 (tm, $J_{F\text{-}C}$ = 18 Hz), 100.1 (m), 80.6 (m), 21.4, 20.7. ^{19}F NMR (470 MHz, $CDCl_3$): δ (ppm) = -136.6 - -136.7 (m, 2F), -150.4 (t, J_F = 21 Hz, 1F),

-162.3 - -162.4 (m, 2F). HRMS (ESI): calcd. for $C_{17}H_{11}F_5$ [M+H] $^+$ 311.0854, found: 311.0842. Elemental analysis calcd for $C_{17}H_{11}F_5$: C 65.81, H 3.57; found: C 65.98, H 3.72.

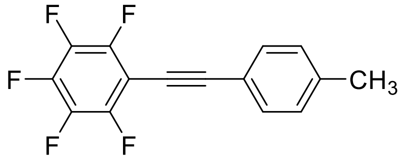

(**6b**):[5] 91.4 mg, 81% yield, white solid. ^{1}H NMR (500 MHz, $CDCl_3$): δ (ppm) = 7.48-7.46 (m, 2H), 7.21-7.19 (m, 2H), 2.39 (s, 3H). $^{13}C\{^1H\}$ NMR (125 MHz, $CDCl_3$): δ (ppm) = 147.1 (dm, $J_{F\text{-}C}$ = 250 Hz), 141.3 (dm, $J_{F\text{-}C}$ = 250 Hz), 140.1, 137.7 (dm, $J_{F\text{-}C}$ = 250 Hz), 131.8, 129.3, 118.5, 101.9 (m), 100.6 (tm, $J_{F\text{-}C}$ = 18 Hz), 72.5 (m), 21.6. ^{19}F NMR (470 MHz, $CDCl_3$): δ (ppm) = -136.2 - -136.3 (m, 2F), -153.3 (t, J_F = 21 Hz, 1F), -162.0 - -162.1 (m, 2F). HRMS (ESI): calcd. for $C_{15}H_7F_5$ [M+H] $^+$ 283.0541, found: 283.0526.

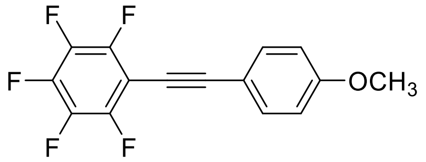

(**6c**):[5] 92.9 mg, 78% yield, white solid. ^{1}H NMR (500 MHz, $CDCl_3$): δ (ppm) = 7.53-7.50 (m, 2H), 6.92-6.89 (m, 2H), 3.85 (s, 3H). $^{13}C\{^1H\}$ NMR (125 MHz, $CDCl_3$): δ (ppm) = 160.7, 147.1 (dm, $J_{F\text{-}C}$ = 252 Hz), 141.1 (dm, $J_{F\text{-}C}$ = 250 Hz), 137.7 (dm, $J_{F\text{-}C}$ = 250 Hz), 133.5, 114.2, 113.6, 101.9 (m), 100.7 (tm, $J_{F\text{-}C}$ = 18 Hz), 72.0 (m), 55.4. ^{19}F NMR (470 MHz, $CDCl_3$): δ (ppm) = -136.5 - -136.6 (m, 2F), -153.7 (t, J_F = 21 Hz, 1F), -162.1 - -162.2 (m, 2F). HRMS (ESI): calcd. for $C_{15}H_7F_5O$ [M+H] $^+$ 299.0490, found: 299.0475.

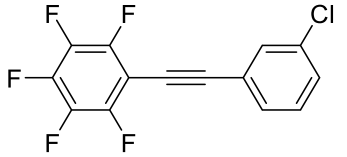

(**6d**):[5] 91.8 mg, 76% yield, white solid. ^{1}H NMR (500 MHz, $CDCl_3$): δ (ppm) = 7.57-7.56 (m, 1H), 7.47-7.45 (td, J = 8 Hz, 2 Hz, 1H), 7.41-7.39 (m, 1H), 7.34-7.31(m, 1H). $^{13}C\{^1H\}$ NMR (125 MHz, $CDCl_3$): δ (ppm) = 147.2 (dm, $J_{F\text{-}C}$ = 250 Hz), 141.7 (dm, $J_{F\text{-}C}$ = 250 Hz), 137.7 (dm, $J_{F\text{-}C}$ = 250 Hz), 134.5, 131.7, 130.0, 129.9, 129.8, 123.2, 99.8 (m), 74.1 (m). ^{19}F NMR (470 MHz, $CDCl_3$): δ (ppm) = -135.6 - -135.7 (m, 2F), -151.8 - -151.9 (m, 1F), -161.5 - -161.6 (m, 2F). HRMS (ESI): calcd. for $C_{14}H_4F_5Cl$ 301.9926, found: 301.9905.

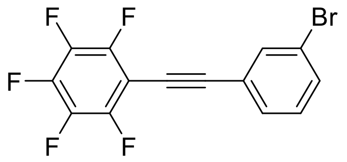

(**6e**): 96.8 mg, 68% yield, white solid. IR (ATR[cm^{-1}]) 2234 ($\nu_{C\equiv C}$). ^{1}H NMR (500 MHz, $CDCl_3$): δ (ppm) = 7.73 (t, *J* = 2 Hz, 1H), 7.56-7.54 (m, 1H), 7.52-7.50 (td, *J* = 8 Hz, 2 Hz, 1H), 7.27 (t, *J* = 8 Hz, 1H). ^{13}C{^{1}H} NMR (125 MHz, $CDCl_3$): δ (ppm) = 147.2 (dm, $J_{F\text{-}C}$ = 249 Hz), 141.7 (dm, $J_{F\text{-}C}$ = 251 Hz), 137.7 (dm, $J_{F\text{-}C}$ = 251 Hz), 134.5, 132.8, 130.5, 129.5, 123.5, 122.4, 99.9 (m), 99.7 (m), 74.2 (m). ^{19}F NMR (470 MHz, $CDCl_3$): δ (ppm) = -135.6 - -135.7 (m, 2F), -151.8 - -151.9 (m, 1F), -161.4 - -161.5 (m, 2F). HRMS (ESI): calcd. for $C_{14}H_4BrF_5$: 345.9411, found: 345.9407. Elemental analysis calcd for $C_{14}H_4BrF_5$: C 48.45, H 1.16; found: C 48.62, H 1.36.

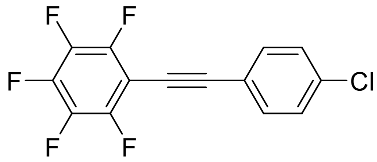

(**6f**): 72.5 mg, 60% yield, white solid. IR (ATR[cm^{-1}]) 2232 ($\nu_{C\equiv C}$). ^{1}H NMR (500 MHz, $CDCl_3$): δ (ppm) = 7.52-7.50 (dm, *J* = 8 Hz, 2H), 7.38-7.36 (dm, *J* = 8 Hz, 2H). ^{13}C{^{1}H} NMR (125 MHz, $CDCl_3$): δ (ppm) = 147.2 (d, $J_{F\text{-}C}$ = 250 Hz), 141.5 (d, $J_{F\text{-}C}$ = 252 Hz), 137.6 (dm, $J_{F\text{-}C}$ = 250 Hz), 135.9, 133.1, 128.9, 120.0, 100.3 (m), 100.0 (m), 74.1 (m). ^{19}F NMR (470 MHz, $CDCl_3$): δ (ppm) = -135.8 - -135.9 (m, 2F), -152.1 - -152.2 (m, 1F), -161.6 - -161.7 (m, 2F). HRMS (ESI): calcd. for $C_{14}H_4ClF_5$: 301.9916, found: 301.9907. Elemental analysis calcd for $C_{14}H_4ClF_5$: C 55.56, H 1.33; found: C 55.72, H 1.49.

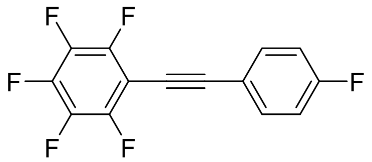

(**6g**):[5] 74.4 mg, 65% yield, white solid. ^{1}H NMR (500 MHz, $CDCl_3$): δ (ppm) = 7.59-7.55 (m, 2H), 7.11-7.07 (m, 2H). ^{13}C{^{1}H} NMR (125 MHz, $CDCl_3$): δ (ppm) = 163.3 (d, $J_{F\text{-}C}$ = 250 Hz), 147.1 (dm, $J_{F\text{-}C}$ = 250 Hz), 141.5 (dm, $J_{F\text{-}C}$ = 252 Hz), 137.7 (dm, $J_{F\text{-}C}$ = 251 Hz), 133.9 (d, $J_{F\text{-}C}$ = 9 Hz), 117.6 (d, $J_{F\text{-}C}$ = 7 Hz), 116.0 (d, $J_{F\text{-}C}$ = 15 Hz), 100.3 (m), 72.9 (m). ^{19}F NMR (470 MHz, $CDCl_3$): δ (ppm) = -108.3 - -108.4 (m, 1F), -136.0 - -136.1 (m, 2F), -152.6 (t, J_F =

21 Hz, 1F), -161.7 - -161.8 (m, 2F). HRMS (ESI): calcd. for $C_{14}H_4F_6$ [M+H] $^+$ 287.0290, found: 287.0273.

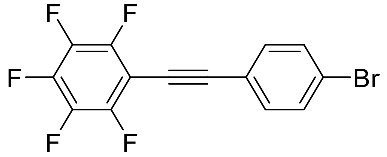

(**6h**):[5] 117.6 mg, 85% yield, white solid. ^{1}H NMR (500 MHz, $CDCl_3$): δ (ppm) = 7.55-7.52 (dm, J = 8 Hz, 2H), 7.45-7.43 (dm, J = 9 Hz, 2H). ^{13}C{^{1}H} NMR (125 MHz, $CDCl_3$): δ (ppm) = 147.1 (dm, $J_{F\text{-}C}$ = 250 Hz), 141.6 (dm, $J_{F\text{-}C}$ = 251 Hz), 137.7 (dm, $J_{F\text{-}C}$ = 250 Hz),133.3, 131.9, 124.2, 120.5,100.3 (m), 100.0 (tm, $J_{F\text{-}C}$ = 17 Hz), 74.2 (m). ^{19}F NMR (470 MHz, $CDCl_3$): δ (ppm) = -135.8 - -135.9 (m, 2F), -152.1 - -152.2 (m, 1F), -161.6 - -161.7 (m, 2F). HRMS (ESI): calcd. for $C_{14}H_4F_5Br$ 345.9411, found: 345.9403.

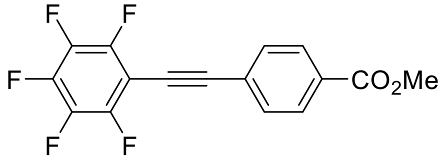

(**6i**):[2] 100.4 mg, 77% yield, white solid. ^{1}H NMR (500 MHz, $CDCl_3$): δ (ppm) = 8.07-8.05 (m, 2H), 7.65-7.63 (m, 2H), 3.94 (s, 3H). ^{13}C{^{1}H} NMR (125 MHz, $CDCl_3$): δ (ppm) = 166.3, 147.2 (dm, $J_{F\text{-}C}$ = 253 Hz), 141.7 (d, $J_{F\text{-}C}$ = 250 Hz), 137.7 (dm, $J_{F\text{-}C}$ = 250 Hz), 131.8, 130.8, 129.6, 126.0, 100.4 (m), 99.8, 75.6, 52.4. ^{19}F NMR (470 MHz, $CDCl_3$): δ (ppm) = -135.5 - -135.6 (m, 2F), -151.6 - -151.7 (m, 1F), -161.4 - -161.5 (m, 2F). HRMS (ESI): calcd. for $C_{16}H_7F_5O_2$ 326.0365, found: 326.0372.

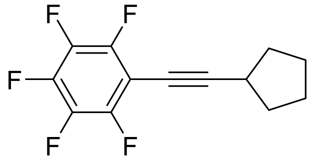

(**6j**): 91.8 mg, 76% yield, oil. ^{1}H NMR (500 MHz, $CDCl_3$): δ (ppm) = 2.94-2.88 (m, 1H), 2.04-1.99 (m, 2H), 1.81-1.73 (m, 4H), 1.65-1.61(m, 2H). ^{13}C{^{1}H} NMR (125 MHz, $CDCl_3$): δ (ppm) = 147.3 (dm, $J_{F\text{-}C}$ = 251 Hz), 140.7 (dm, $J_{F\text{-}C}$ = 250 Hz), 137.6 (dm, $J_{F\text{-}C}$ = 248 Hz), 108.3 (m), 100.8 (m), 64.1 (m), 33.6, 30.9, 25.1. ^{19}F NMR (470 MHz, $CDCl_3$): δ (ppm) =

-137.2 - -137.3 (m, 2F), -154.7 (t, J_F = 21 Hz, 1F), -162.5 - -162.6 (m, 2F). HRMS (ESI): calcd. for $C_{13}H_9F_5$ $[M-H]^-$ 259.0541, found: 259.0534.

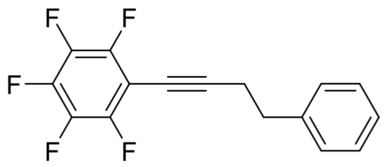

(**6k**): 94.7 mg, 80% yield, oil. ^{1}H NMR (500 MHz, $CDCl_3$): δ (ppm) = 7.34-7.31 (m, 2H), 7.28-7.23 (m, 3H), 2.96 (t, J = 7 Hz, 2H), 2.79 (t, J = 7 Hz, 2H). $^{13}C\{^1H\}$ NMR (125 MHz, $CDCl_3$): δ (ppm) = 147.5 (dm, $J_{F\text{-}C}$ = 250 Hz), 141.0 (dm, $J_{F\text{-}C}$ = 252 Hz), 137.6 (dm, $J_{F\text{-}C}$ = 250 Hz) 139.9, 128.5, 128.4, 126.5, 102.9 (m), 100.4 (tm, $J_{F\text{-}C}$ = 18 Hz), 65.4 (m), 34.5, 22.0. ^{19}F NMR (470 MHz, $CDCl_3$): δ (ppm) = -136.9 - -137.0 (m, 2F), -154.1 (t, J_F = 21 Hz, 1F), -162.3 - -162.4 (m, 2F). HRMS (ESI): calcd. for $C_{16}H_9F_5$ $[M+H]^+$ 297.0697, found: 297.0687

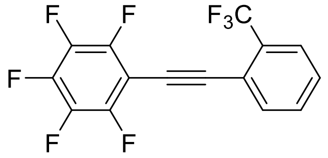

(**6l**): 64.5 mg, 48% yield, white solid. IR (ATR[cm^{-1}]) 2234 ($\nu_{C\equiv C}$). ^{1}H NMR (500 MHz, $CDCl_3$): δ (ppm) = 7.73 (d, J = 8 Hz, 2H), 7.57 (t, J = 8 Hz, 1H), 7.52 (t, J = 8 Hz, 1H). $^{13}C\{^1H\}$ NMR (125 MHz, $CDCl_3$): δ (ppm) = 147.3 (dm, $J_{F\text{-}C}$ = 250 Hz), 141.9 (dm, $J_{F\text{-}C}$ = 249 Hz), 137.7 (dm, $J_{F\text{-}C}$ = 251 Hz), 134.2, 132.0 (q, $J_{F\text{-}C}$ = 31 Hz), 131.6, 129.4, 126.1 (q, $J_{F\text{-}C}$ = 5 Hz), 125.4 (q, $J_{F\text{-}C}$ = 272 Hz), 122.2, 119.6, 97.0 (m), 78.4. ^{19}F NMR (470 MHz, $CDCl_3$): δ (ppm) = -62.3 (s, 3F), -135.3 - -135.4 (m, 2F), -151.5 - -151.6 (m, 1F), -161.5 - -161.6 (m, 2F). HRMS (ESI): calcd. for $C_{15}H_4F_8$: 336.0180, found: 336.0170. Elemental analysis calcd for $C_{15}H_4F_8$: C 53.59, H 1.20; found: C 53.75, H 1.41.

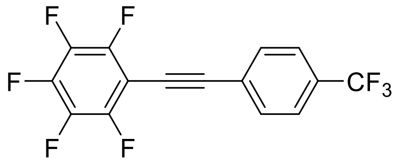

(**6m**):[5] 67.2 mg, 50% yield, white solid. ^{1}H NMR (500 MHz, $CDCl_3$): δ (ppm) = 7.72 (d, J = 8 Hz, 2H), 7.68 (d, J = 8 Hz, 2H) (2nd order AA'BB' spin system; values are approximate).

$^{13}C\{^1H\}$ NMR (125 MHz, $CDCl_3$): δ (ppm) = 147.2 (dm, $^1J_{F-C}$ = 251 Hz), 141.8 (dm, J_{F-C} = 251 Hz), 137.7 (dm, J_{F-C} = 250 Hz), 132.4, 132.2, 131.3 (q, J_{F-C} = 33 Hz), 125.9 (q, J_{F-C} = 273 Hz), 125.5 (q, J_{F-C} = 4 Hz), 122.6, 99.7, 75.3 (m). ^{19}F NMR (470 MHz, $CDCl_3$): δ (ppm) = -63.0 (t, J_F = 1 Hz, 3F), -135.4 - -135.5 (m, 2F), -151.3 - -151.4 (m, 1F), -161.3 - -161.4 (m, 2F). HRMS (ESI): calcd. for $C_{15}H_4F_8$: 336.0180, found: 336.0170.

5. ^{1}H NMR, ^{19}F NMR, and ^{13}C{^{1}H} NMR Spectra of All Products

Compound 3a: ^{1}H NMR spectrum (500 MHz, $CDCl_3$).

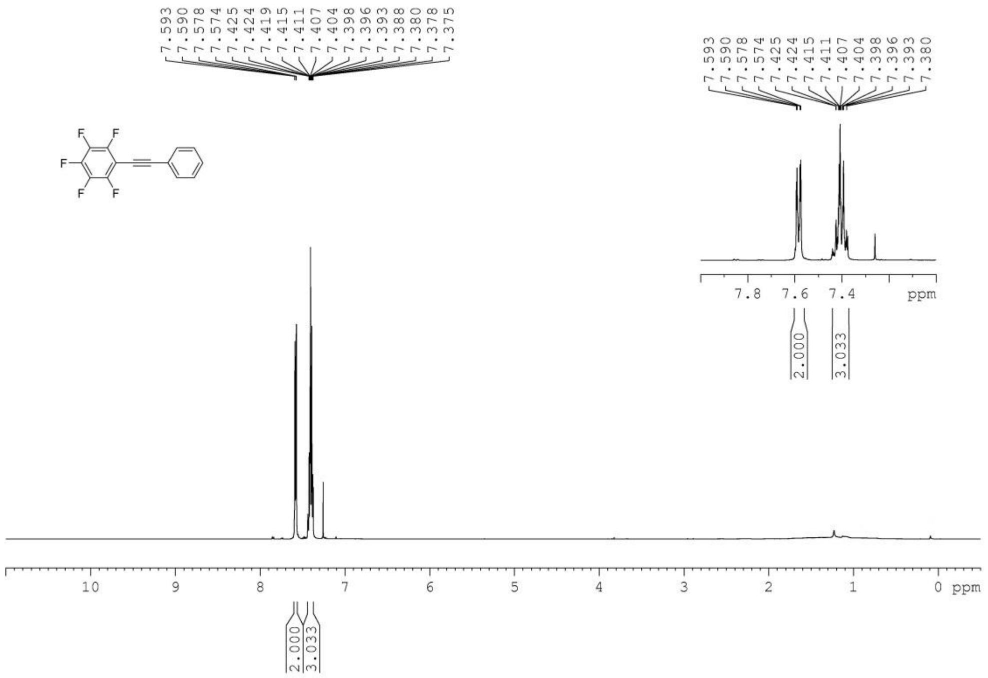

Compound 3a: ^{13}C{^{1}H} NMR spectrum (125 MHz, $CDCl_3$).

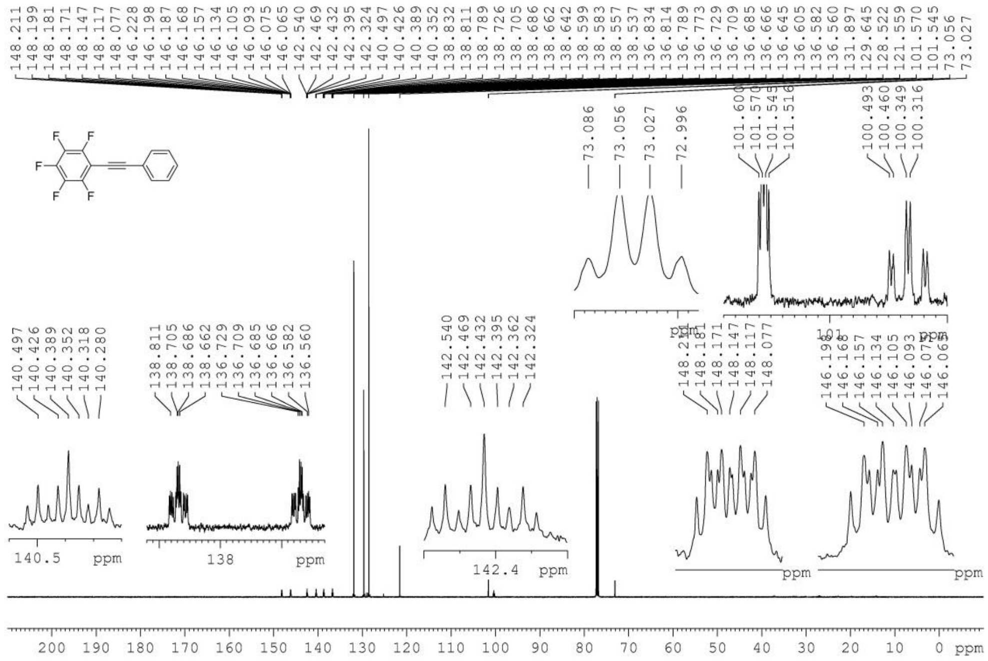

Compound 3a: ^{19}F NMR spectrum (470 MHz, $CDCl_3$).

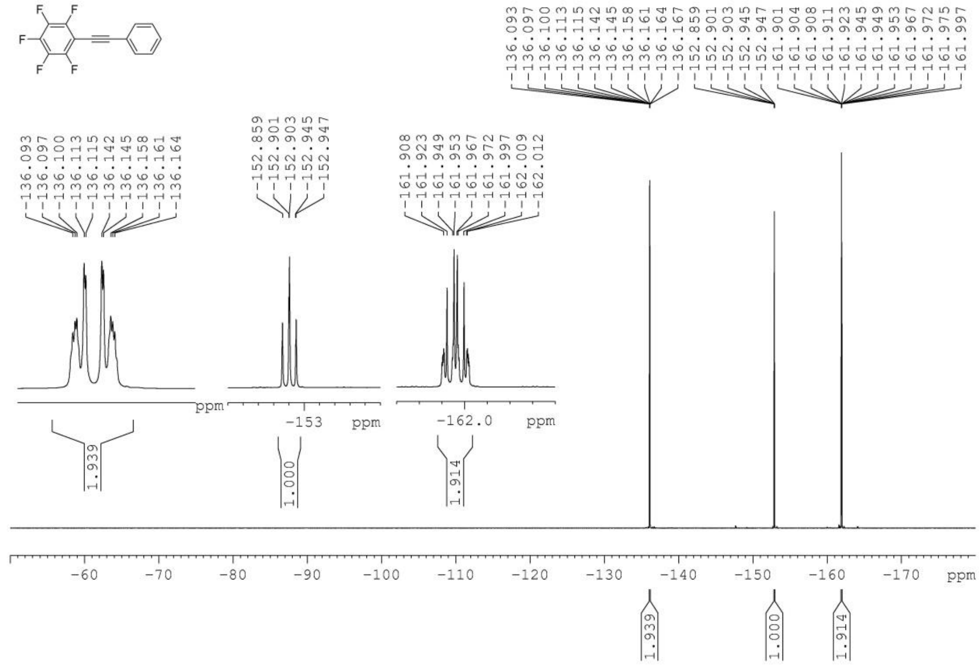

Compound 3b. ^{1}H NMR spectrum (500 MHz, $CDCl_3$).

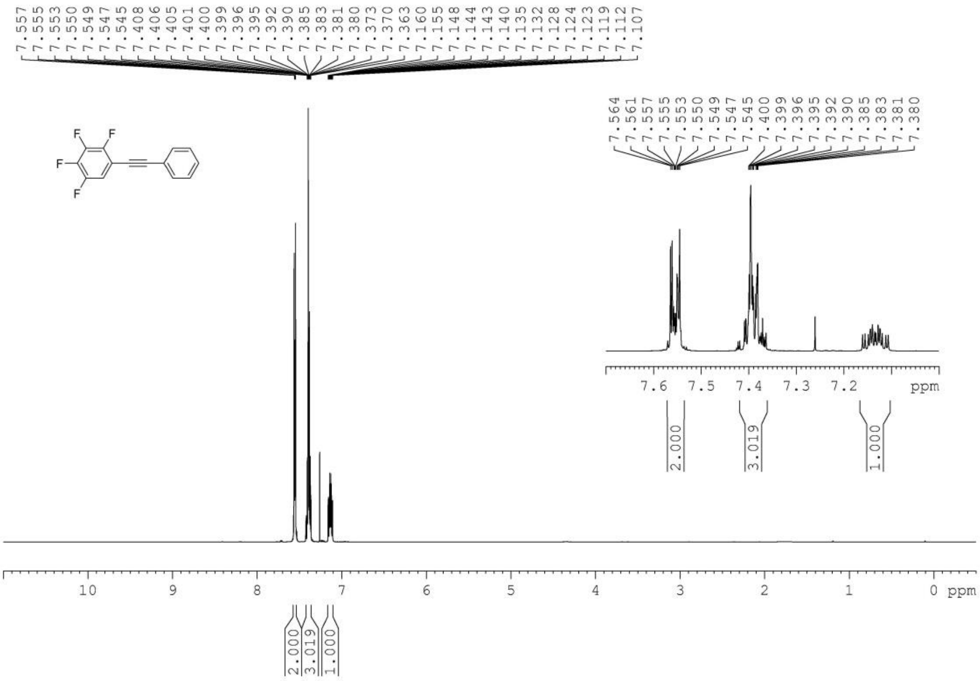

Compound 3b: $^{13}C\{^{1}H\}$ NMR spectrum (125 MHz, $CDCl_3$).

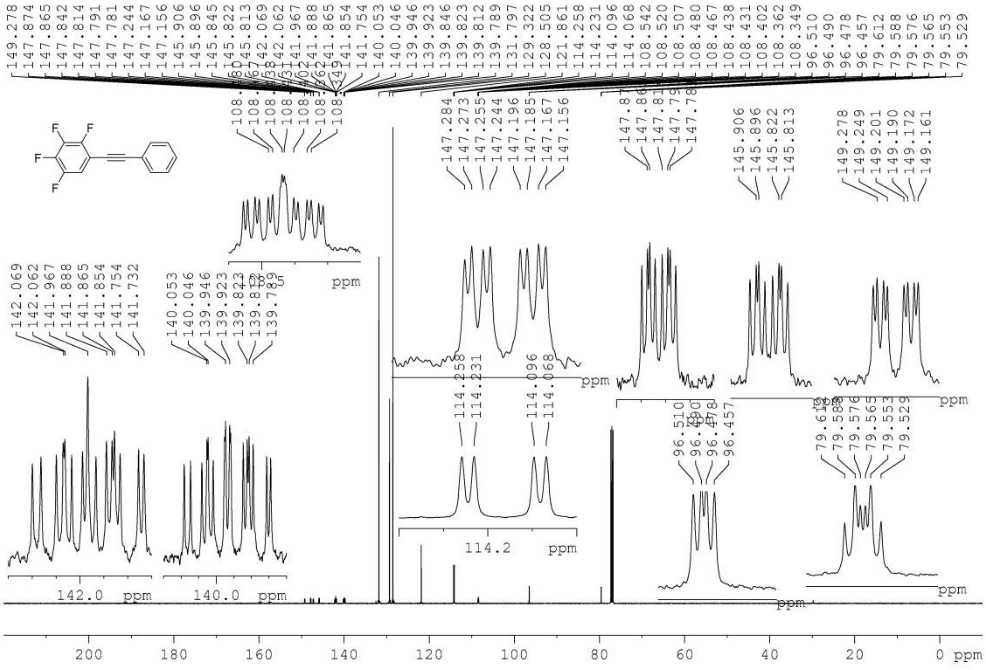

Compound 3b: ^{19}F NMR spectrum (470 MHz, $CDCl_3$).

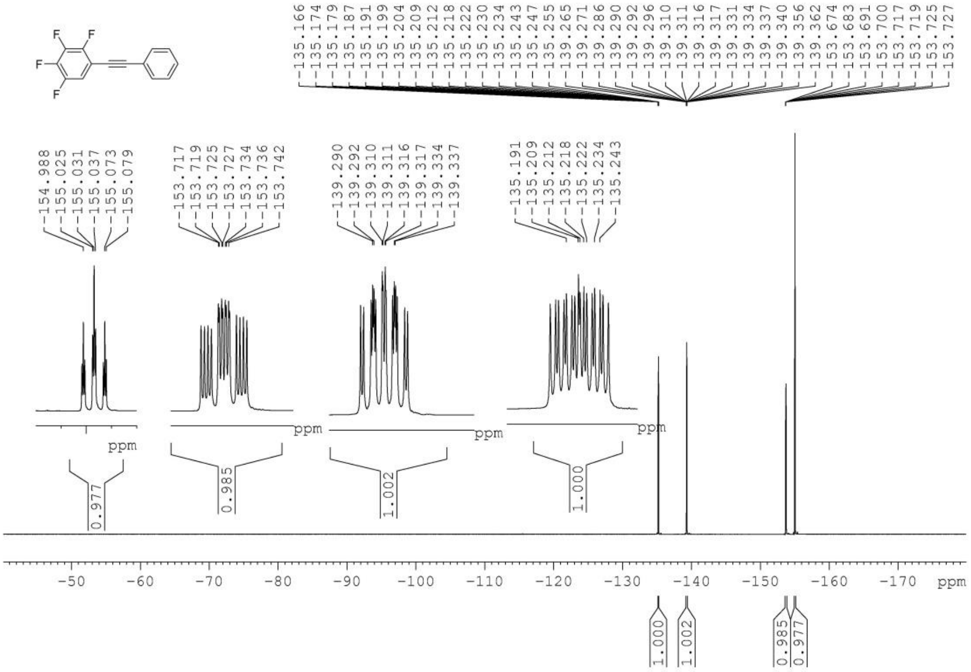

Compound 3c: ^{1}H NMR spectrum (500 MHz, CDCl$_3$).

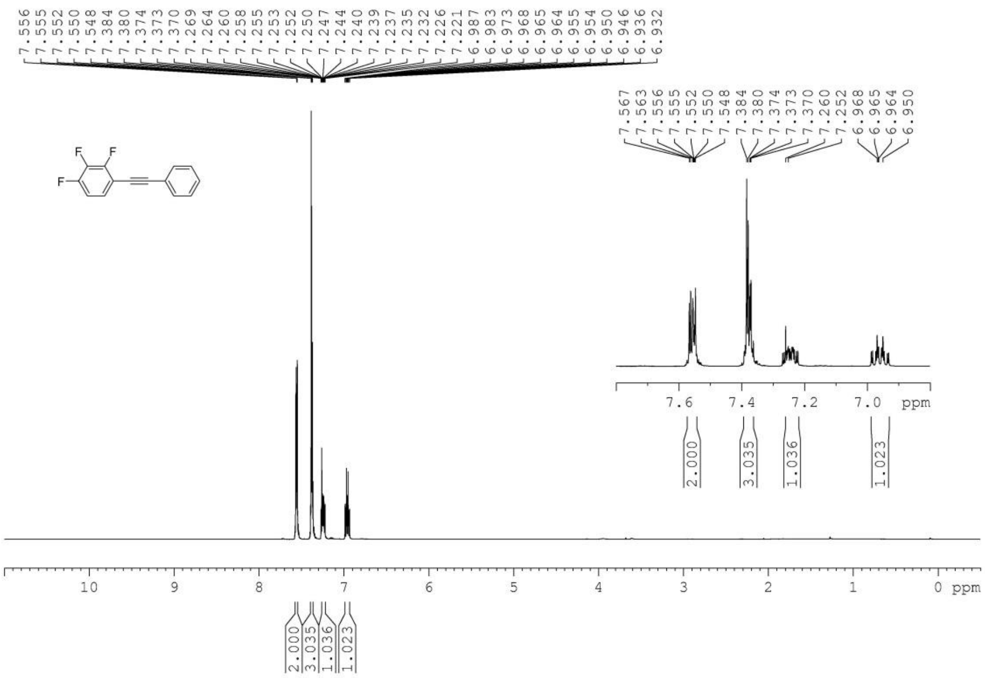

Compound 3c: ^{13}C{^{1}H} NMR spectrum (125 MHz, CDCl$_3$).

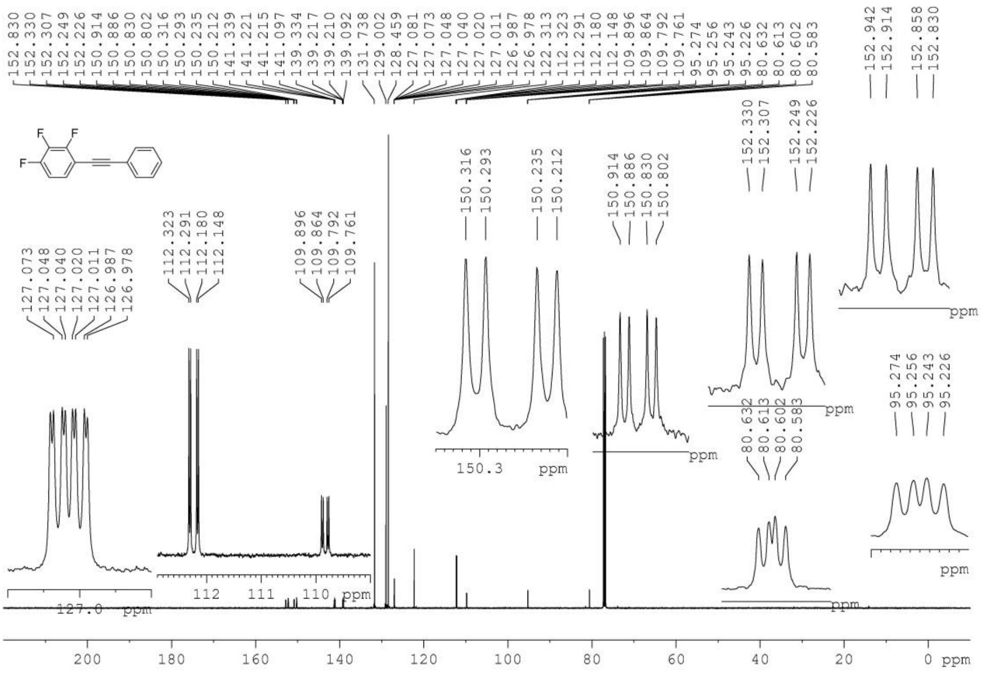

Compound 3c: ^{19}F NMR spectrum (470 MHz, $CDCl_3$).

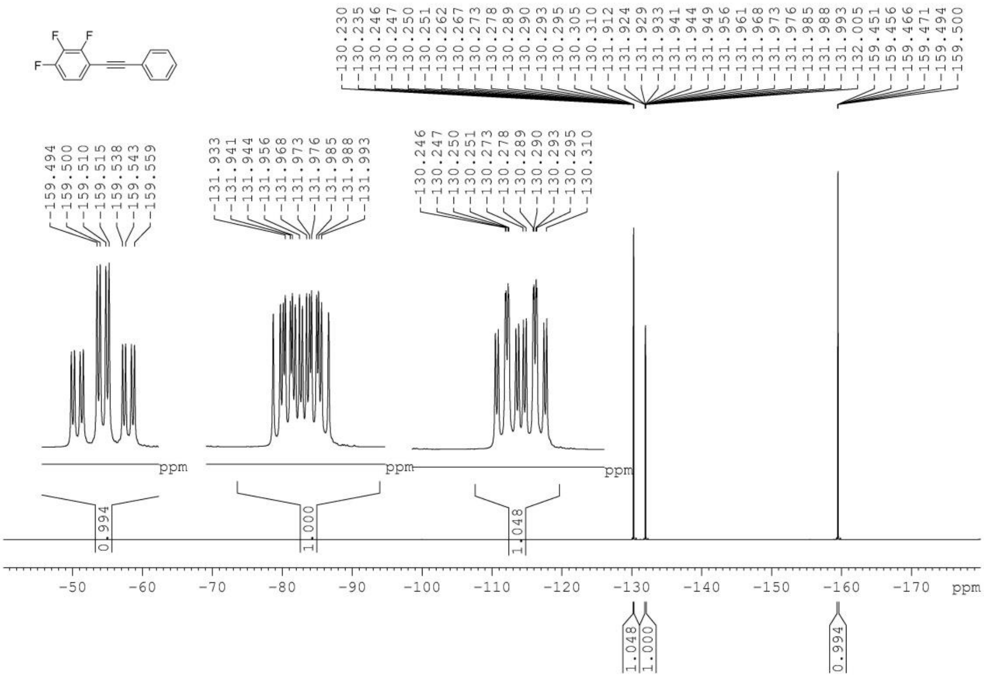

Compound 3d: ^{1}H NMR spectrum (500 MHz, $CDCl_3$).

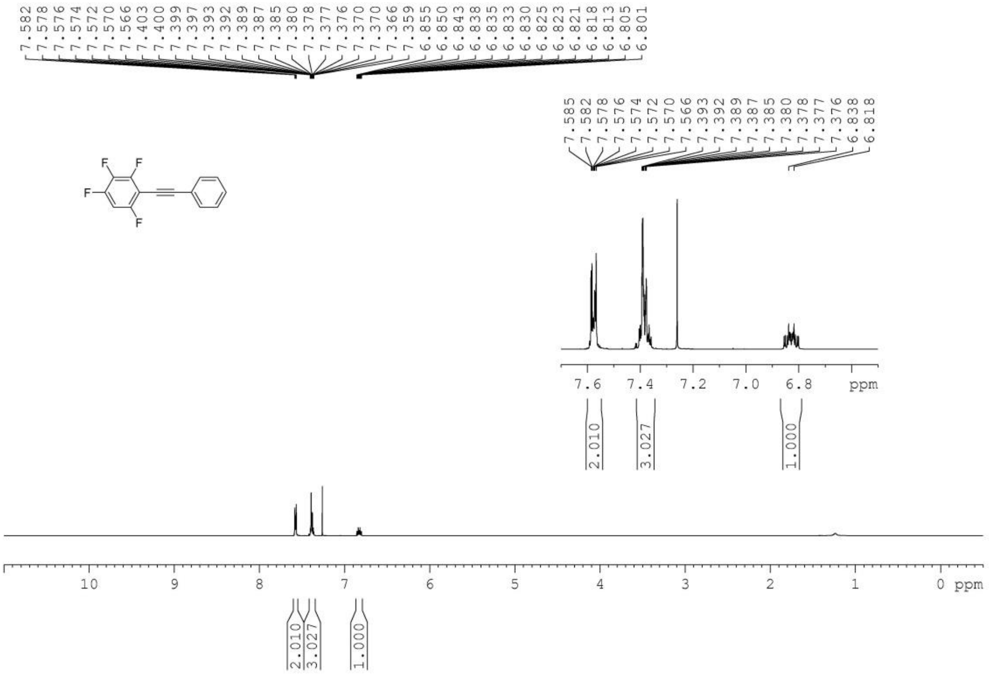

Compound 3d: $^{13}C\{^1H\}$ NMR spectrum (125 MHz, $CDCl_3$).

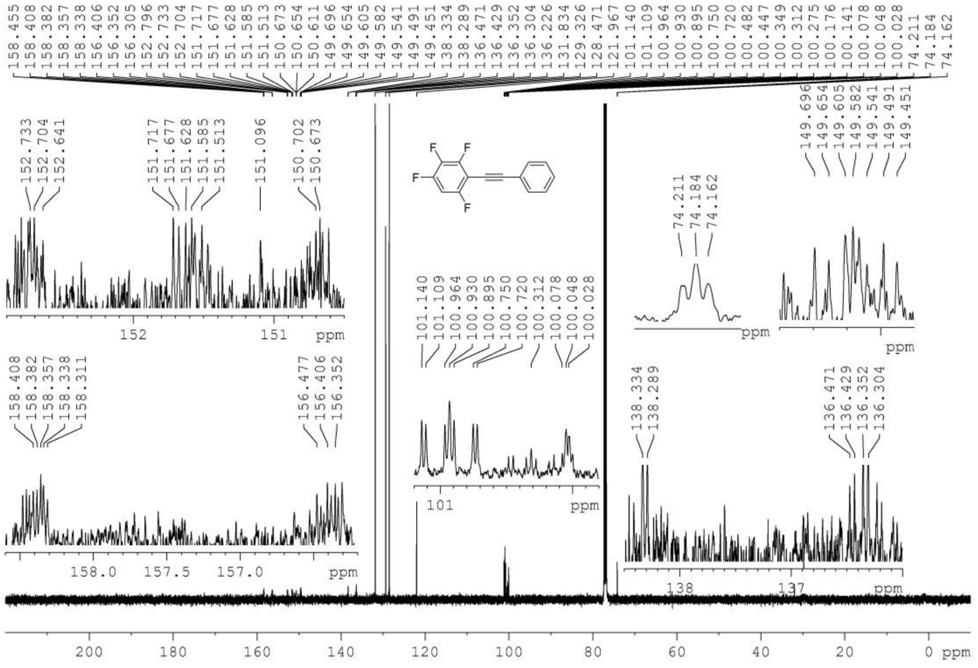

Compound 3d: ^{19}F NMR spectrum (470 MHz, $CDCl_3$).

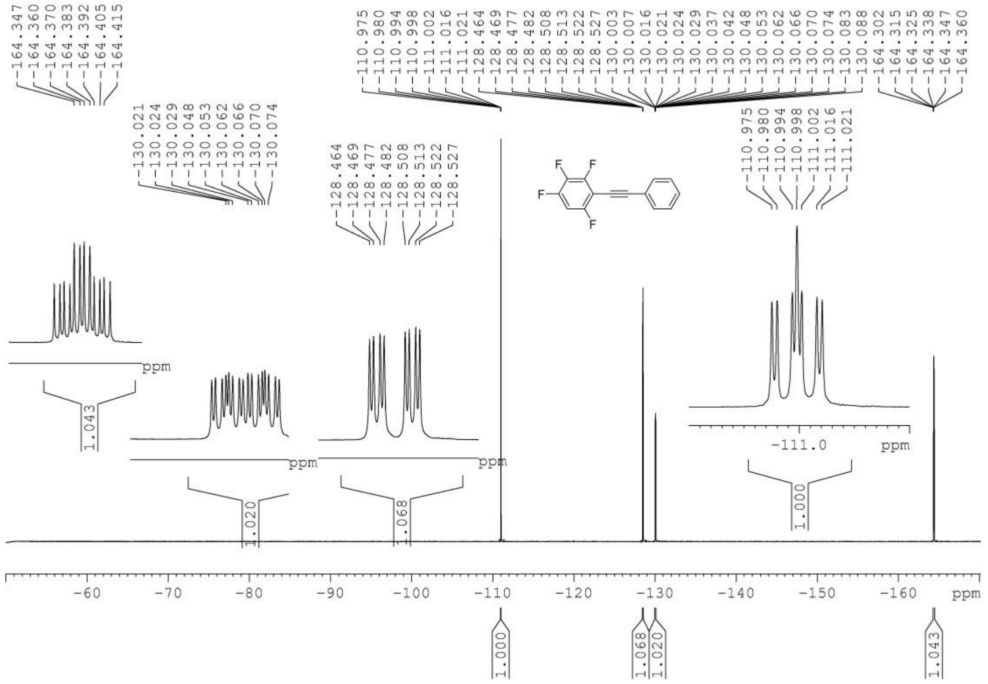

Compound 3e: ^{1}H NMR spectrum (500 MHz, $CDCl_3$).

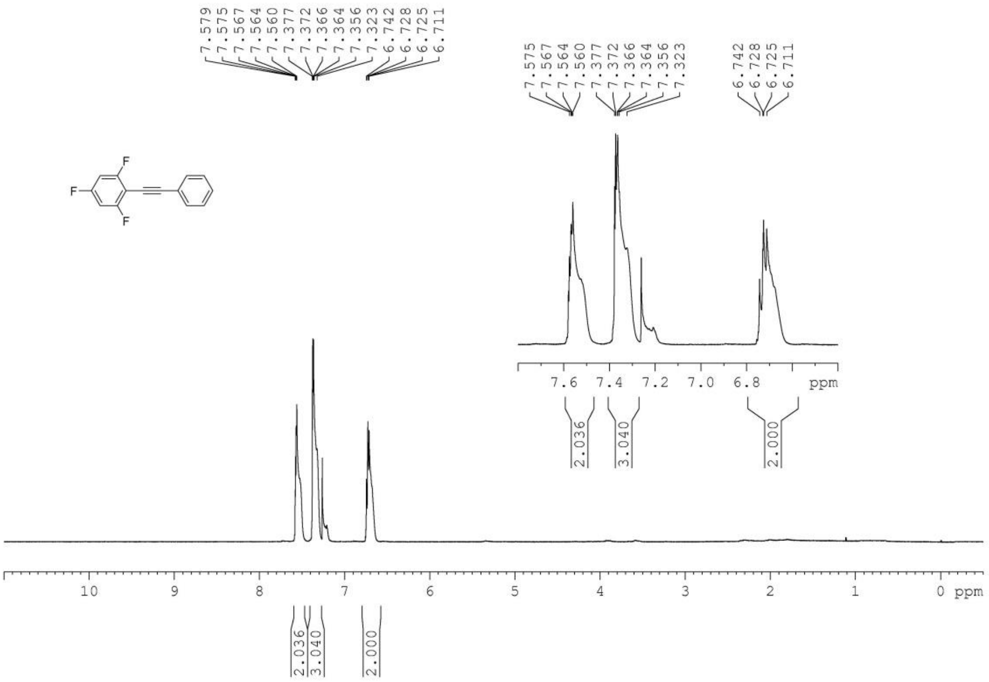

Compound 3e: ^{13}C{^{1}H} NMR spectrum (125 MHz, $CDCl_3$).

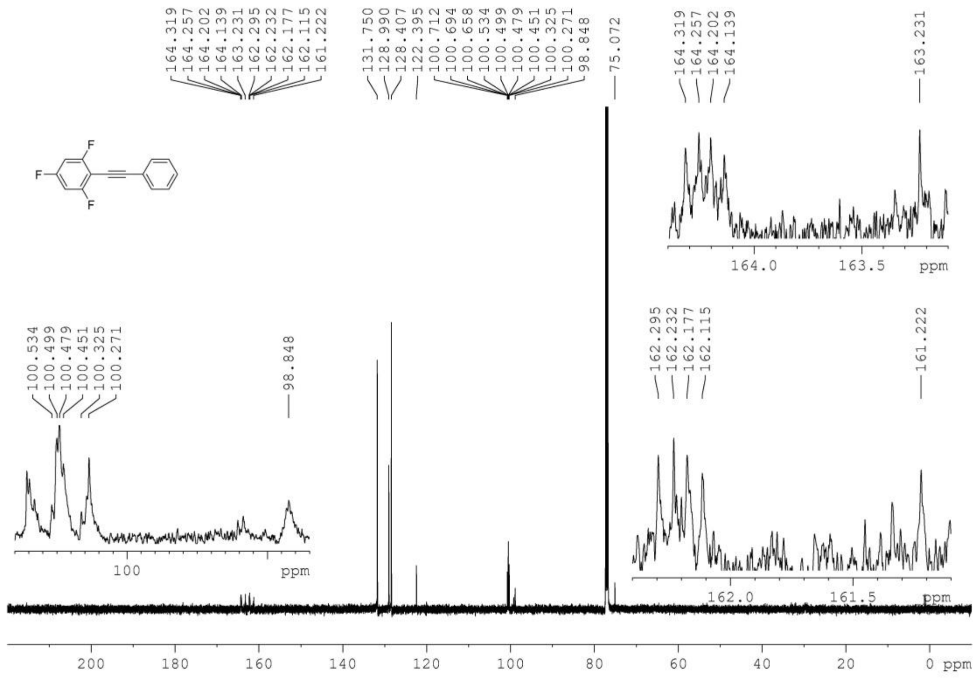

Compound 3e: ^{19}F NMR spectrum (470 MHz, $CDCl_3$).

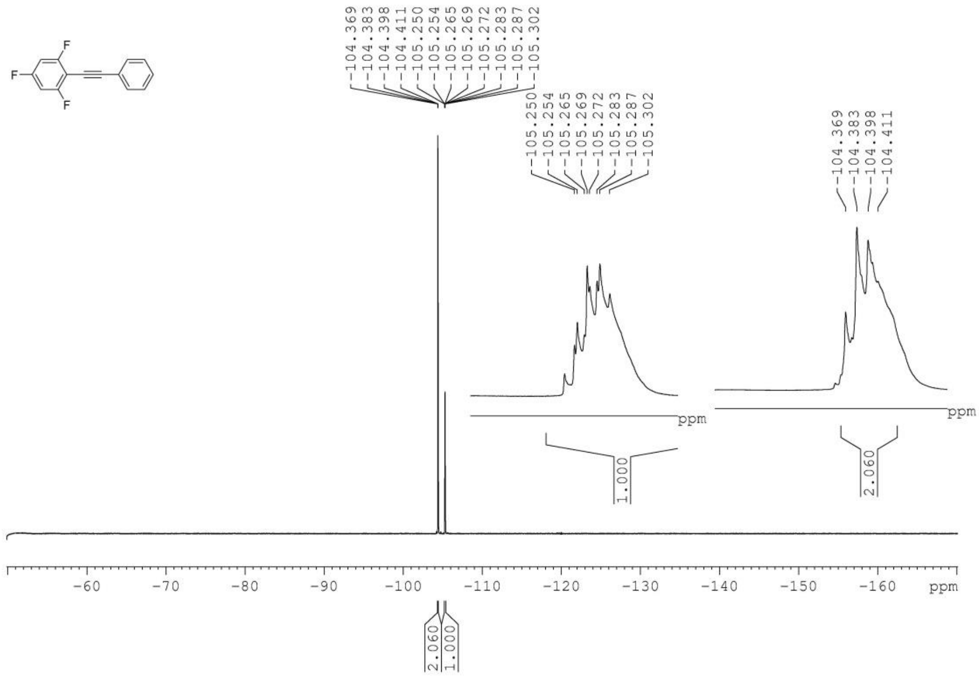

Compound 3f: ^{1}H NMR spectrum (500 MHz, $CDCl_3$).

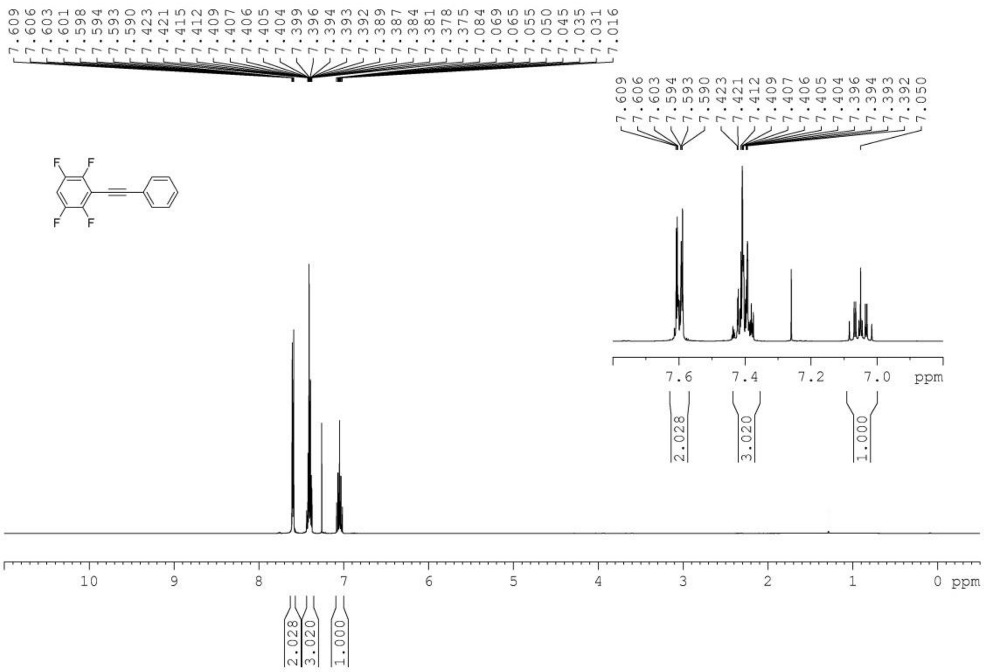

Compound 3f: $^{13}C\{^1H\}$ NMR spectrum (125 MHz, $CDCl_3$).

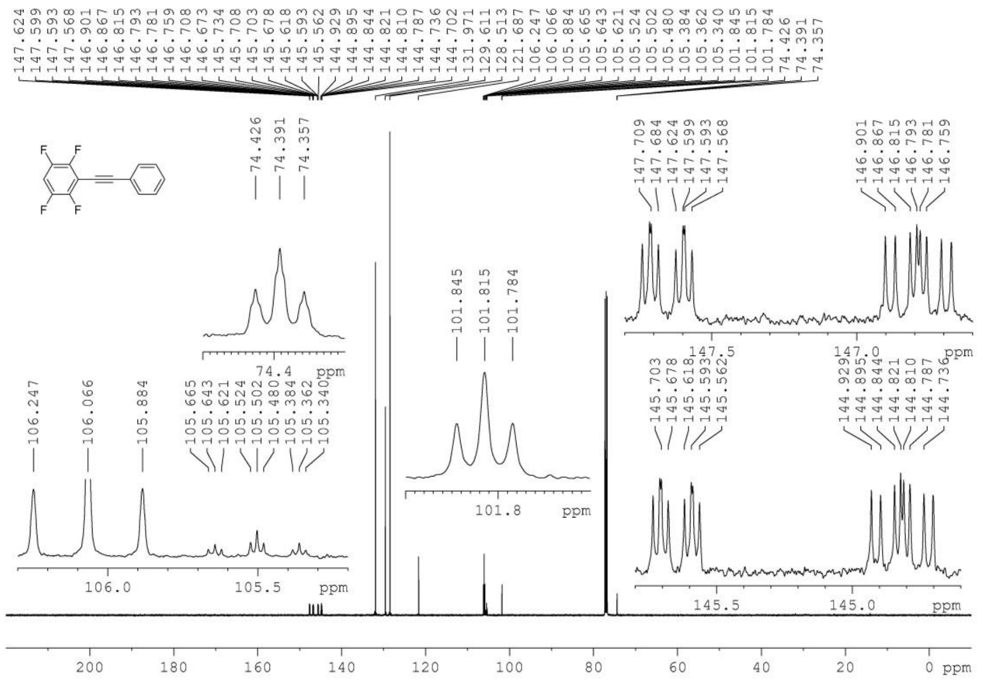

Compound 3f: ^{19}F NMR spectrum (470 MHz, $CDCl_3$).

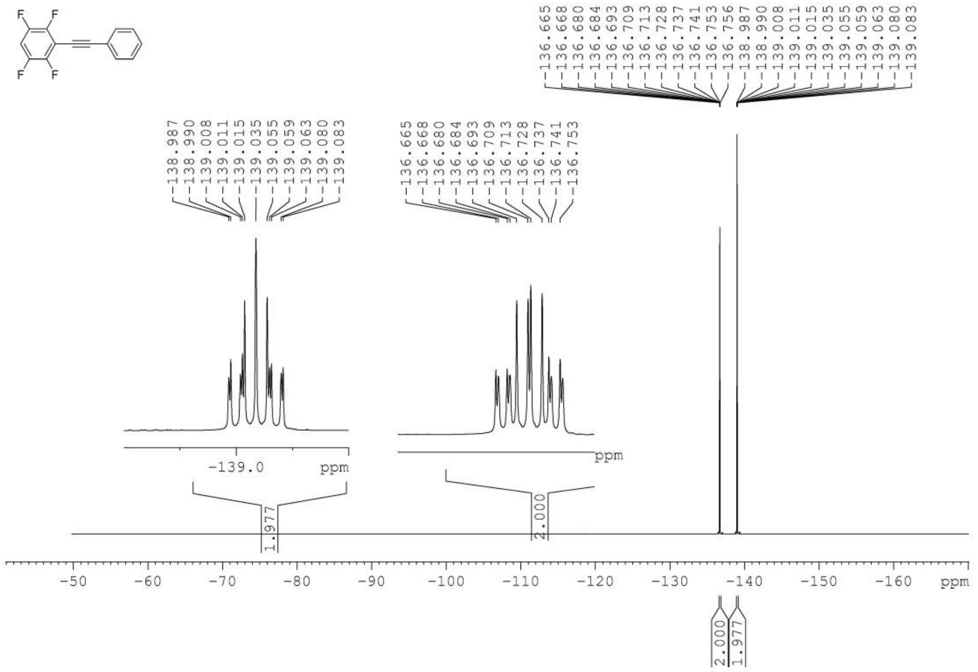

Compound 3g: ^{1}H NMR spectrum (500 MHz, $CDCl_3$).

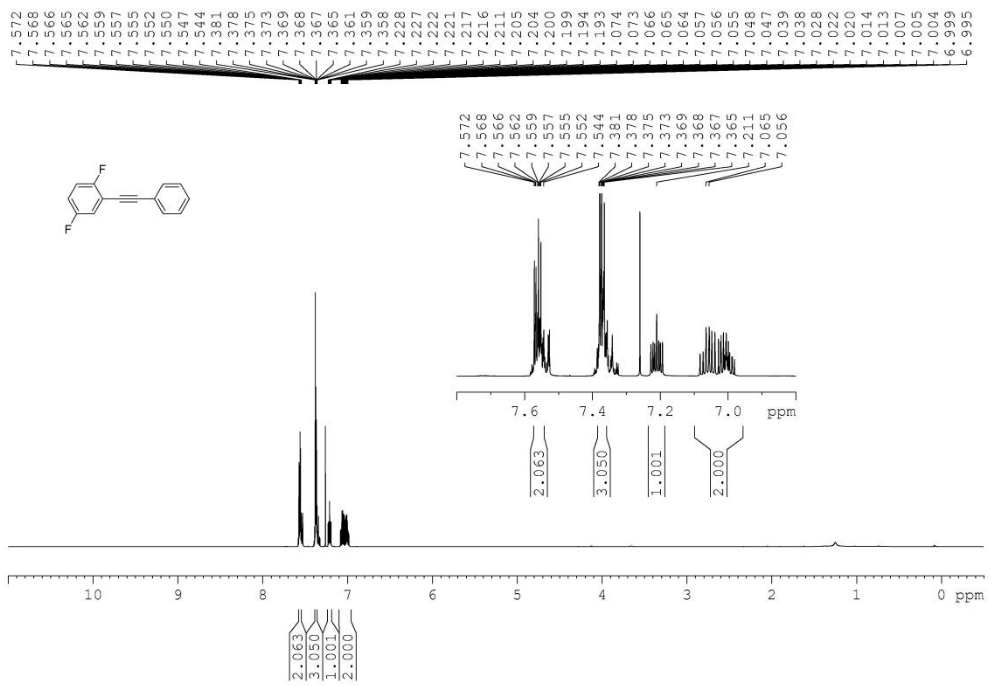

Compound 3g: $^{13}C\{^{1}H\}$ NMR spectrum (125 MHz, $CDCl_3$).

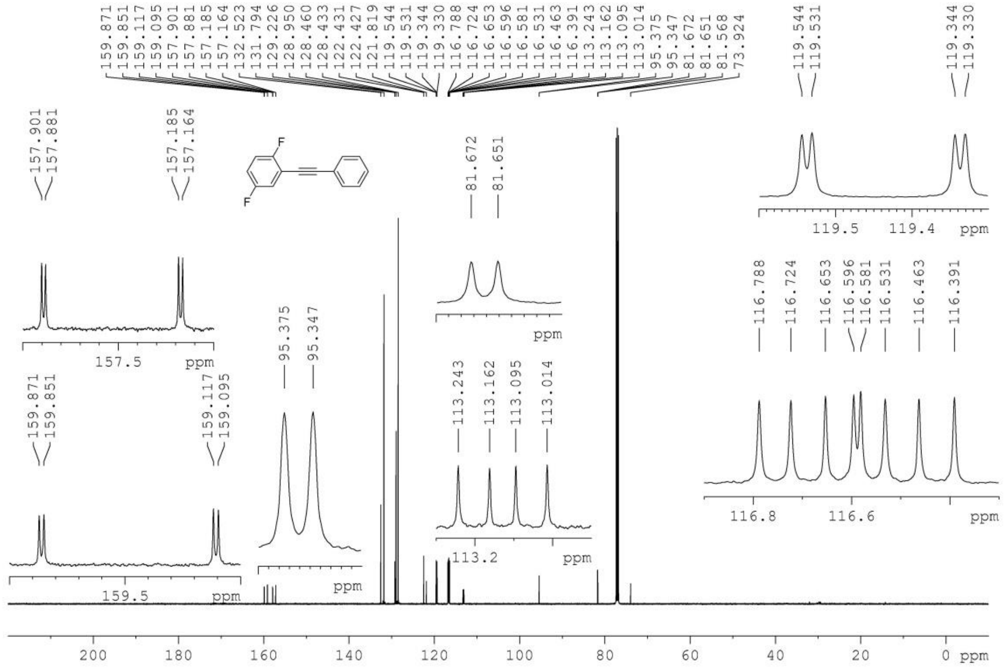

Compound 3g: ^{19}F NMR spectrum (470 MHz, $CDCl_3$).

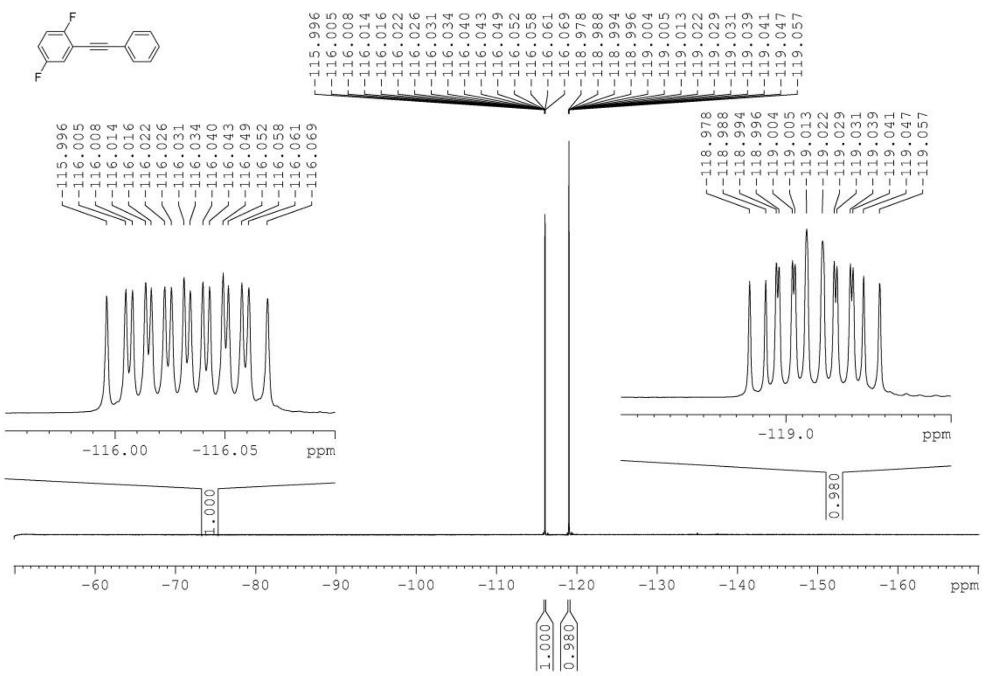

Compound 3h: ^{1}H NMR spectrum (500 MHz, $CDCl_3$).

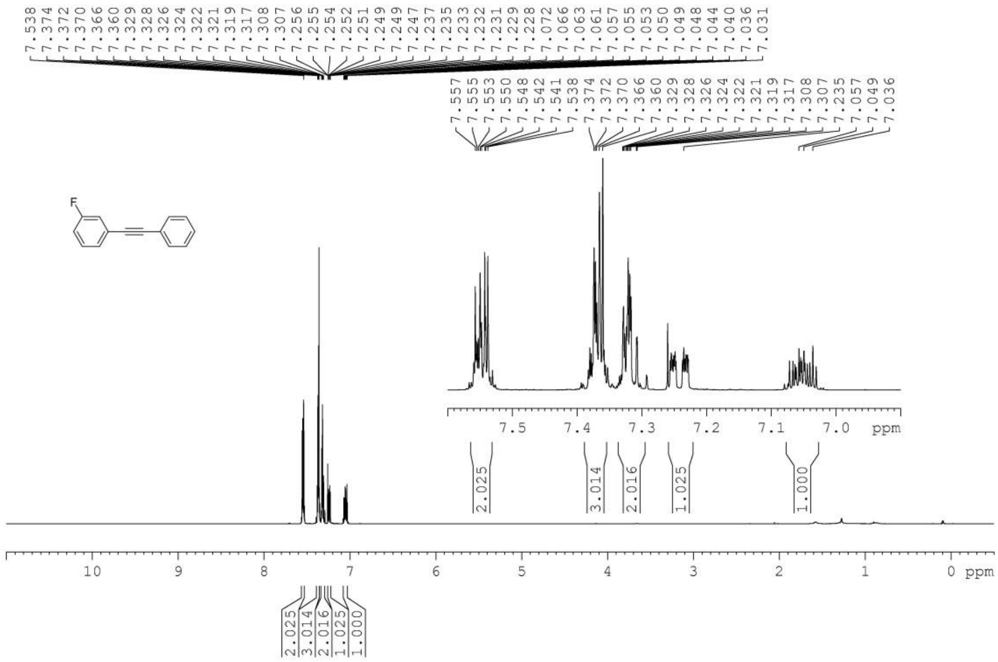

Compound 3h: $^{13}C\{^{1}H\}$ NMR spectrum (125 MHz, $CDCl_3$).

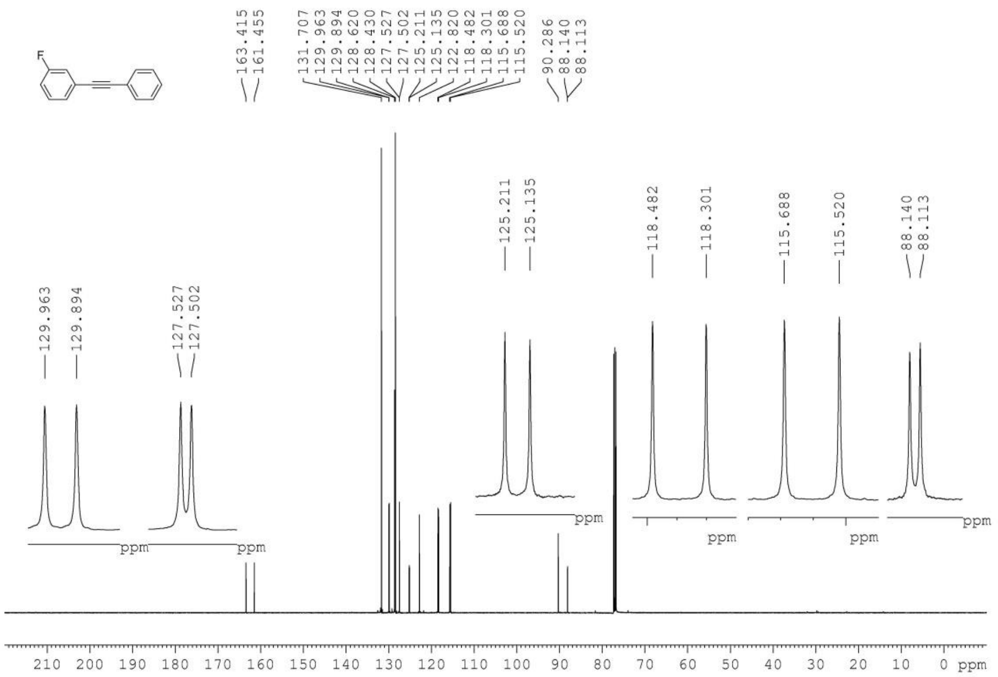

Compound 3h: ^{19}F NMR spectrum (470 MHz, $CDCl_3$).

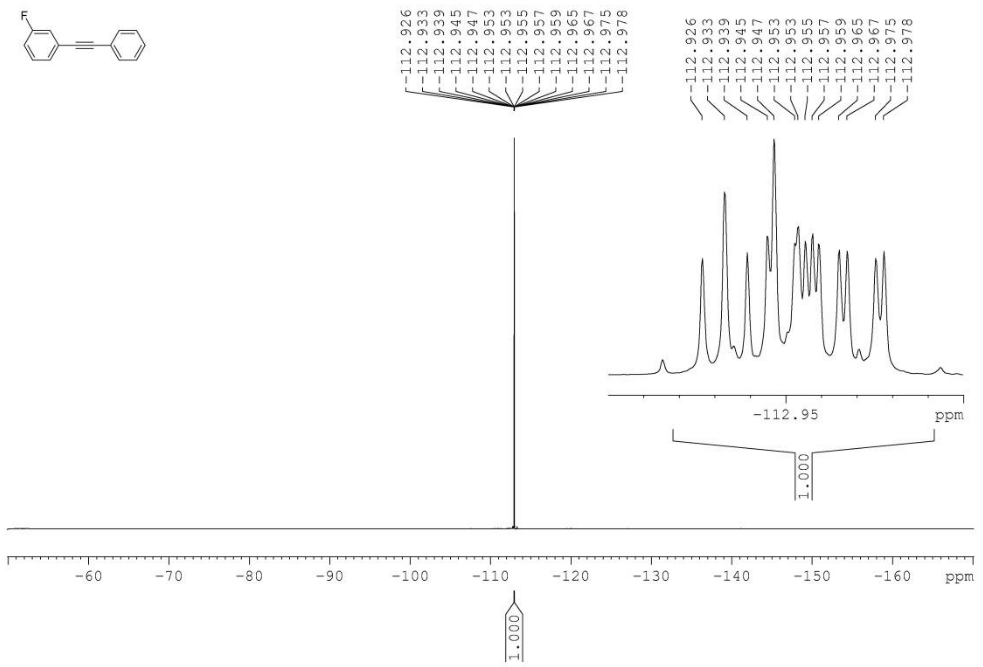

Compound 3i: ^{1}H NMR spectrum (500 MHz, CDCl$_3$).

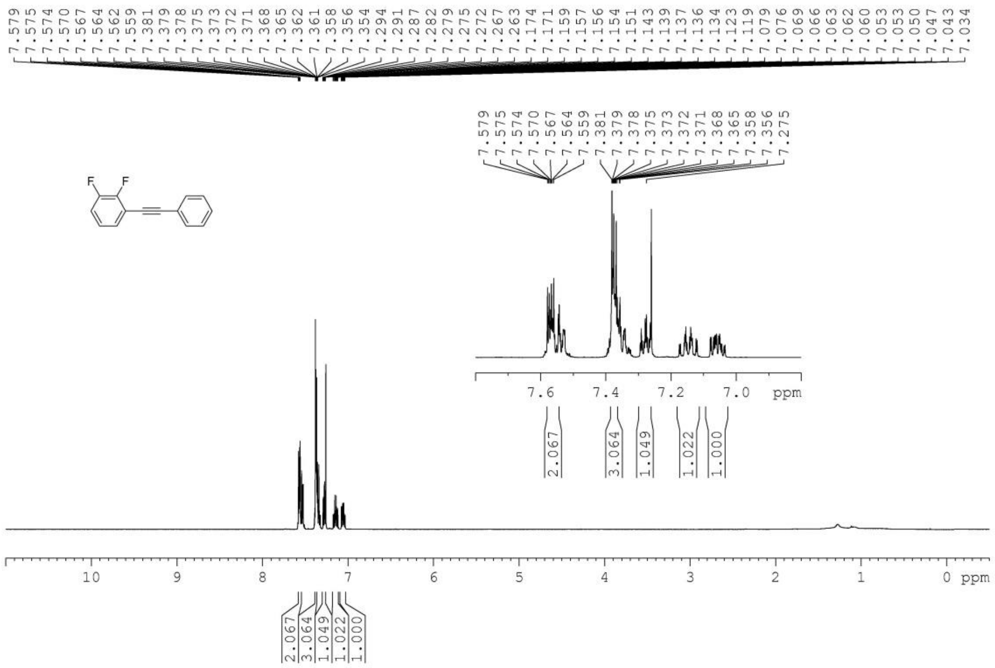

Compound 3i: ^{13}C{^{1}H} NMR spectrum (125 MHz, CDCl$_3$).

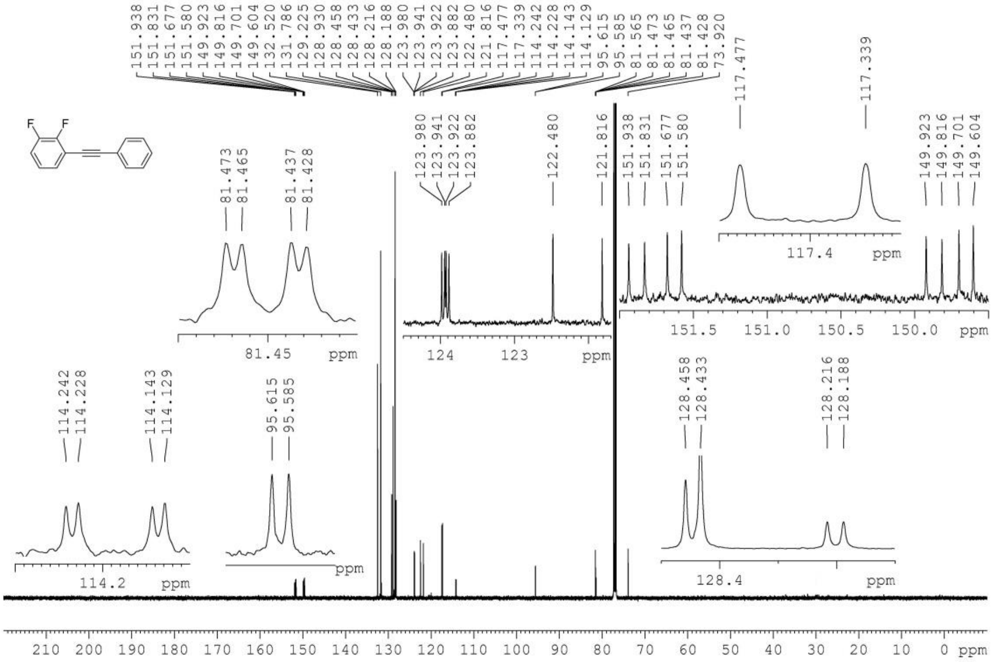

Compound 3i: ^{19}F NMR spectrum (470 MHz, $CDCl_3$).

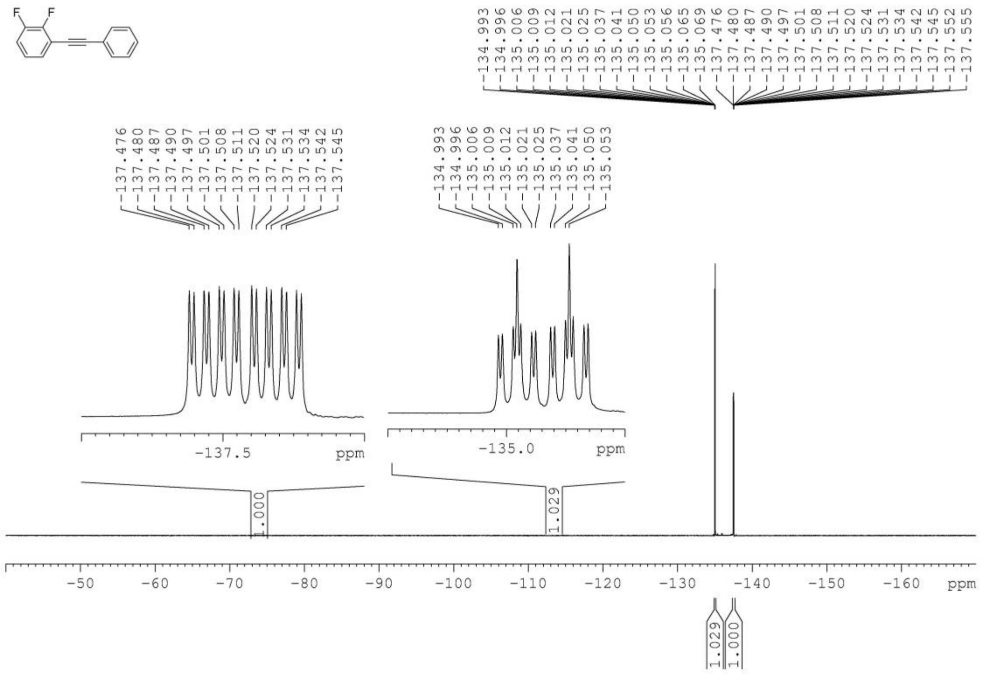

Compound 6a: ^{1}H NMR spectrum (500 MHz, $CDCl_3$).

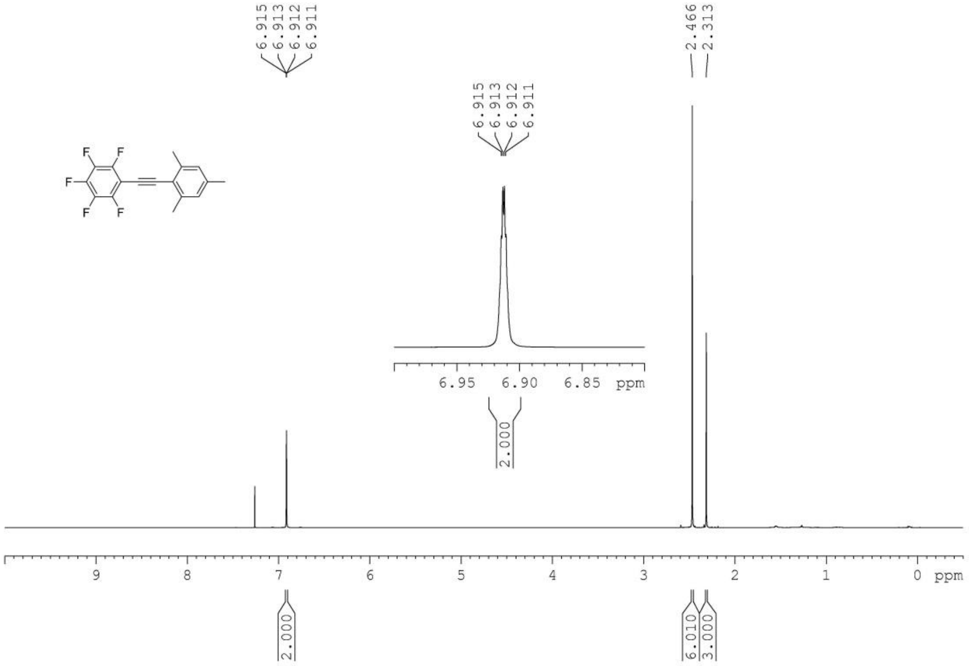

Compound 6a: $^{13}C\{^{1}H\}$ NMR spectrum (125 MHz, $CDCl_3$).

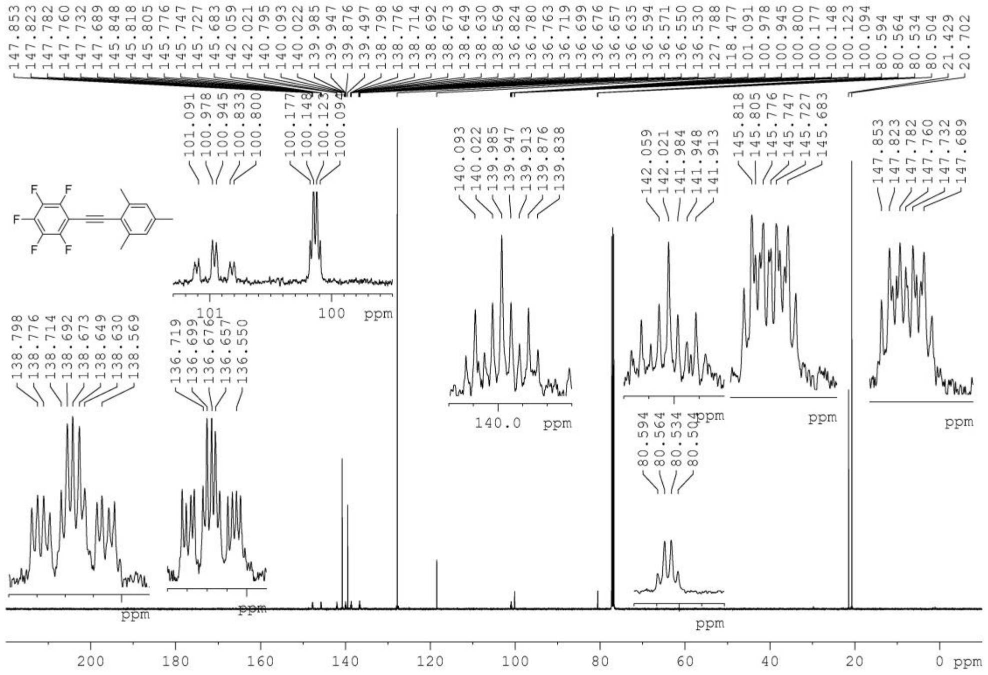

Compound 6a: ^{19}F NMR spectrum (470 MHz, $CDCl_3$).

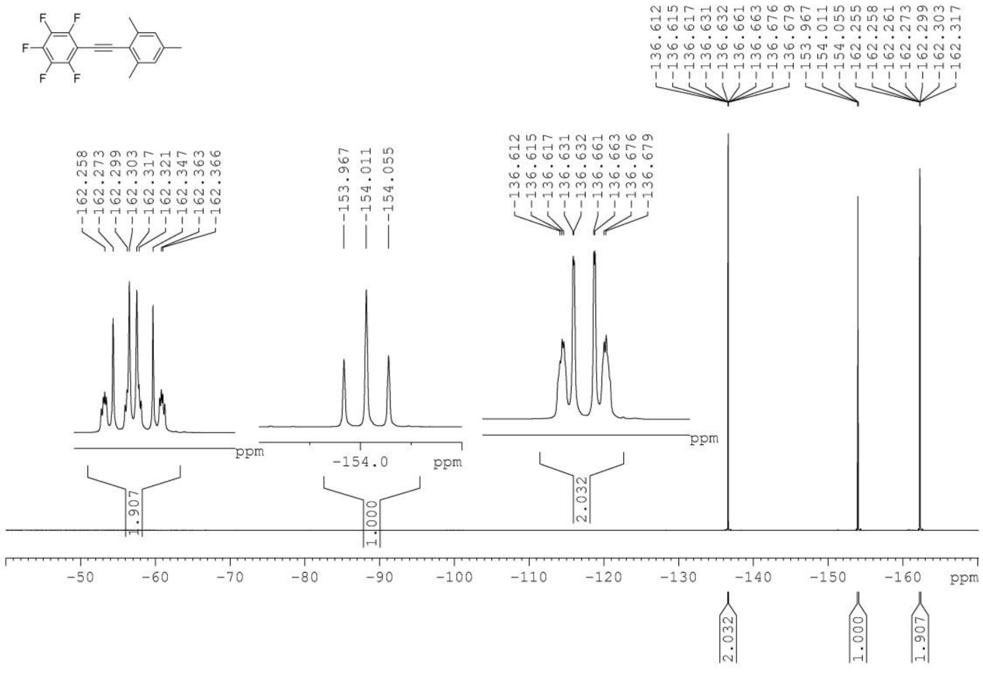

Compound 6b: ^{1}H NMR spectrum (500 MHz, $CDCl_3$).

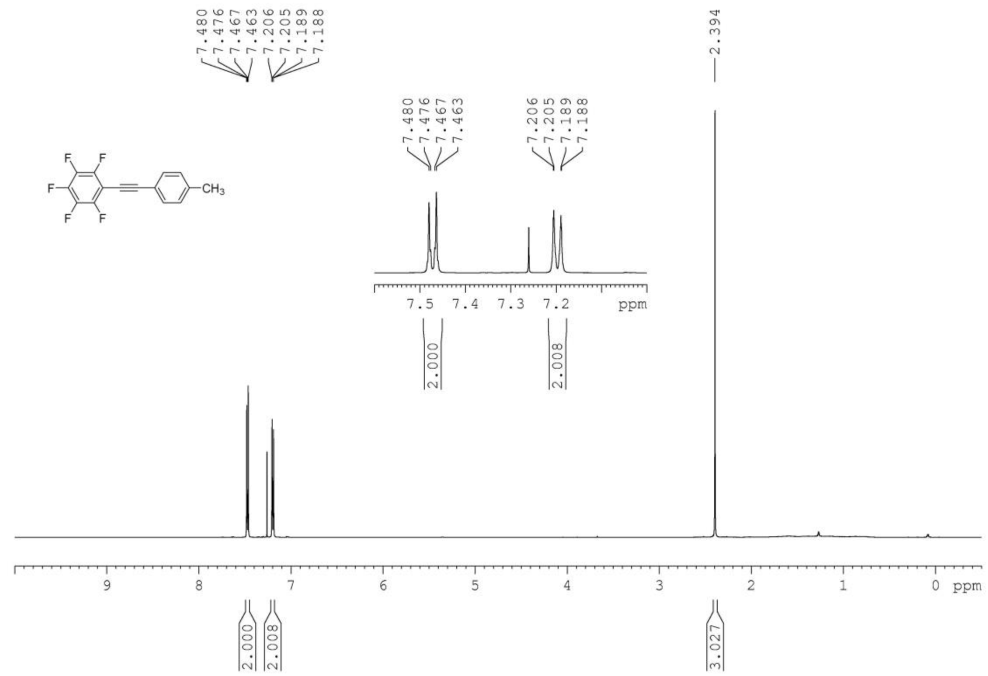

Compound 6b: $^{13}C\{^{1}H\}$ NMR spectrum (125 MHz, $CDCl_3$).

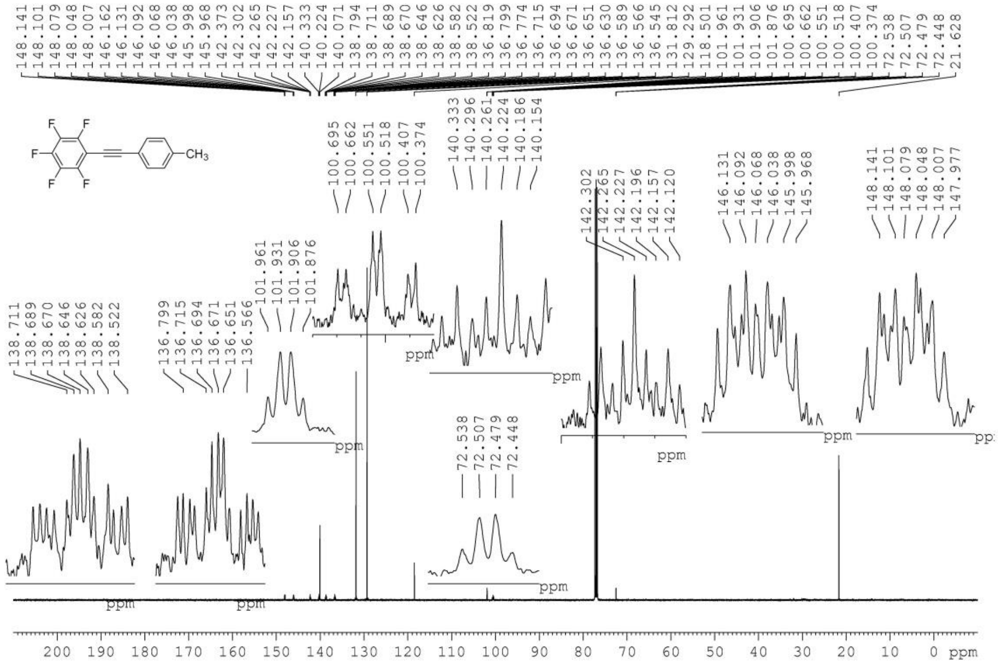

Compound 6b: ^{19}F NMR spectrum (470 MHz, $CDCl_3$).

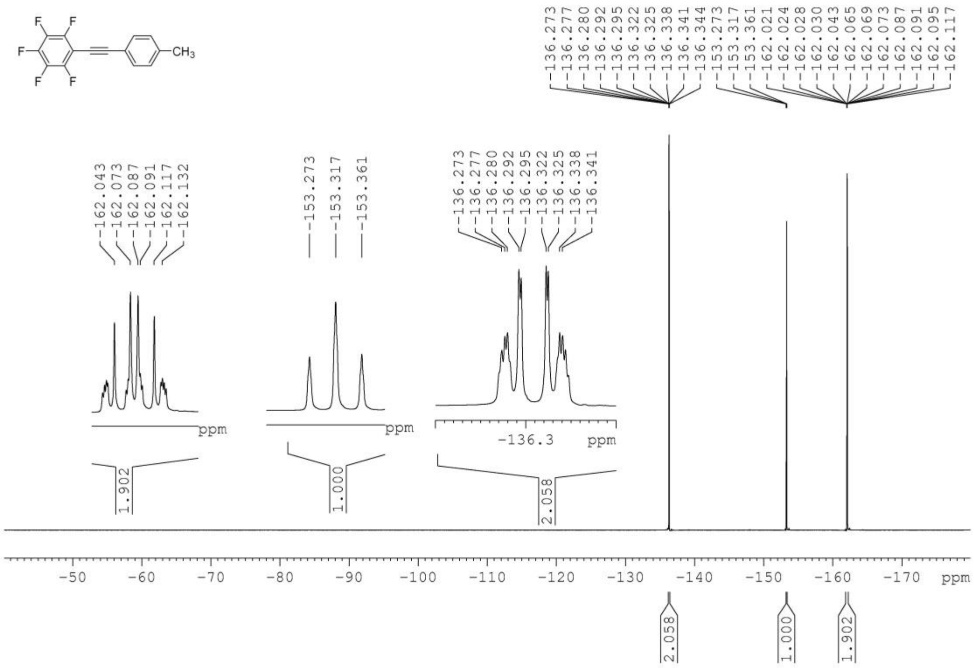

Compound 6c: ^{1}H NMR spectrum (500 MHz, $CDCl_3$).

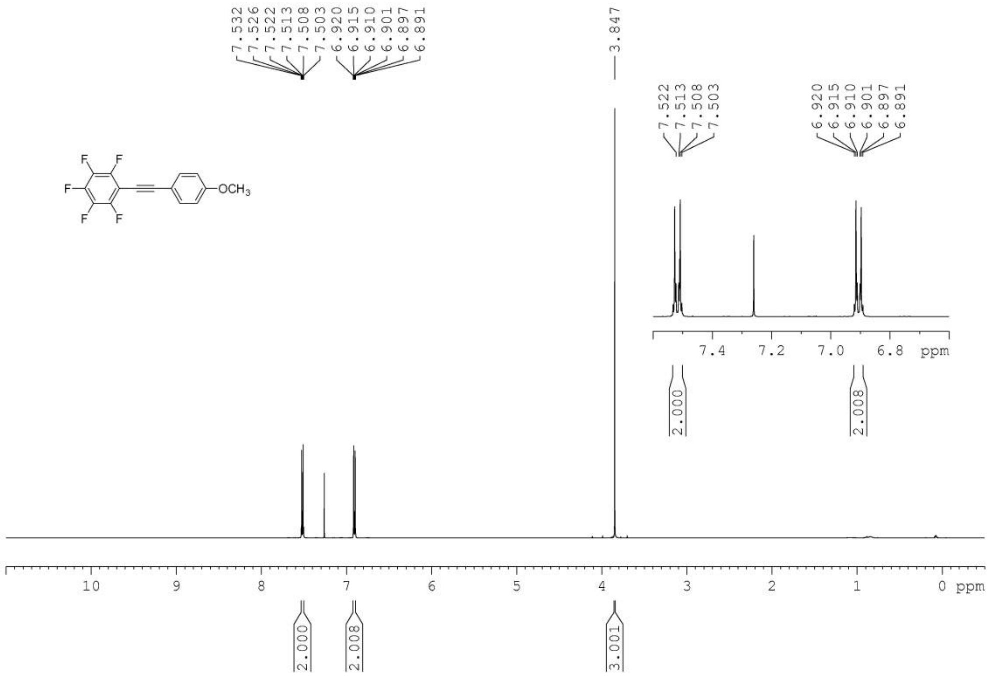

Compound 6c: $^{13}C\{^{1}H\}$ NMR spectrum (125 MHz, $CDCl_3$).

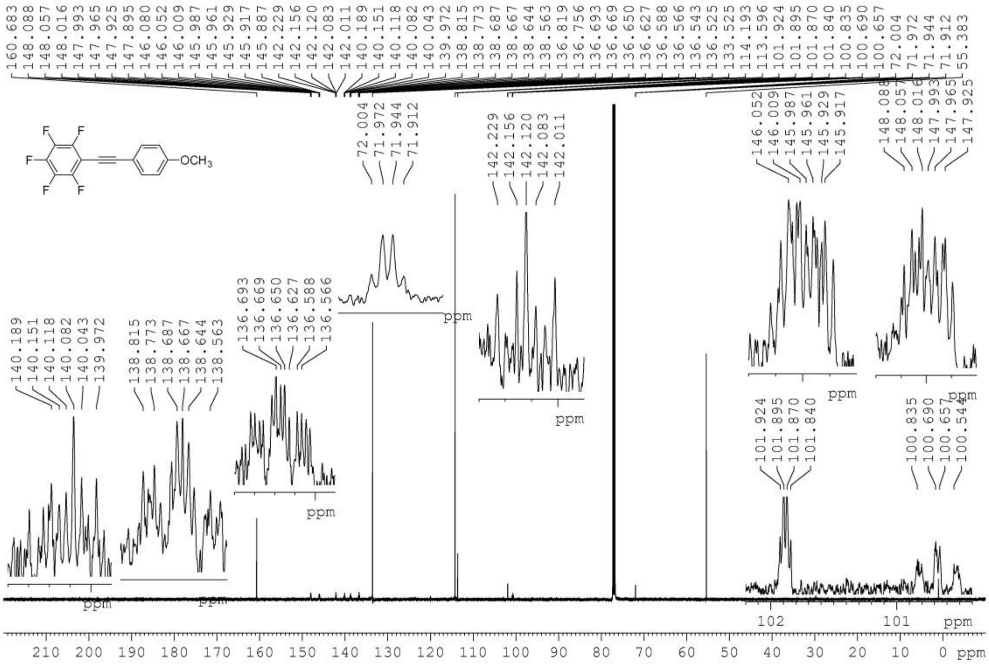

Compound 6c: ^{19}F NMR spectrum (470 MHz, $CDCl_3$).

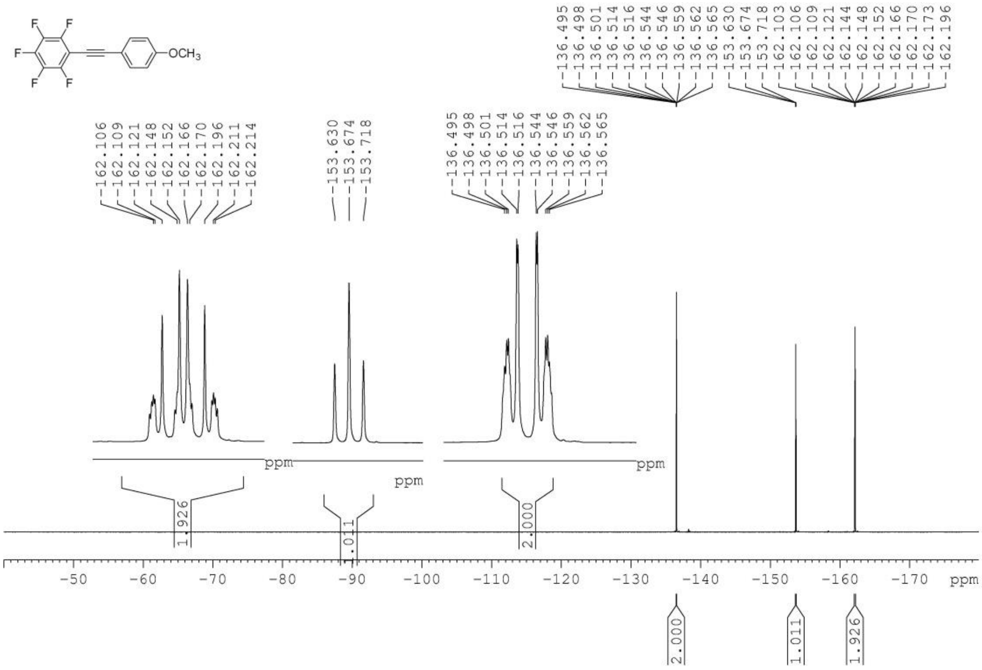

Compound 6d: ^{1}H NMR spectrum (500 MHz, $CDCl_3$).

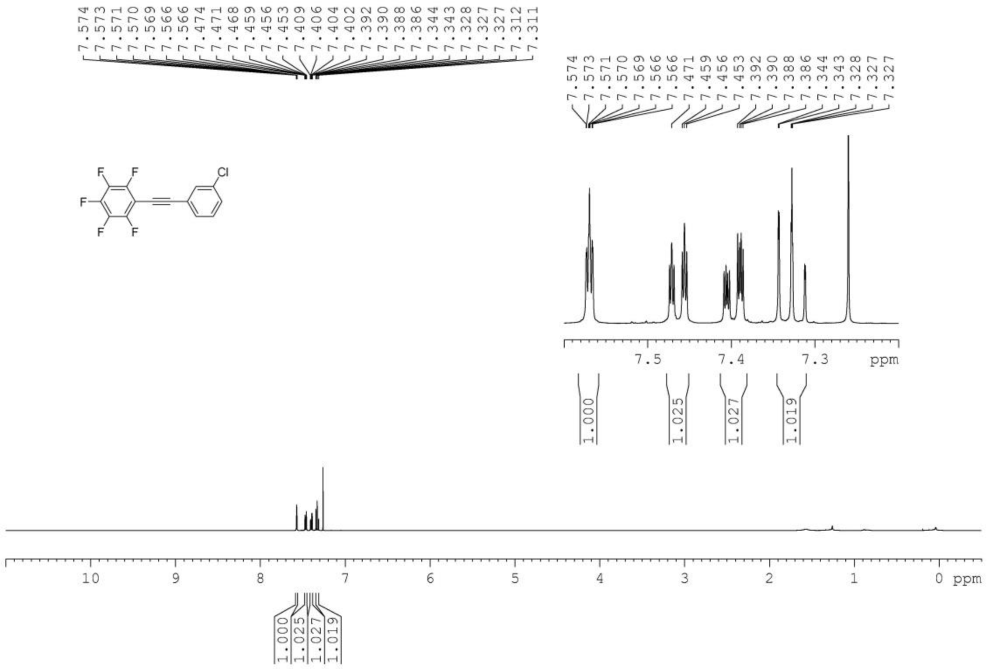

Compound 6d: ^{13}C{^{1}H} NMR spectrum (125 MHz, $CDCl_3$).

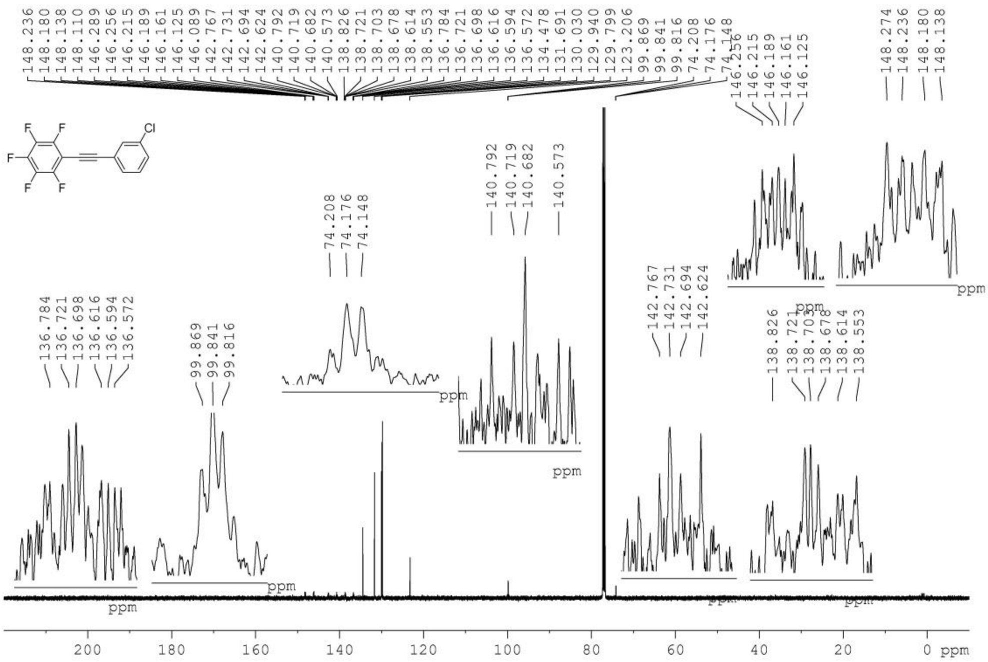

Compound 6d: ^{19}F NMR spectrum (470 MHz, $CDCl_3$).

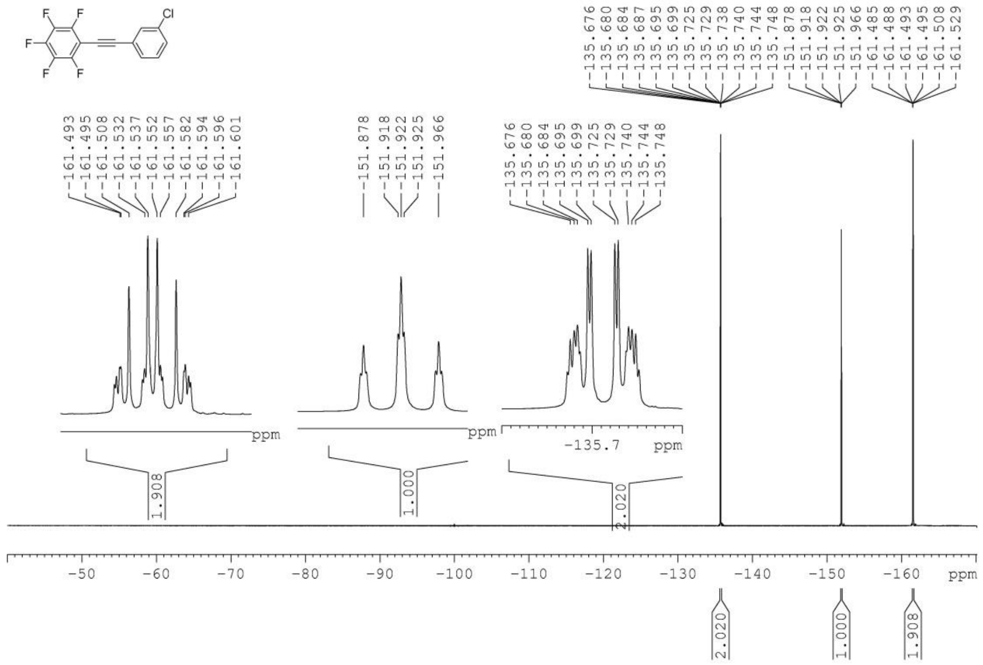

Compound 6e: ^{1}H NMR spectrum (500 MHz, $CDCl_3$).

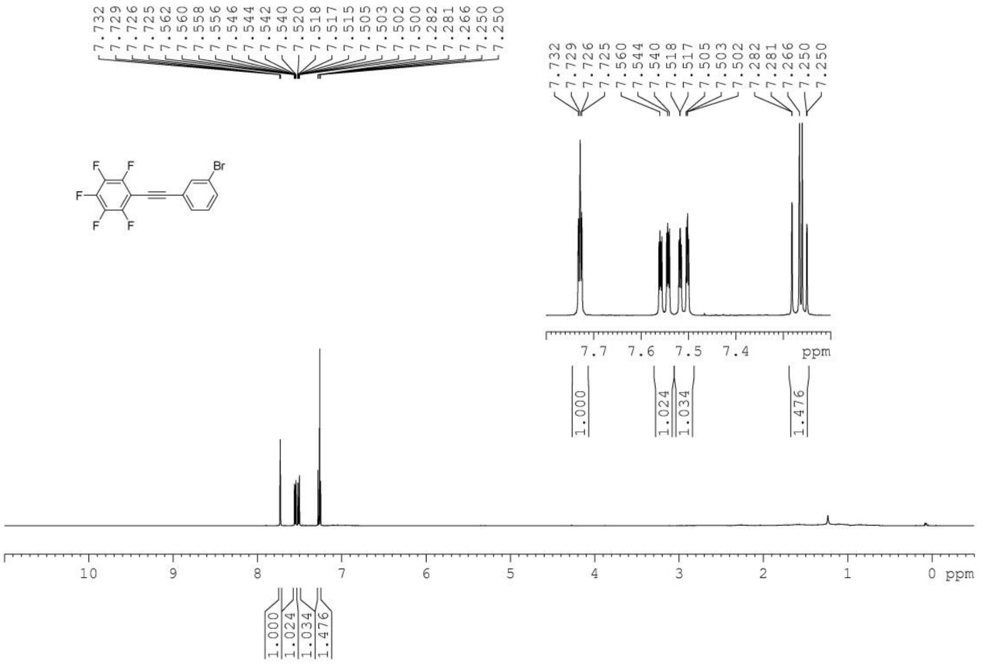

Compound 6e: $^{13}C\{^1H\}$ NMR spectrum (125 MHz, $CDCl_3$).

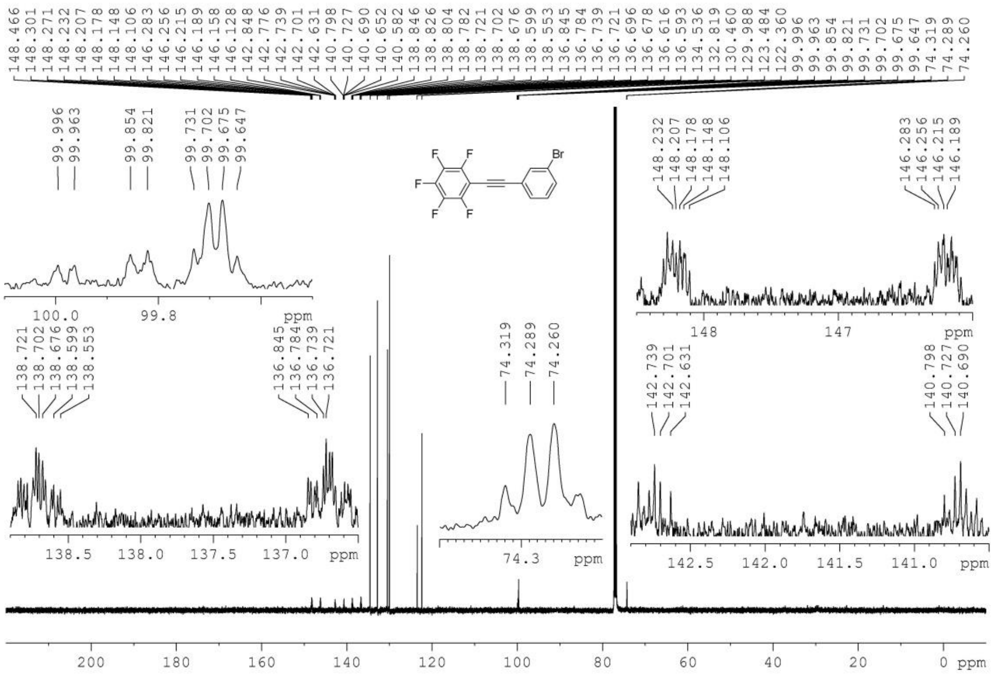

Compound 6e: ^{19}F NMR spectrum (470 MHz, $CDCl_3$).

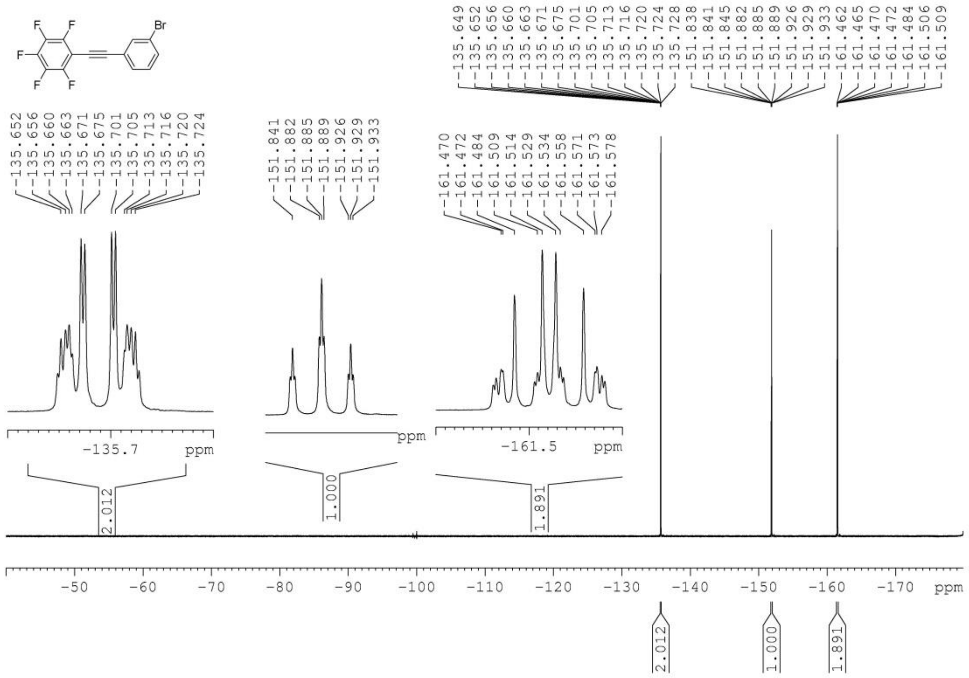

Compound 6f: ^{1}H NMR spectrum (500 MHz, $CDCl_3$).

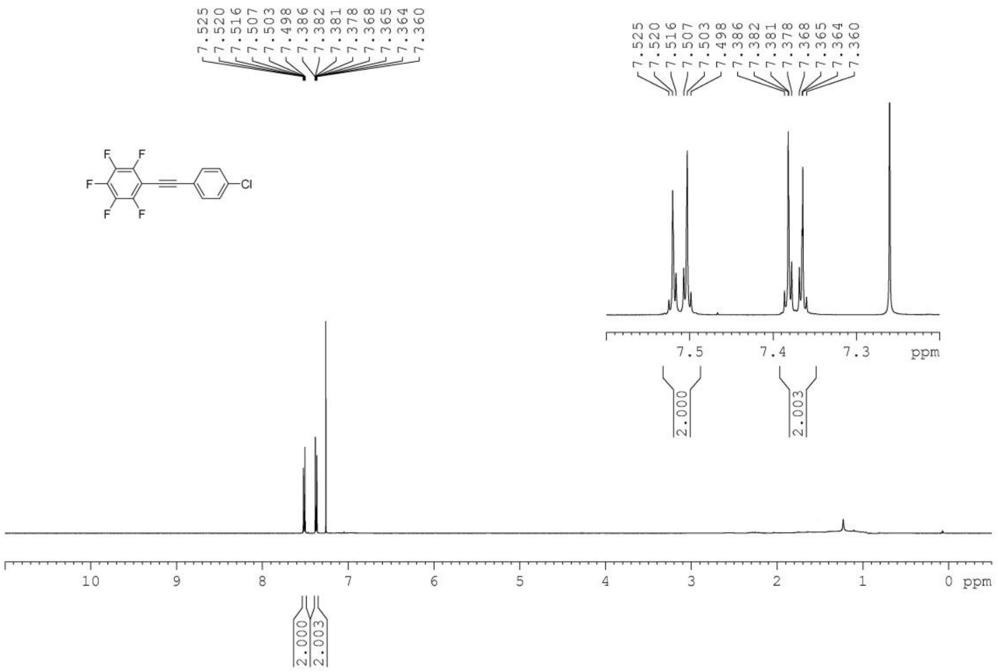

Compound 6f: ^{13}C{^{1}H} NMR spectrum (125 MHz, $CDCl_3$).

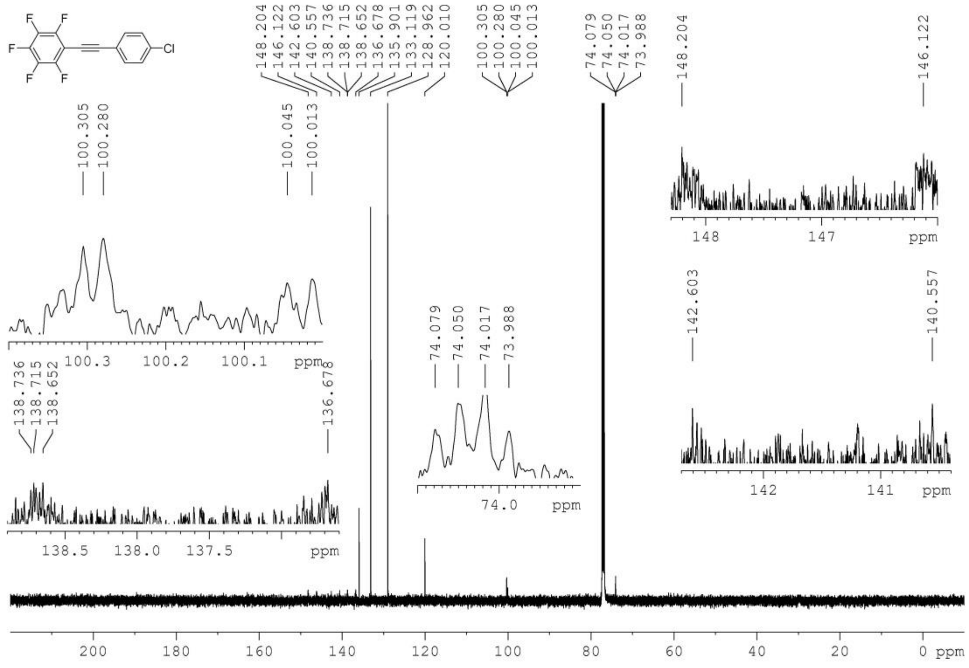

Compound 6f: ^{19}F NMR spectrum (470 MHz, $CDCl_3$).

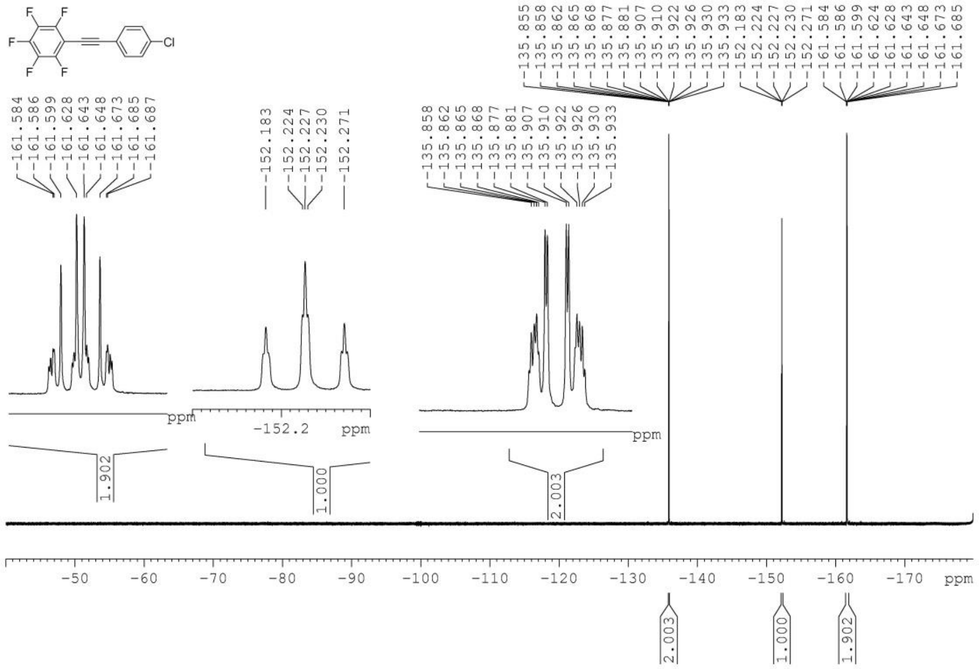

Compound 6g: ^{1}H NMR spectrum (500 MHz, $CDCl_3$).

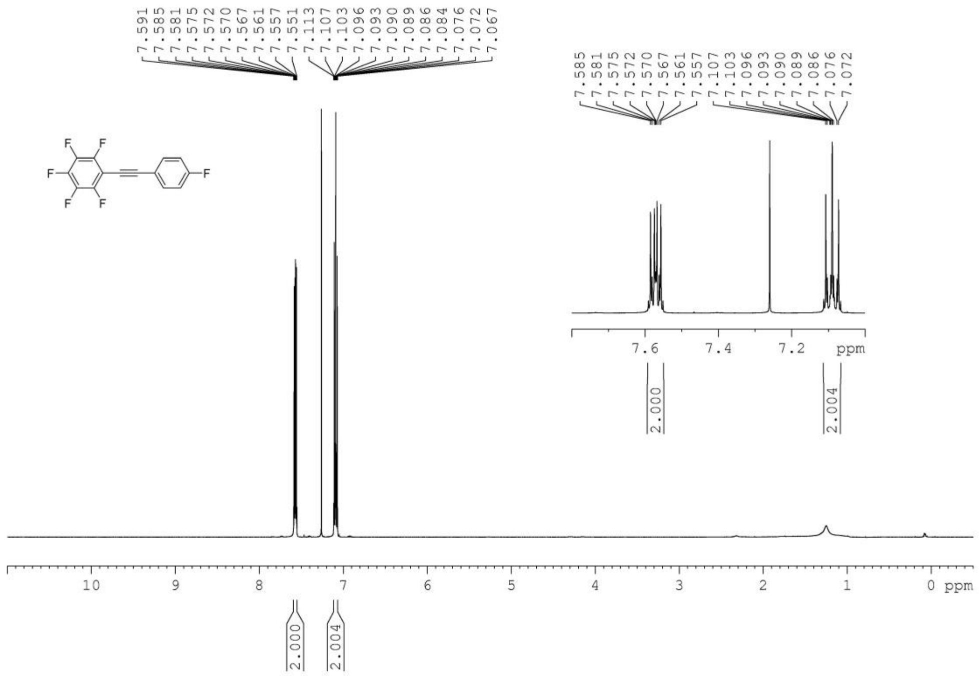

Compound 6g: $^{13}C\{^{1}H\}$ NMR spectrum (125 MHz, $CDCl_3$).

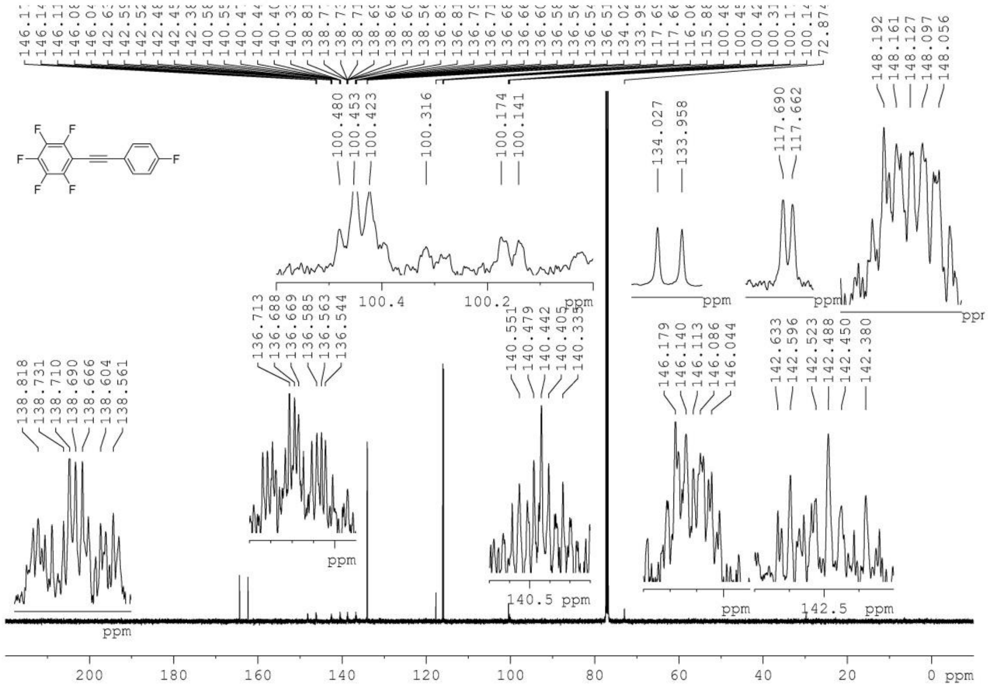

Compound 6g: ^{19}F NMR spectrum (470 MHz, $CDCl_3$).

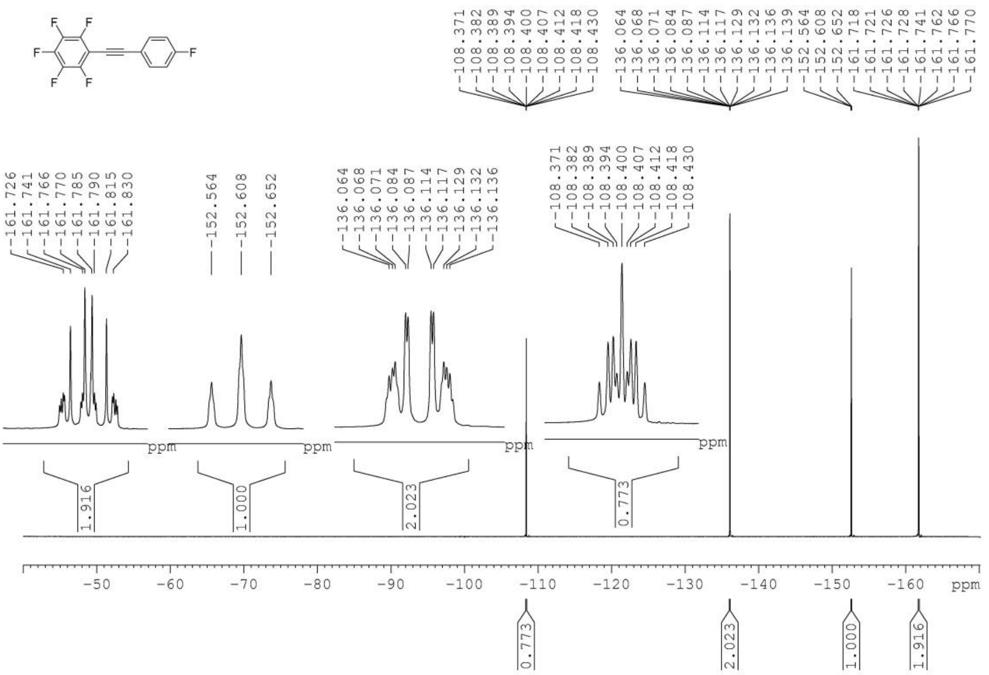

Compound 6h: ^{1}H NMR spectrum (500 MHz, $CDCl_3$).

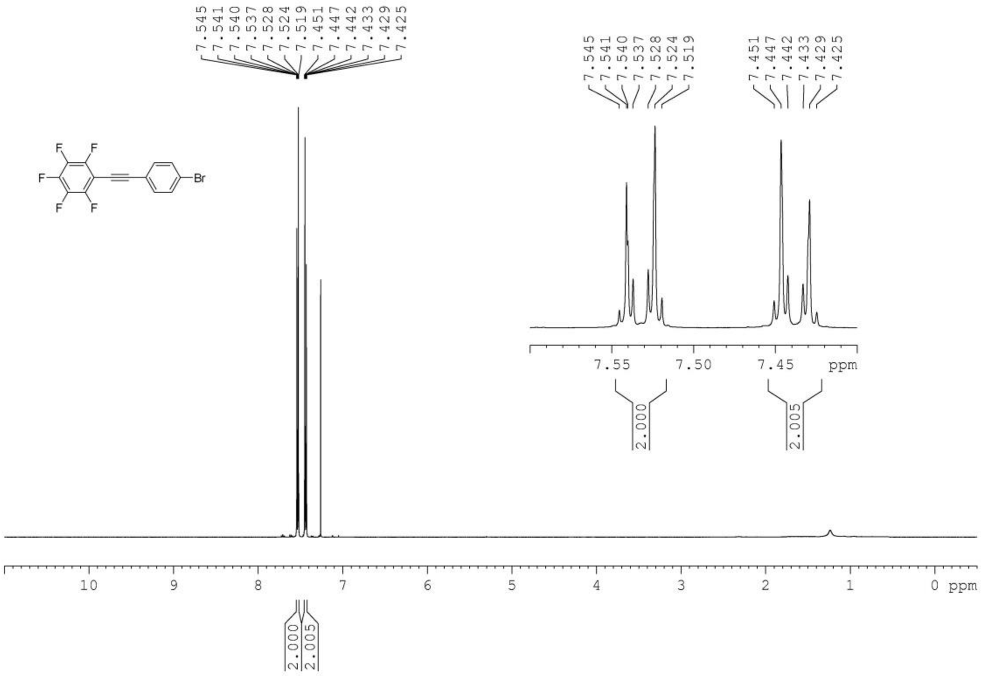

Compound 6h: ^{13}C{^{1}H} NMR spectrum (125 MHz, $CDCl_3$).

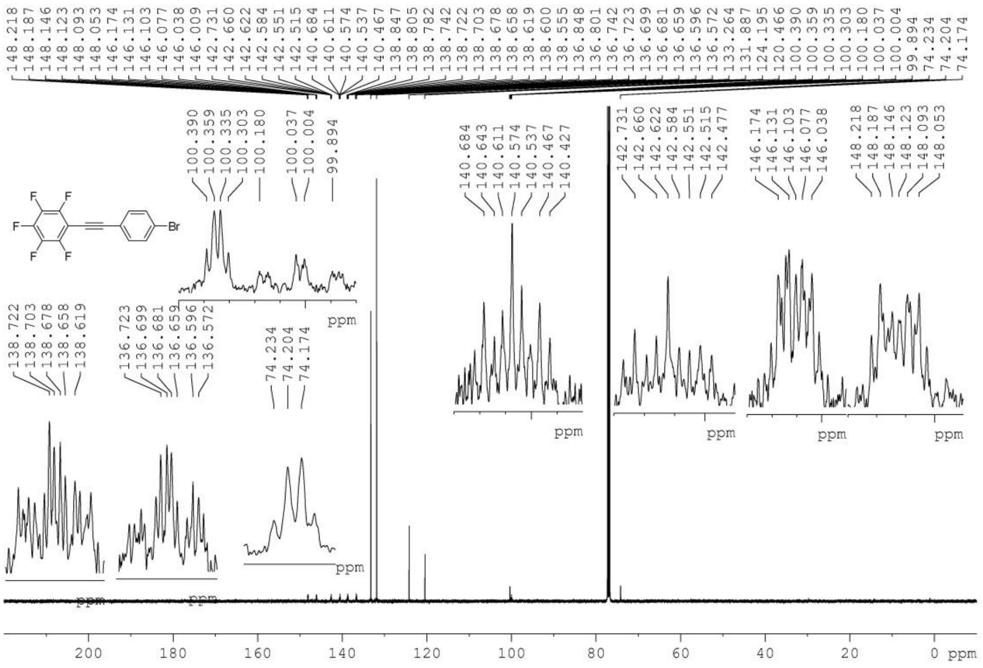

Compound 6h: ¹⁹F NMR spectrum (470 MHz, CDCl$_3$).

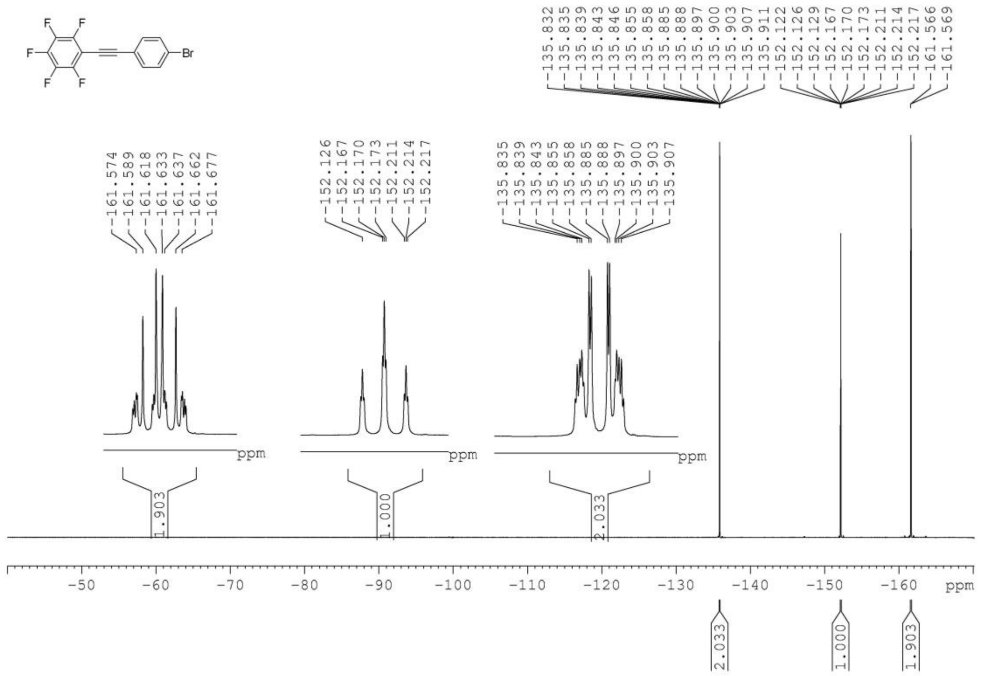

Compound 6i: ¹H NMR spectrum (500 MHz, CDCl$_3$).

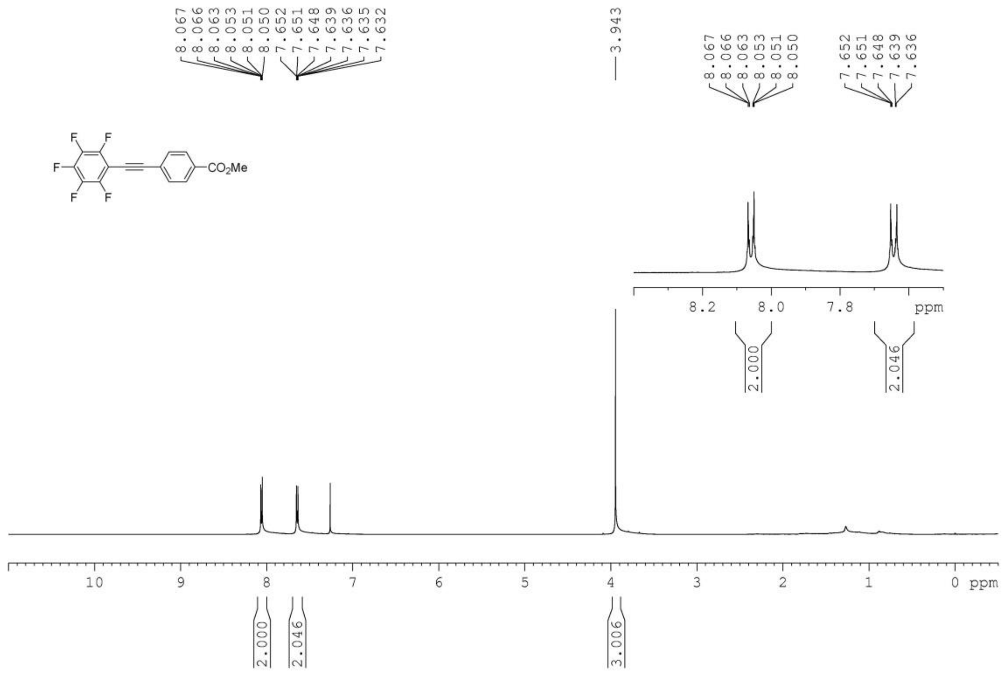

Compound 6i: $^{13}C\{^{1}H\}$ NMR spectrum (125 MHz, $CDCl_3$).

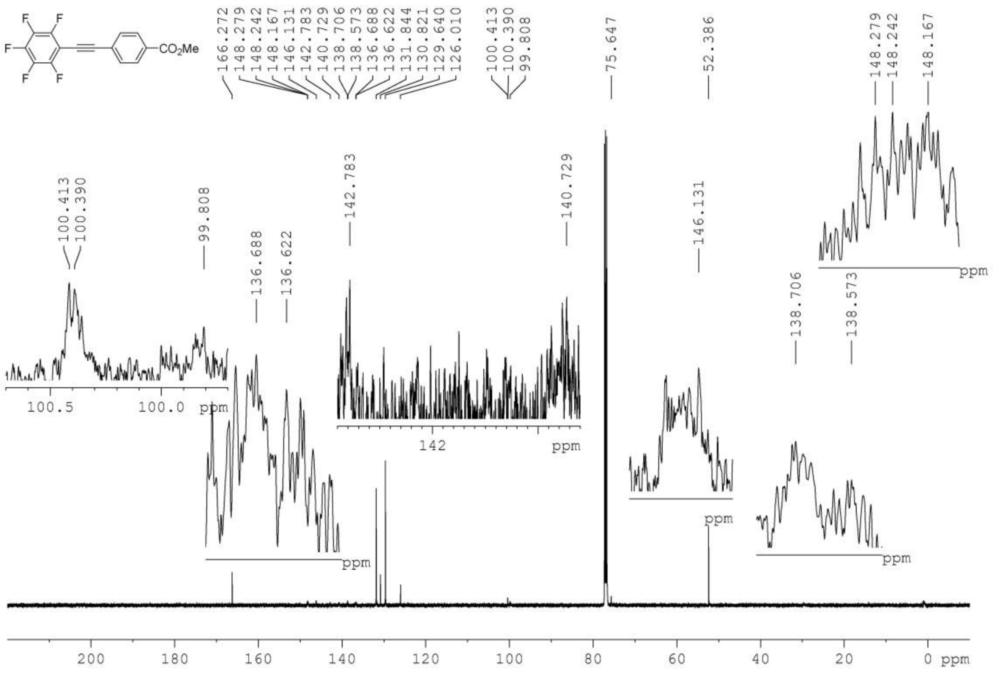

Compound 6i: ^{19}F NMR spectrum (470 MHz, $CDCl_3$).

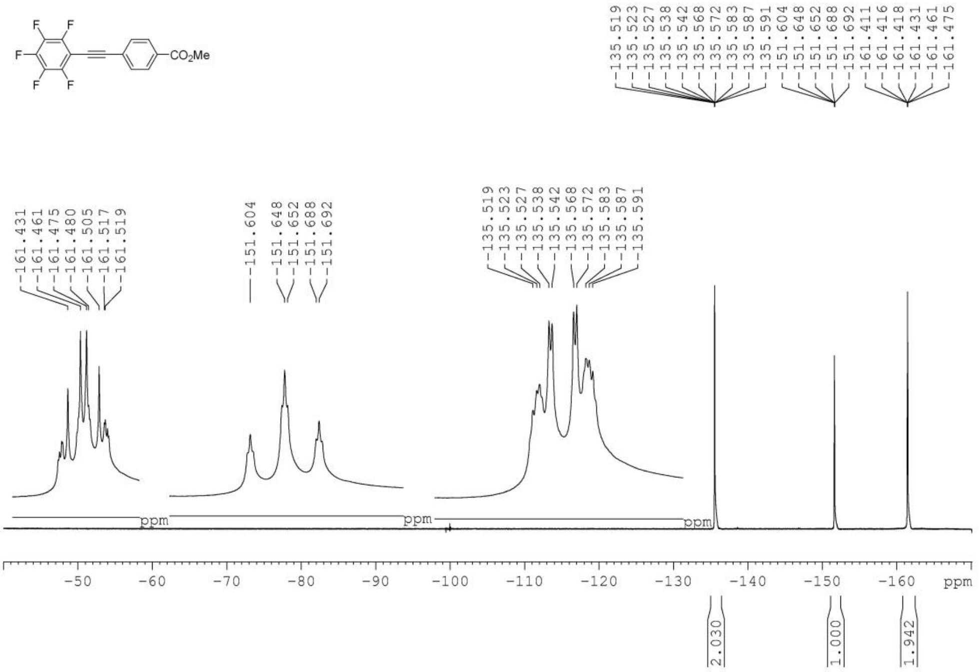

Compound 6j: ^{1}H NMR spectrum (500 MHz, $CDCl_3$).

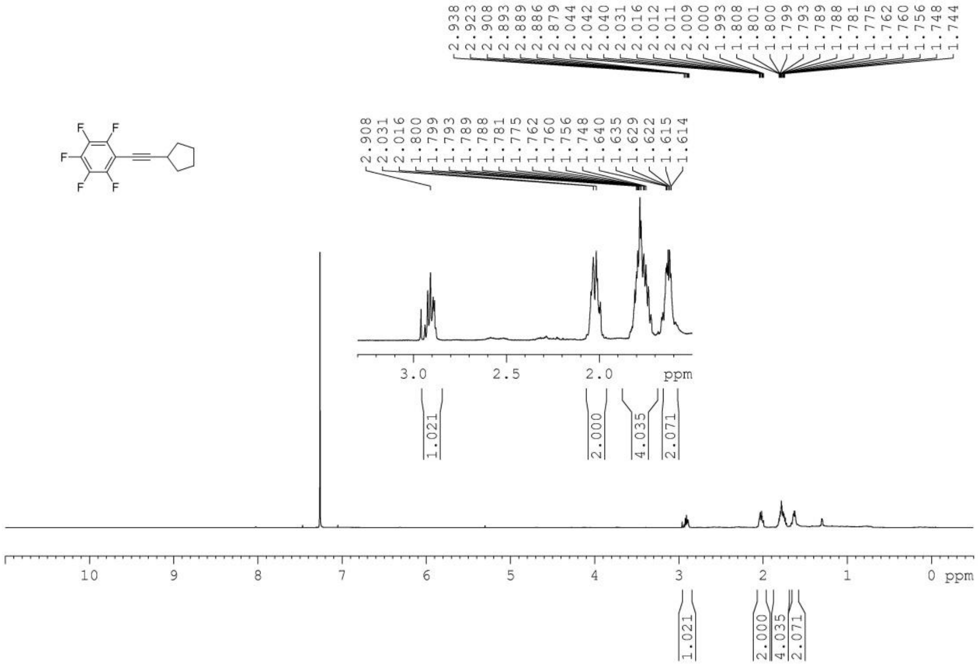

Compound 6j: ^{13}C{^{1}H} NMR spectrum (125 MHz, $CDCl_3$).

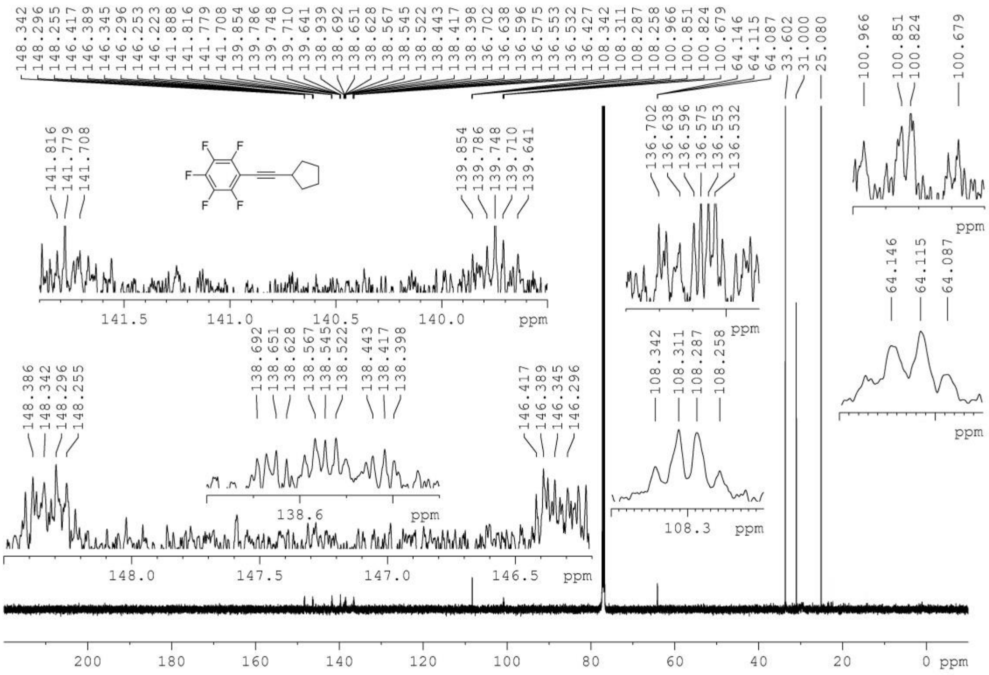

Compound 6j: ^{19}F NMR spectrum (470 MHz, $CDCl_3$).

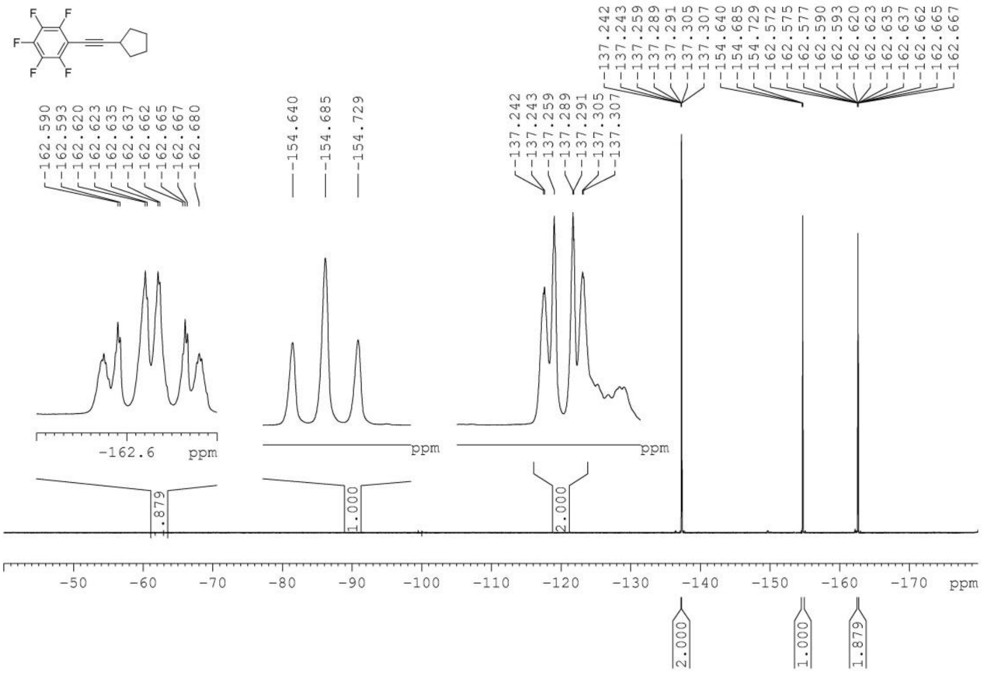

Compound 6k: ^{1}H NMR spectrum (500 MHz, $CDCl_3$).

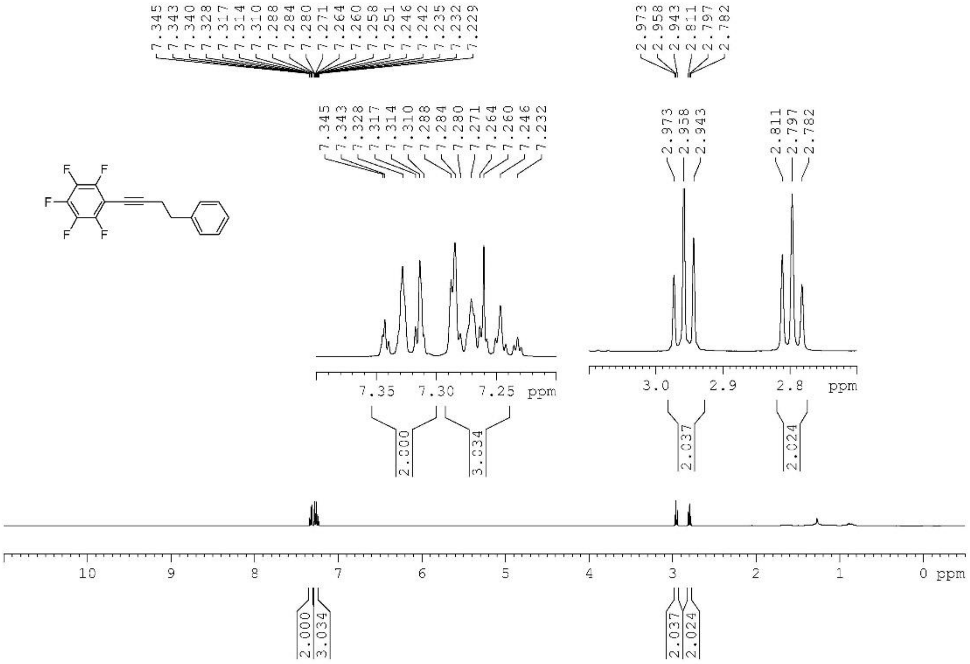

Compound 6k: $^{13}C\{^1H\}$ NMR spectrum (125 MHz, $CDCl_3$).

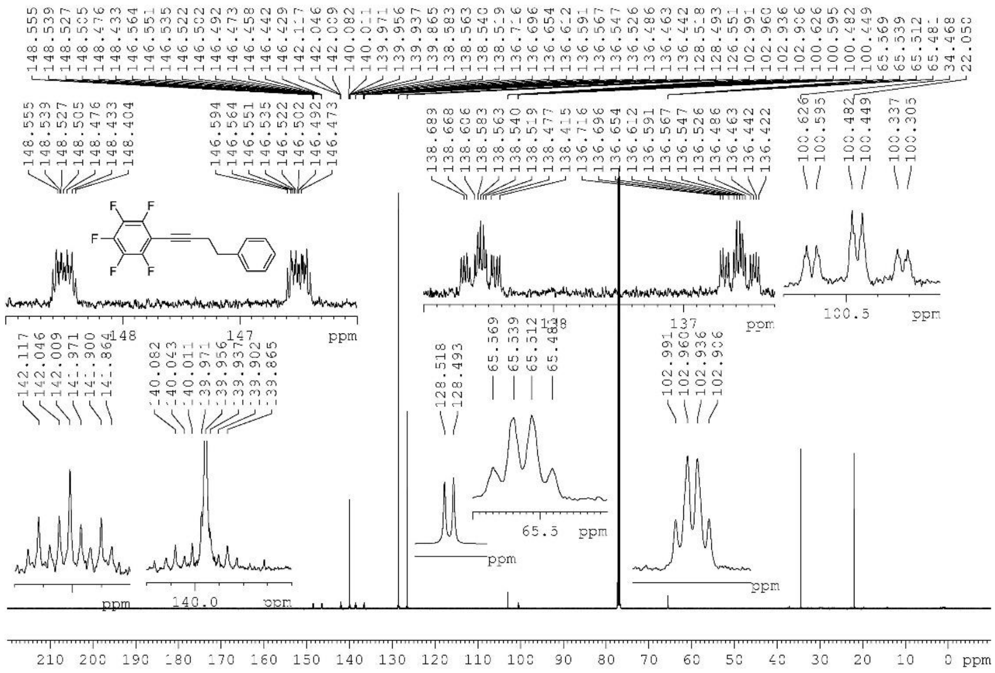

Compound 6k: ^{19}F NMR spectrum (470 MHz, $CDCl_3$).

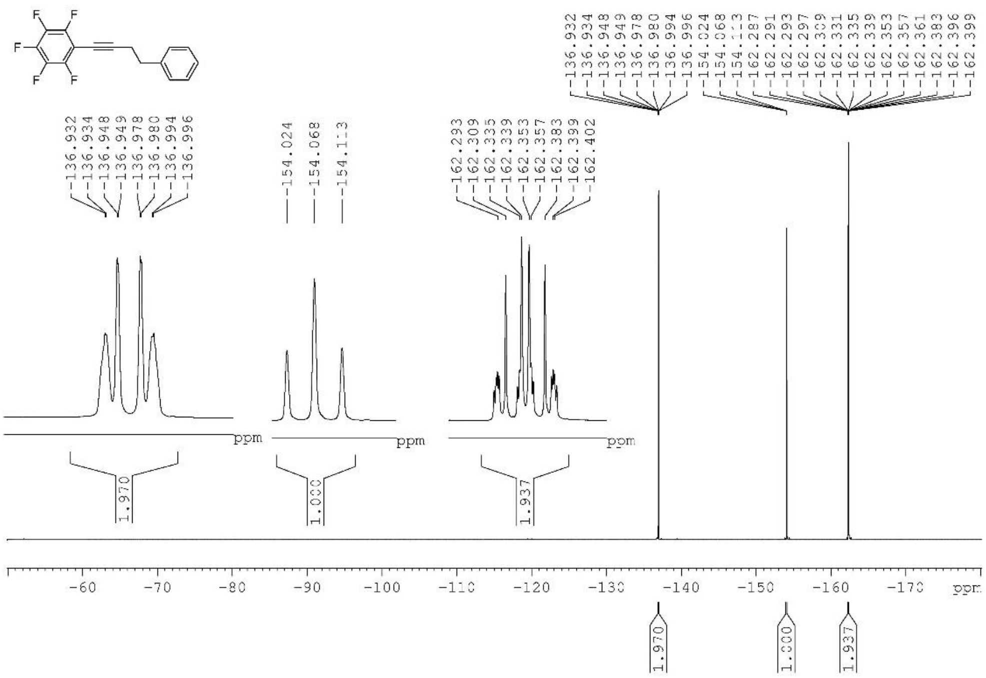

Compound 6l: ^{1}H NMR spectrum (500 MHz, $CDCl_3$).

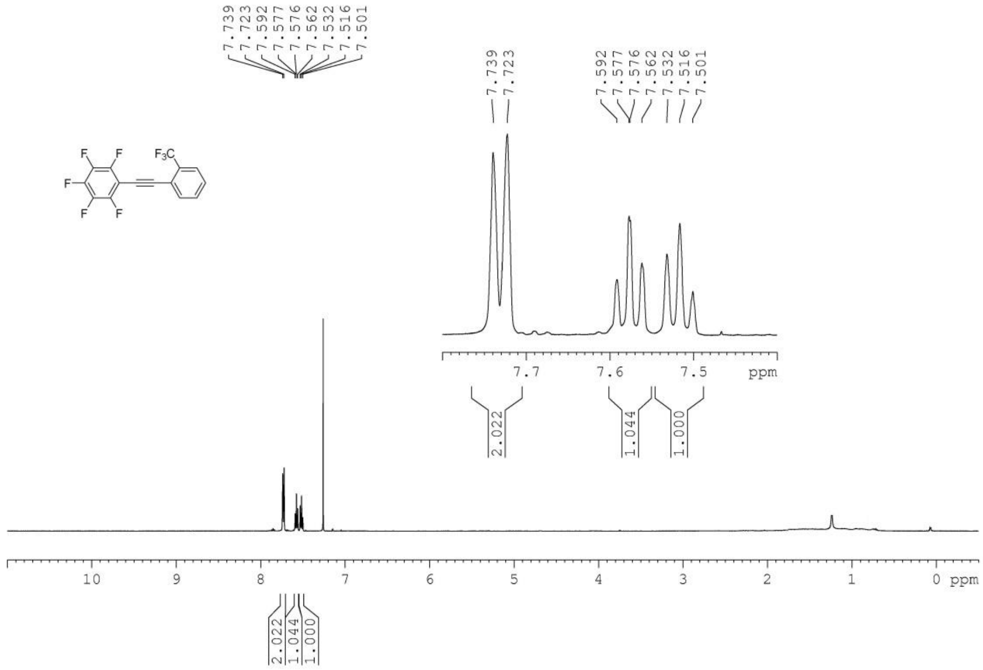

Compound 6l: $^{13}C\{^{1}H\}$ NMR spectrum (125 MHz, $CDCl_3$).

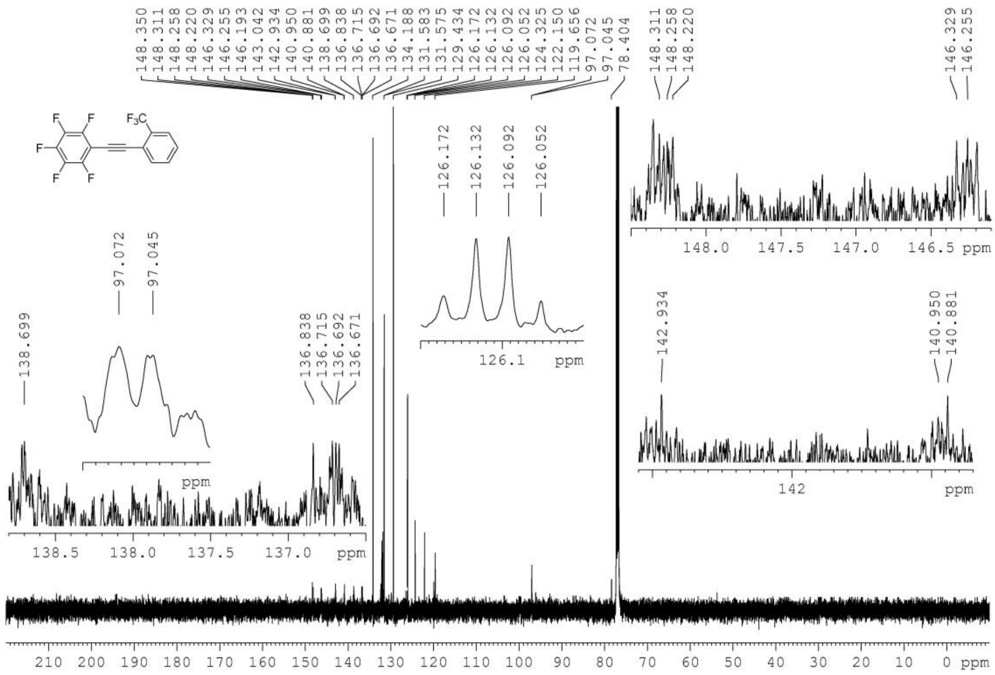

Compound 6l: ^{19}F NMR spectrum (470 MHz, $CDCl_3$).

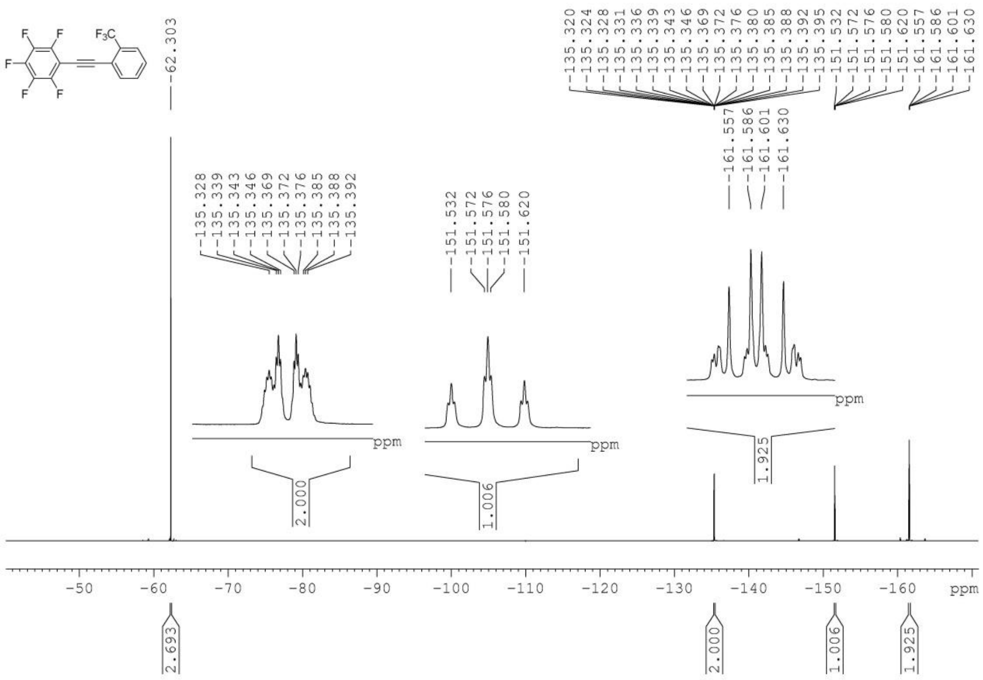

Compound 6m: ^{1}H NMR spectrum (500 MHz, $CDCl_3$).

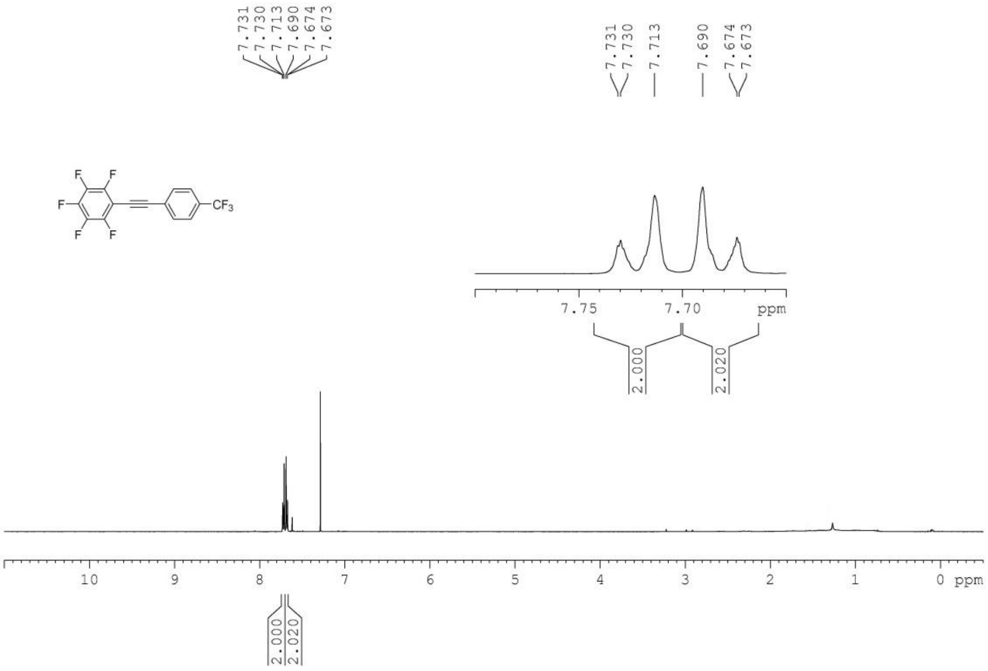

Compound 6m: $^{13}C\{^{1}H\}$ NMR spectrum (125 MHz, $CDCl_3$).

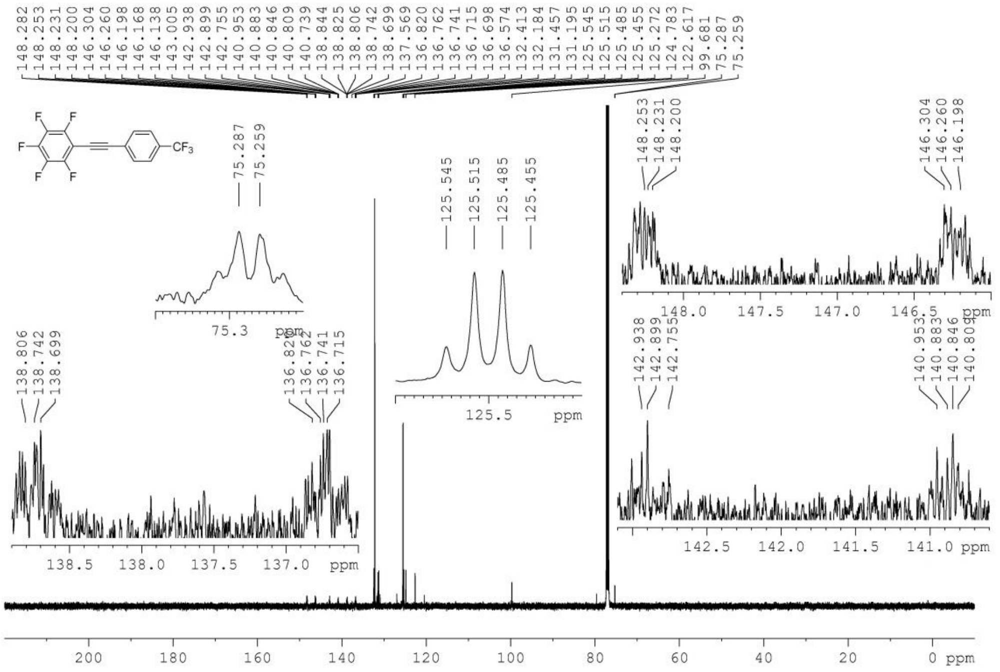

Compound 6m: ^{19}F NMR spectrum (470 MHz, $CDCl_3$).

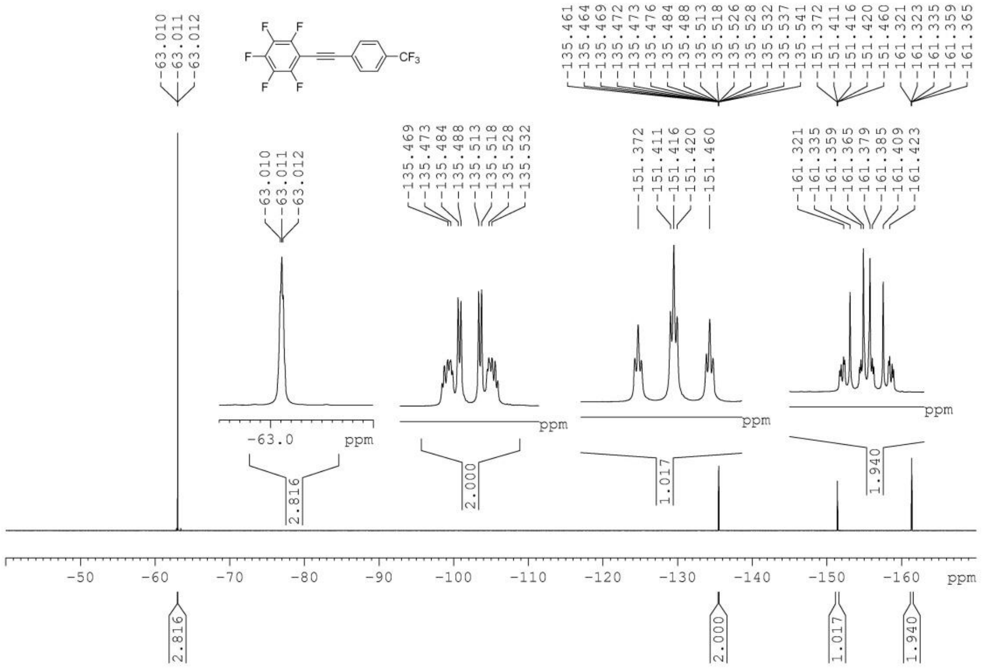

6. Crystallographic Details

Crystal structure determination. Crystals suitable for single-crystal X-ray diffraction were selected, coated in perfluoropolyether oil, and mounted on MiTeGen sample holders. Diffraction data of **6a** and **6g** were collected on Bruker X8 Apex II 4-circle diffractometers with CCD area detectors using Mo-K_{α} radiation monochromated by multi-layer focusing mirrors. The crystals were cooled using an Oxford Cryostream low-temperature device. Data were collected at 100 K. The images were processed and corrected for Lorentz-polarization effects and absorption as implemented in the Bruker software packages. The structures were solved using the intrinsic phasing method (SHELXT),[6] refined with the SHELXL program[7] using the SHELXLE graphical user interface,[8] and expanded using Fourier techniques. All non-hydrogen atoms were refined anisotropically. Hydrogen atoms were included in structure factors calculations. All hydrogen atoms were assigned to idealised geometric positions. Diamond[9] software was used for graphical representation. Other structural information was extracted using Mercury[10] and OLEX2[11] software. Crystal data and experimental details are listed in Table S1; full structural information has been deposited with the Cambridge Crystallographic Data Centre. CCDC-2000968 (**6a**) and 2000970 (**6g**).

Table S1: Single-crystal X-ray diffraction data and structure refinements of **6a** and **6g**.

Data	**6a**	**6g**
CCDC number	2000968	2000970
Empirical formula	$C_{17}H_{11}F_5$	$C_{14}H_4F_6$
Formula weight / g·mol^{-1}	310.26	286.17
T / K	100(2)	100(2)
Radiation, λ / Å	Mo-K$_\alpha$ 0.71073	Mo-K$_\alpha$ 0.71073
Crystal size / mm³	0.83 × 0.26 × 0.21	0.75 × 0.26 × 0.17
Crystal color, habit	colorless block	colorless plate
μ / mm^{-1}	0.133	0.166
Crystal system	triclinic	triclinic
Space group	$P\ \bar{1}$	$P\ \bar{1}$
a / Å	7.187(6)	5.999(2)
b / Å	8.211(4)	7.593(5)
c / Å	12.336(7)	12.626(6)
α / °	91.51(2)	83.49(2)
β / °	99.48(3)	88.664(11)
γ / °	104.99(4)	85.089(17)
Volume / Å^3	691.7(8)	569.3(5)
Z	2	2
ρ_{calc} / g·cm^{-3}	1.490	1.669
F(000)	316	284
θ range / °	2.575 − 26.512	2.999 – 26.477
Reflections collected	8583	8567
Unique reflections	2863	2359
Parameters / restraints	202 / 0	181 / 0
GooF on F^2	1.028	1.087
R_1 [I>2σ(I)]	0.0363	0.0352
wR^2 (all data)	0.1058	0.1098
Max. / min. residual electron density / e·Å^{-3}	0.286 / -0.195	0.259 / -0.224

Table S2: Intermolecular C−H···F, H···F, C···F, and F···F interaction distances (Å) and angles (°) in compounds **6a** and **6g** at 100 K less than or equal to the sum of the Van der Waals radii.

Compound	C−H···F	H···F	C/F···F	∠(CHF)
**6a**	C15−H15···F5	2.631(2)	3.536(3)	153.7(1)
	C8···C8		3.359(3)	
	C5···C14		3.294(3)	
	C16···F2		3.159(2)	
	F3···F4		2.909(2)	
**6g**	C11−H11···F4	2.6414(14)	3.496(2)	149.98(11)
	C10−H10···F5	2.5224(13)	3.316(2)	141.14(11)
	C13−H13···F6	2.5886(15)	3.420(2)	146.39(10)
	C13−H13···F2	2.5426(13)	3.213(2)	127.74(11)
	C3···C12		3.342(3)	
	C6···C9		3.399(3)	
	C3···F5		3.1262(19)	
	C6···F2		3.1440(19)	
	F2···F5		2.9205(17)	
	F3···F4		2.8556(18)	

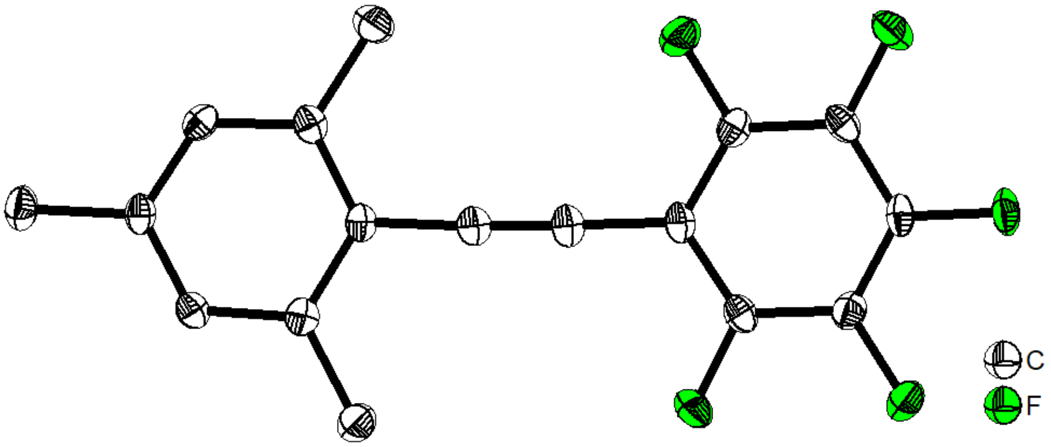

Figure S1. The solid-state molecular structure of **6a** determined by single-crystal X-ray diffraction at 100 K. All ellipsoids are drawn at the 50% probability level, and H atoms are omitted for clarity.

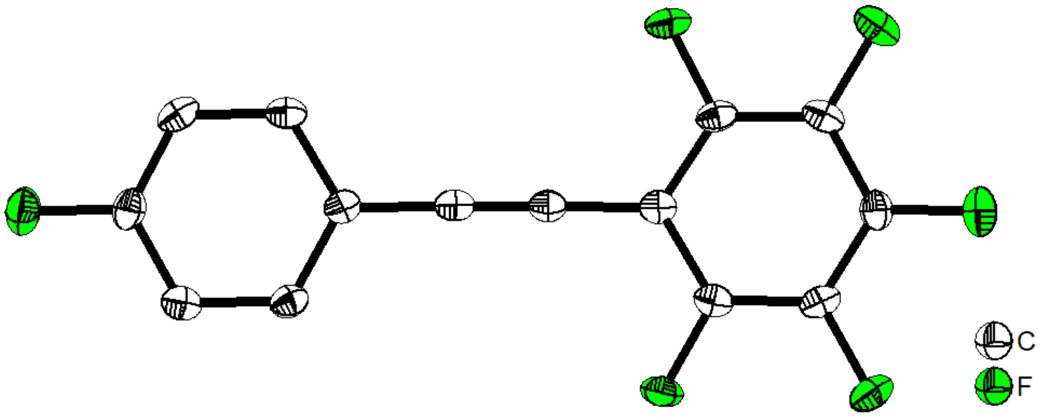

Figure S2. The solid-state molecular structure of **6g** determined by single-crystal X-ray diffraction at 100 K. All ellipsoids are drawn at the 50% probability level, and H atoms are omitted for clarity.

7. References

[1] a) T. Ishiyama, J. Takagi, K. Ishida, N. Miyaura, N. R. Anastasi, J. F. Hartwig, *J. Am. Chem. Soc*. **2002**, *124*, 390-391; b) T. Ishiyama, J. Takagi, K. Ishida, N. Miyaura, J. F. Hartwig, *Angew. Chem. Int. Ed.* **2002**, *41*, 3056-3058; c) Y. P. Budiman, A. Friedrich, U. Radius, T. B. Marder, *ChemCatChem*. **2019**, *11*, 5387-5396.

[2] N. Matsuyama, M. Kitahara, K. Hirano, T. Satoh, M. Miura, *Org. Lett.* **2010**, *12*, 2358-2361.

[3] X. Qi, J.-B. Jiang, X.-F. Wu, *Tetrahedron. Lett.* **2016**, *57*, 1706-1710.

[4] A. R. Gholap, K. Venkatasen, R. Pasricha, T. D. Rajgobal, J. Lahoti, K. V. Srinivasan, *J. Org. Chem.* **2005**, *70*, 4869-4872.

[5] Y. Wei, H. Zhao, J. Kan, W. Su, M. Hong, *J. Am. Chem. Soc.* **2010**, *132*, 2522-2523.

[6] G. M. Sheldrick, *Acta Cryst.*, **2015**, *A71*, 3-8.

[7] G. M. Sheldrick, *Acta Cryst.*, **2008**, *A64*, 112-122.

[8] C. B. Hübschle, G. M. Sheldrick, B. Dittrich, *J. Appl. Cryst*., **2011**, *44*, 1281-1284.

[9] Brandenburg, K. Diamond (version 4.4.0), Crystal and Molecular Structure Visualization, Crystal Impact H. Putz & K. Brandenburg GbR, Bonn (Germany), **2017**.

[10] C. F. Macrae, I. J. Bruno, J. A. Chisholm, P. R. Edgington, P. McCabe, E. Pidcock, L. Rodriguez-Monge, R. Taylor, J. van de Streek, P. A. Wood, *J. Appl. Cryst.*, **2008**, *41*, 466-470.

[11] O. V. Dolomanov, L. J. Bourhis, R. J. Gildea, J. A. K. Howard, H. Puschmann, *J. Appl. Cryst.*, **2009**, *42*, 339-341.
